# Public support for smoke-free policies in outdoor areas and (semi-)private places: a systematic review and meta-analysis

**DOI:** 10.1016/j.eclinm.2023.101982

**Published:** 2023-05-09

**Authors:** Nienke W. Boderie, Asiyah Sheikh, Erika Lo, Aziz Sheikh, Alex Burdorf, Frank J. van Lenthe, Famke J.M. Mölenberg, Jasper V. Been

**Affiliations:** a Department of Public Health, Erasmus MC, University Medical Centre Rotterdam, Rotterdam, Netherlands; b Edinburgh Medical School, The University of Edinburgh, Edinburgh, UK; c Centre for Medical Informatics, Usher Institute, The University of Edinburgh, Edinburgh, UK; d Division of Neonatology, Department of Neonatal and Paediatric Intensive Care, Erasmus MC Sophia Children’s Hospital, University Medical Centre Rotterdam, Rotterdam, Netherlands; e Department of Obstetrics and Gynaecology, Erasmus MC Sophia Children’s Hospital, University Medical Centre Rotterdam, Rotterdam, Netherlands

**Keywords:** Tobacco smoke pollution, Tobacco smoking, Child, Public opinion, Surveys and questionnaires, Systematic review, Meta-analysis, Policy making, Smoke-free policy

## Abstract

**Background:**

Smoke-free policies are essential to protect people against tobacco smoke exposure. To successfully implement smoke-free policies that go beyond enclosed public places and workplaces, public support is important. We undertook a comprehensive systematic review of levels and determinants of public support for indoor (semi-)private and outdoor smoke-free policies.

**Methods:**

In this systematic review and meta-analysis, six electronic databases were searched for studies (published between 1 January 2004 and 19 January 2022) reporting support for (semi-)private and outdoor smoke-free policies in representative samples of at least 400 respondents aged 16 years and above. Two reviewers independently extracted data and assessed risk of bias of individual reports using the Mixed Methods Appraisal Tool. The primary outcome was proportion support for smoke-free policies, grouped according to location covered. Three-level meta-analyses, subgroup analyses and meta-regression were performed.

**Findings:**

14,749 records were screened, of which 107 were included; 42 had low risk of bias and 65 were at moderate risk. 99 studies were included in the meta-analyses, reporting 326 measures of support from 896,016 individuals across 33 different countries. Support was pooled for indoor private areas (e.g., private cars, homes: 73%, 95% confidence interval (CI): 66–79), indoor semi-private areas (e.g., multi-unit housing: 70%, 95% CI: 48–86), outdoor hospitality areas (e.g., café and restaurant terraces: 50%, 95% CI: 43–56), outdoor non-hospitality areas (e.g., school grounds, playgrounds, parks, beaches: 69%, 95% CI: 64–73), outdoor semi-private areas (e.g., shared gardens: 67%, 95% CI: 53–79) and outdoor private areas (e.g., private balconies: 41%, 95% CI: 18–69). Subcategories showed highest support for smoke-free cars with children (86%, 95% CI: 81–89), playgrounds (80%, 95% CI: 74–86) and school grounds (76%, 95% CI: 69–83). Non-smokers and ex-smokers were more in favour of smoke-free policies compared to smokers. Support generally increased over time, and following implementation of each smoke-free policy.

**Interpretation:**

Our findings suggested that public support for novel smoke-free policies is high, especially in places frequented by children. Governments should be reassured about public support for implementation of novel smoke-free policies.

**Funding:**

10.13039/100002129Dutch Heart Foundation, 10.13039/501100014780Lung Foundation Netherlands, 10.13039/501100004622Dutch Cancer Society, 10.13039/501100003092Dutch Diabetes Research Foundation and 10.13039/501100012028Netherlands Thrombosis Foundation.

**Research in context**

**Evidence before this study**

Smoke-free policies can protect people against the harmful effects of tobacco smoke exposure. When implementing such policies, information on public support is essential to policy makers. We identified any existing or planned systematic reviews investigating public support for smoke-free policies in indoor (semi-)private and outdoor (semi-)private areas, i.e., “novel” smoke-free policies. Google Scholar was searched on 23 January 2020 using the terms: “systematic review”, “meta-analysis”, “smoking”, “policy”, “regulation”, “legislation”, “law”, “outdoor”, “private”, “support”. We identified five systematic or scoping reviews focusing on support for specific smoke-free locations, or within specific countries showing high or increasing levels of support for outdoor smoking regulations, smoke-free multi-unit housing and smoke-free cars.

**Added value of this study**

Our systematic review and meta-analysis provides the first comprehensive overview of support for smoke-free policies that go beyond smoke-free indoor public places and workplaces without any language or geographic restrictions. Overall, the findings from over 100 studies of which 99 could be included in meta-analysis, indicate that public support for novel smoke-free policies is generally high, particularly in areas where children are commonly exposed to tobacco smoke.

**Implications of all the available evidence**

Smoke-free policies can benefit health and more and more countries are implementing smoke-free policies in novel places. Governments should be reassured by the considerable public support for implementation of smoke-free policies in (semi-)private areas and outdoor public places.

## Introduction

Exposure to second-hand smoke (SHS) poses a major burden to population health globally. Each year, 1.2 million deaths and 36.3 million disability-adjusted life years (DALYs) are attributed to SHS exposure.^1^ Compelling evidence indicates that comprehensive legislation to protect non-smokers from tobacco smoke exposure in all indoor public places and workplaces is a powerful tool to reduce the adverse effects of tobacco smoke, including among children.^1^, ^2^, ^3^ In an attempt to further improve population health, an increasing number of jurisdictions have expanded smoke-free policies to encompass outdoor places (e.g., public parks, pedestrian plazas and beaches),^4^ semi-private places (e.g., public housing units),^5^ and private places (e.g., cars).^6^ Emerging evidence shows that such “novel” policies can indeed be effective in further reducing the burden of SHS exposure in children.^7^^,^^8^

Public support is important for policymakers to consider implementing novel smoke-free policies and to maximise compliance.^9^ The World Health Organization (WHO) stated in their 2009 report on the global tobacco epidemic that ‘involving civil society is central to achieving effective legislation’.^10^ Previous literature has shown large differences in public support between various smoke-free places and within populations. For example, in the USA and Canada playgrounds generally received a higher degree of support compared to sidewalks, and smokers are generally less in favour of smoke-free policies than non-smokers.^11^ To inform policy-making regarding extending smoke-free policies, it is thus important to derive insights into the levels and determinants of public support for smoke-free policies that cover outdoor areas and (semi-)private places.

Therefore, the primary objective of this study was to systematically review evidence on the levels and determinants of public support for smoke-free policies covering outdoor places or (semi-)private places, henceforth referred to as ‘novel smoke-free policies’. To our knowledge this is the first comprehensive overview of support for novel smoke-free policies that is not limited to a specific smoke-free place or geographic region. Our secondary objective was to identify which personal and country-level characteristics are associated with public support for these policies.

## Methods

### Search strategy and selection criteria

We undertook a systematic review and meta-analysis in accordance with our peer-reviewed review protocol.^12^ As we did not have a clinical outcome, PROSPERO considered our protocol ineligible for registration. Our review is reported according to the Preferred Items for Systematic Reviews and Meta-Analyses (PRISMA) guidelines. Six electronic databases were searched (Embase.com, Medline Ovid, Web of Sciences, Cochrane, CINAHL database and PsychINFO) for reports published between 1 January 2004 and 19 January 2022 that reported public support for novel smoke-free policies (see Appendix I for the complete list of search terms). The search was conducted on 17 March 2020 and updated 19 January 2022. No restrictions were applied for language; studies were translated using Google Translate if needed. Additional relevant reports were included through reference and citation screening of included papers.^13^ All records were extracted into EndNote (EndNote X9, Thomson Reuters, New York, USA) and automatically and manually de-duplicated. Two reviewers (NWB and AsS) independently screened all titles and abstracts, and subsequently the full texts, to identify eligible studies. Any discrepancies were resolved via involving a third reviewer (JVB).

### Eligibility

Eligible studies investigated support for smoke-free policies in indoor private places (e.g., cars, homes), indoor semi-private places (e.g., multi-unit housing), outdoor semi-private places (e.g., shared gardens), outdoor hospitality places (e.g., terraces of bars and restaurants), or outdoor non-hospitality places (e.g., playgrounds, streets, beaches). Policies could already have been in place, were planned, or were hypothetical (e.g., before plans for implementation have started). Studies were only eligible when support was assessed in a population aged 16 years or above, representing the majority of a population affected by the policy (e.g., support for smoke-free university campus assessed among university students). Studies were excluded if: 1) fewer than 400 participants were included; this limit is based on survey sample size calculations to ensure a margin of error of 5%,^12^ 2) support was only reported for indoor public places or workplaces, 3) the policy only covered non-combustible tobacco products (e.g., electronic cigarettes or heat-not-burn tobacco products), or 4) published before 1 January 2004. This pragmatic cut-off was chosen because the first national ‘traditional' smoke-free law (i.e., covering indoor public places and workplaces) was introduced in Ireland in 2004. Finally, studies reporting support among groups with clearly vested interests such as tobacco industry groups were excluded.

### Data extraction

Three authors independently performed the data extraction (NWB, AsS and EL), and cross-checked one another. The following information was extracted: first author’s name, publication year, type of publication, study design, location of the study, description of the policy, policy information (implementation date, level of implementation and level of enforcement), observational period, selection of participants (eligibility, sampling method), method of data collection, definition of support, method of assessment (dichotomised or Likert-scale question), statistical analysis (if applicable), numbers and percentages of missing values and non-response (if applicable), techniques for handling missing data, level of support (estimate, 95% confidence interval), personal characteristics (age, gender, smoking status, and parental status of participants), any conflict of interest reported by the authors and funding sources. Furthermore, information was extracted regarding whether the degree of support was for a hypothetical scenario or for a policy that was (about to be) implemented. For reports including pre- and post-implementation support, information was sought on all measurement points.

Country income level according to World Bank classifications and information on traditional comprehensive smoke-free legislation in place according to WHO was sought externally for each report.^14^^,^^15^

### Risk-of-bias assessment

The Mixed Methods Appraisal Tool (MMAT)^16^ was used to assess risk-of-bias of the included studies, which includes assessment of the relevance of the sampling strategy, representativeness of the target population, appropriateness of the outcome measures, risk of non-response bias, and the appropriateness of statistical methods.

### Data synthesis

Reported support was expressed as the proportion of the surveyed population endorsing the smoke-free policy. When studies reported the proportion that was *not* in favour of the policy outcome estimates were reversed. If Likert scales were used, all answer options that were more positive than neutral were combined to indicate support. Policies were categorised by the places that they cover: 1) indoor private places, 2) indoor semi-private places, 3) outdoor private places, 4), outdoor semi-private places, 5) outdoor hospitality places, and 6) outdoor non-hospitality places. When multiple estimates per category were presented (e.g., for outdoor eating places and for outdoor café places within outdoor hospitality places), the average support across the category was calculated. For studies based on the same samples a hierarchy of criteria was used to include one of them; the included study was most representative of the general population, had the lowest risk of bias, was based on the largest sample size. If relevant data were missing, corresponding authors were contacted.

### Data analysis

All analyses were conducted with R V.3.6.5 (R Foundation for Statistical Computing, 2020). Support reported as proportions ranging between 0 and 1 did not meet the normality assumption, and therefore logit transformations were applied.^17^ If support was reported as the mean score of a Likert scale ranging between 1 and a maximum score, this was transformed to the proportion support using the following formula:

$$proportion\;support=\frac{Mean\;score-1}{Highest\;scale\;value-1}\text{.}$$

If the Likert scale ranged between 0 and a maximum score, the mean score was divided by the highest scale value. For ease of interpretation, proportions were converted to percentage support. We assumed support was related to country of residence and thus violated the meta-analysis independence assumption if multiple studies from a single country were included. Hence, a three-level meta-analysis was conducted to account for within-study, between-study and country-level clustering.^18^ The metafor package version 3.0.2 was used, which applies inverse variance weighting and accounts for dependence between the estimates.^19^

Subgroup analyses were conducted by gender (men vs. women; none of the studies reported data according other categories), smoking status (smokers vs. non-smokers, and smokers vs. former smokers), parental status (parents vs. others), and age group (youngest age group vs. oldest age group reported). Log odds ratios (ORs) were pooled; if ORs were not reported the following formula was used:

$$OR=\frac{Support\;group\;A}{1-support\;group\;A}/\frac{Support\;group\;B}{1-support\;group\;B}$$

Finally, pooled log ORs were back-transformed to ORs for ease of interpretation.

In addition to the subgroup analysis three-level meta-regression analysis was conducted. Variables of interest were: calendar year in which the study was conducted, country income level (high- vs. low- and middle-income (LIMC)), and the comprehensiveness of traditional smoke-free policies in place (range: 0—no smoke-free policies in place to 8—all public places completely smoke-free, based on WHO criteria).^15^ Additional post-hoc analyses were performed to assess differences in support over time by four-year interval groups and for hypothetical policies (i.e., no plans existing for future implementation) versus non-hypothetical policies.

Finally, a sensitivity analysis to explore the impact of risk-of-bias of individual studies on the findings was performed by repeating the meta-analyses separately for studies with low risk of bias and studies at higher risk of bias (i.e., studies that had *no* or *can’t tell* on at least one of the MMAT criteria). As appropriate for proportional data, publication bias was assessed using funnel plots of study size against log odds.^20^^,^^21^

### Ethics

As we only included previously published studies we did not seek ethical approval.

### Role of the funding source

The funders were not involved in writing the manuscript or the decision to submit for publication. All authors interpreted the analyses, read and approved the final manuscript, had full access to all the data in the study and had final responsibility for the decision to submit for publication.

## Results

**Fig. 1:**
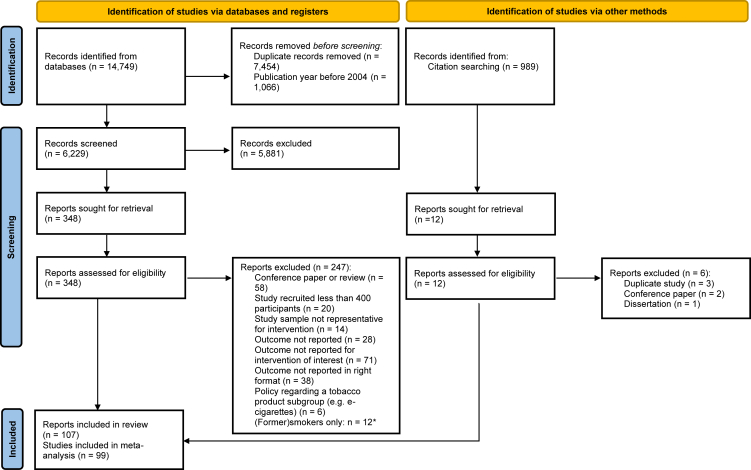
**PRISMA flow diagram.** A record refers to an entry in an electronic database describing a report. A report is a full text published research article and there may be one or more reports describing individual research projects. ∗Reports on support among (former) smokers only can be found in Appendix III.

14,749 records were identified from the databases. Duplicates and reports published before 2004 were omitted, resulting in 6229 unique records to screen. Full-text records were assessed for 348 records and 107 reports were included (Fig. 1).^22^, ^23^, ^24^, ^25^, ^26^, ^27^, ^28^, ^29^, ^30^, ^31^, ^32^, ^33^, ^34^, ^35^, ^36^, ^37^, ^38^, ^39^, ^40^, ^41^, ^42^, ^43^, ^44^, ^45^, ^46^, ^47^, ^48^, ^49^, ^50^, ^51^, ^52^, ^53^, ^54^, ^55^, ^56^, ^57^, ^58^, ^59^, ^60^, ^61^, ^62^, ^63^, ^64^, ^65^, ^66^, ^67^, ^68^, ^69^, ^70^, ^71^, ^72^, ^73^, ^74^, ^75^, ^76^, ^77^, ^78^, ^79^, ^80^, ^81^, ^82^, ^83^, ^84^, ^85^, ^86^, ^87^, ^88^, ^89^, ^90^, ^91^, ^92^, ^93^, ^94^, ^95^, ^96^, ^97^, ^98^, ^99^, ^100^, ^101^, ^102^, ^103^, ^104^, ^105^, ^106^, ^107^, ^108^, ^109^, ^110^, ^111^, ^112^, ^113^, ^114^, ^115^, ^116^, ^117^, ^118^, ^119^, ^120^, ^121^, ^122^, ^123^, ^124^, ^125^, ^126^, ^127^, ^128^ The included reports presented estimates of public support for novel smoke-free places from 33 different countries. Appendix II reports the geographic location, the smoke-free place, measure of support, sample size, and the reported support per study.

Most reports (n = 67) assessed support for hypothetical scenarios, i.e., support for smoke-free places not yet in place without referring to concrete plans for implementation or actual implementation (see Appendix III).^22^, ^23^, ^24^, ^25^, ^26^, ^27^^,^^29^^,^^30^^,^^33^^,^^34^^,^^39^^,^^42^^,^^43^^,^^47^, ^48^, ^49^, ^50^, ^51^^,^^56^, ^57^, ^58^^,^^60^^,^^61^^,^^63^, ^64^, ^65^, ^66^, ^67^, ^68^^,^^71^^,^^72^^,^^74^^,^^75^^,^^77^, ^78^, ^79^^,^^81^^,^^83^^,^^85^, ^86^, ^87^^,^^89^, ^90^, ^91^, ^92^, ^93^^,^^95^^,^^99^^,^^100^^,^^102^, ^103^, ^104^, ^105^, ^106^^,^^108^^,^^110^^,^^112^^,^^115^^,^^116^^,^^119^^,^^120^^,^^123^, ^124^, ^125^, ^126^^,^^128^^,^^129^ Four reports assessed support for smoke-free policies that were likely to be implemented,^107^ or for possible extensions of—or additions to—existing policies.^54^^,^^105^^,^^118^ Thirty-six reports assessed public support for policies that were already implemented (Appendix IV), among which five were introduced at the national level: smoke-free cars with children or pregnant women in Italy^82^; a smoke-free prison policy in Scotland^37^^,^^114^; and a smoke-free public housing act in the USA.^88^^,^^121^ In the remaining thirty-one studies support for local smoke-free policies was assessed regarding university or college campuses,^31^^,^^35^^,^^38^^,^^41^^,^^52^^,^^76^^,^^84^^,^^97^^,^^109^^,^^127^ school grounds,^40^^,^^113^ hospital grounds,^40^^,^^59^^,^^80^^,^^117^^,^^122^ playgrounds,^45^^,^^113^ parks and beaches,^28^^,^^54^^,^^73^^,^^94^^,^^107^^,^^111^^,^^118^ multi-unit housing,^44^^,^^46^^,^^70^ and outdoor gathering places such as streets.^28^^,^^32^^,^^98^

### Risk-of-bias assessment

Risk-of-bias assessment of individual reports is reported in Appendix V. Forty-two reports scored ‘yes’ on all MMAT criteria and were therefore considered to have low risk of bias, 65 reports had at least one ‘no’ or ‘can’t tell’ and were therefore considered to have a moderate risk of bias.

### Meta-analysis

Eight reports were not included in meta-analyses: three because public support estimates from the same study population in more recent years were available,^36^^,^^37^^,^^77^ one report because the outcome measure included a combined score for novel and traditional policies,^62^ one report because it only reported support combined for multiple countries,^125^ one report because it did not present the sample size,^128^ and two due to overlapping samples.^69^^,^^96^ Lund et al. also overlapped, expect for one estimate on outdoor hospitality and was therefore still included.^83^ Public support for smoke-free policies was pooled for six main categories of places, which were further categorised into 15 subgroups. Table 1 provides an overview of the number of studies and their combined sample sizes per category of smoke-free places. The majority of countries in which the studies were conducted had traditional smoke-free legislation in place covering four to five out of eight public places. Likert scale questions were more frequently used than binary questions to assess support.

**Table 1:** Descriptive statistics of 99 studies with 326 estimates of support for novel smoke-free policies included in the meta-analysis.

Places of smoke-free policies	Estimates of support across studies^a^ (n)	Sample size (n)	High income country (%)^b^	Number of WHO recommended smoke-free policies in place^c^*Median (IQR)*	Likert scale question (%)^d^	Hypothetical question (%)^e^
**Indoor private**	**61**	**950,436**	**80**	**6 (7)**	**100**	**95**
Cars with children	30	518,621	83	6 (7)	100	90
Cars	22	419,449	86	8 (5)	100	100
Homes	9	12,366	56	1 (2)	100	100
**Indoor semi-private**	**25**	**35,447**	**79**	**1 (2)**	**88**	**71**
Multi-unit housing	17	25,639	82	1 (2)	88	71
Other semi-private	7	9754	71	2 (6)	88	71
**Outdoor hospitality**	**24**	**114,062**	**75**	**6 (5)**	**100**	**96**
**Outdoor non-hospitality**	**208**	**867,344**	**79**	**6 (7)**	**97**	**77**
Areas surrounding building entrances	8	96,653	62	5 (6)	100	75
Areas surrounding health care facilities	24	32,628	91	8 (4)	100	67
Event locations	24	39,367	83	6 (4)	96	92
Parks & beaches	30	143,599	80	7 (6)	97	77
Playgrounds	20	249,127	80	7 (3)	100	90
Streets or open areas	7	12,101	71	6 (6)	86	38
Public transport stops	24	36,898	79	6 (5)	100	80
School terrains	23	104,307	78	6 (5)	96	87
University campus	38	77,534	76	1 (7)	95	74
Other outdoor areas	9	76,601	67	3 (6)	89	50
**Outdoor private**	**3**	**10,862**	**100**	**1 (4)**	**100**	**67**
**Outdoor semi-private**	**5**	**5549**	**40**	**3 (2)**	**100**	**100**

Abbreviations: World Health Organization (WHO); interquartile range (IQR).

a Estimates are the total number of estimates for public support across studies. One study can provide multiple estimates.

b We used the World Bank categorisation, and presented are the percentage of estimates for public support derived from high-income countries.

c Number of enclosed public places covered by traditional smoke-free legislation in place according to WHO classification ranging from 0 to 8 (i.e.,: health care facilities, educational facilities, universities, governmental facilities, indoor private offices and workplaces, restaurants, pubs and bars, and public transport).^130^ We used the data from the year that public support was measured.

d Percentage of estimates for public support on a Likert-scale vs. binary scale.

e Percentage of estimates for public support for hypothetical vs. implemented smoke-free places.

**Fig. 2:**
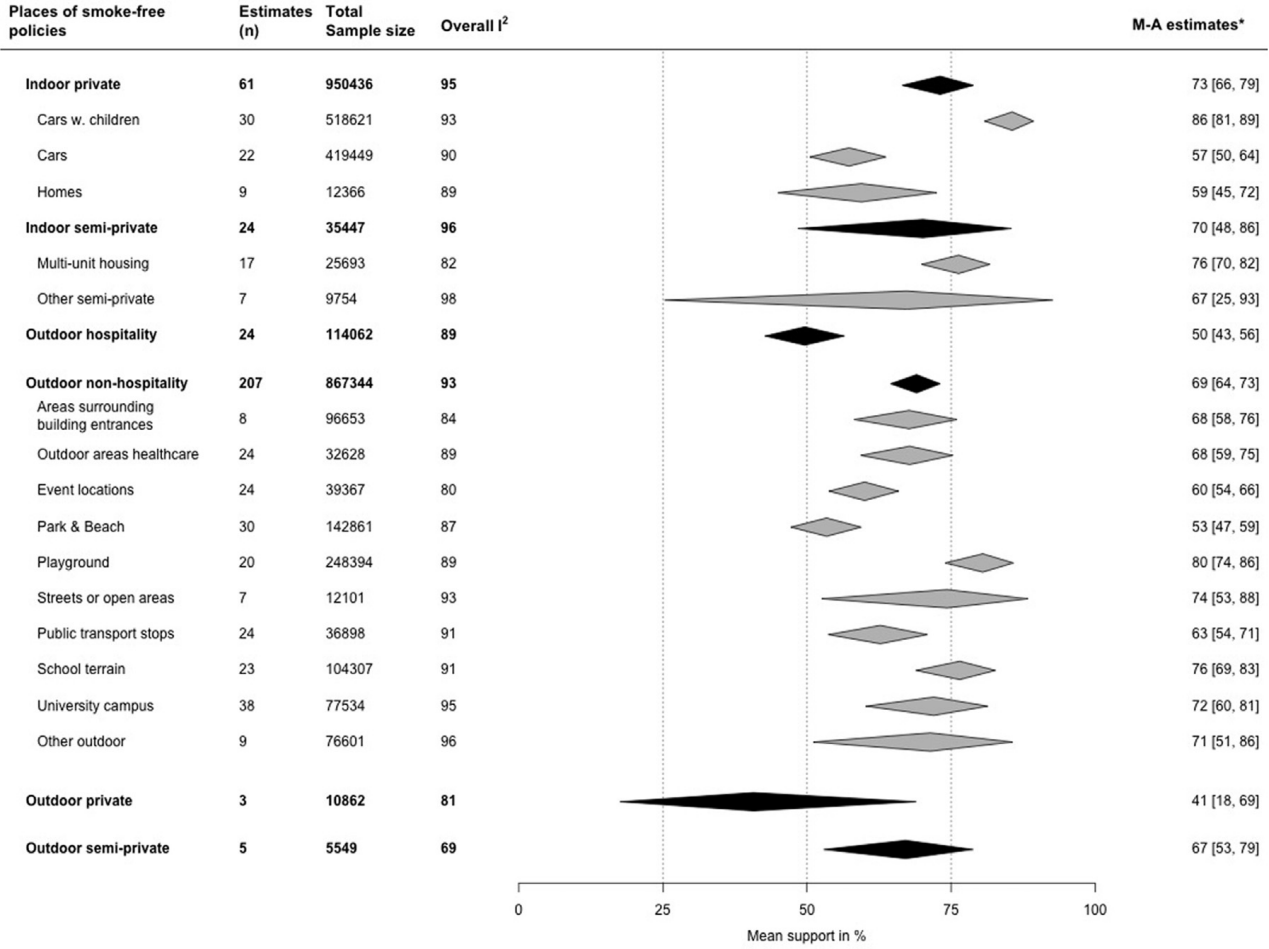
**Summary of pooled support estimates for six categories of smoke-free places, and 15 subcategories.** Pooled support was obtained from three-level random effects meta-analysis. ∗Each estimate represents a separate meta-analysis.

In total, data from 896,016 participants were included in the meta-analyses. Some studies provided multiple estimates of public support for policies covering different places, therefore the total number of observations was 1,938,700. Fig. 2 shows the pooled estimates of support per category and subcategory of novel smoke-free policies. Forest plots for each meta-analysis are provided in Appendix VI and explained variance per meta-analysis in Appendix VII. The highest level of support for smoke-free places was found for indoor private places (73%, 95% CI: 66–79; 61 estimates; 950,436 observations), followed by indoor semi-private places (70%, 95% CI: 48–86; 25 estimates; 35,447 observations). Pooled public support was 69% for outdoor non-hospitality places (95% CI: 65–73; 208 estimates; 867,344 observations) and 67% for outdoor semi-private places (95% CI: 53–79; 5 estimates; 5549 observations). For outdoor hospitality places pooled support was 50% (95% CI: 43–56; 24 estimates; 114,062 observations) and lowest pooled support was found for outdoor private places with 41% (95% CI: 18–69; 3 estimates; 10,862 observations).

Support was highest for policies making cars carrying children smoke-free (86%, 95% CI: 81–89; 30 estimates; 518,621 observations), followed by playgrounds (80%, 95% CI: 72–86; 21 estimates; 249,127 observations) and school grounds (76%, 95% CI: 69–83; 23 estimates; 104,307 observations). For all subcategories except outdoor private areas, we found a mean pooled support higher than 50%. Places with relatively low levels of support included: parks and beaches (53%, 95% CI: 47–59; 30 estimates; 143,599 observations), outdoor hospitality places (50%, 95% CI: 43–56; 24 estimates; 114,062 observations) and outdoor private places (41%, 95% CI: 18–69; 3 estimates; 10,862 observations).

### Heterogeneity

Heterogeneity was assessed within (level 1) and between (level 2) studies, and between countries (level 3, Appendix VII). Overall heterogeneity was 69% or higher for all types of locations, indicating substantial heterogeneity across the three levels.^131^

### Subgroup analyses

**Fig. 3:**
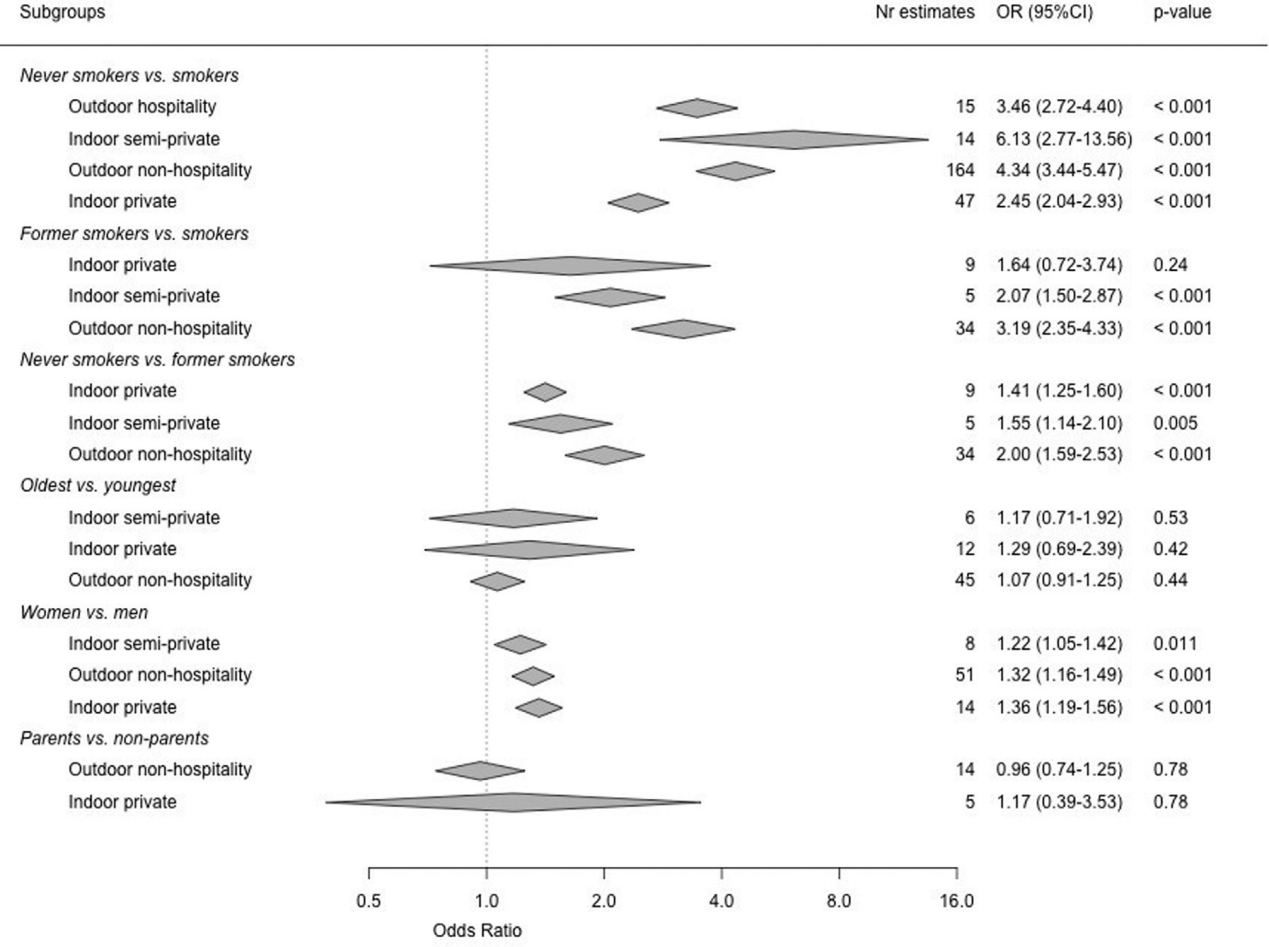
**Summary of pooled support estimates for places of smoke-free policies, by gender, smoking status, age group, and parental status.** Pooled support was obtained from three-level random effects meta-analysis.

Support was almost uniformly significantly higher among non-smokers and former smokers than among current smokers with ORs ranging between 2.45 and 6.13 (Fig. 3). Non-smokers showed higher support than former smokers, although these differences were more modest (OR range between 1.64 and 3.19). No significant differences in support were observed between the youngest and oldest age groups and between parents and those without children. Compared to men, women were slightly but significantly more often in favour of novel smoke-free policies.

### Meta-regression

Meta-regression analyses indicated no significant associations between question type, study year, and number of indoor public places covered by smoke-free legislation and support for novel smoke-free policies (Table 2). People from LMICs generally had comparable levels of support compared to those from high income countries, except for outdoor non-hospitality policies, where support was substantially higher in LMICs (OR = 2.12, 95% CI: 1.17–3.68). In post-hoc analyses support was higher for indoor-private and outdoor hospitality smoke-free policies when they were planned or already in place, as compared to when not yet planned or implemented. For indoor semi-private policies, the opposite was true (Appendix VIII).

**Table 2:** Meta-regression analysis per category of smoke-free place based on 99 studies with 326 estimates of support.

Areas of smoke-free policies	Variable	OR	95% CI	p-value
Indoor private (n = 58)				
	Study year, per year increase	1.01	(0.93–1.11)	0.744
	Number of smoke-free policies in place	0.97	(0.86–1.08)	0.554
	Low- and middle-income country^a^	1.23	(0.44–3.39)	0.687
Indoor semi-private (n = 24)				
	Study year, per year increase	1.08	(0.97–1.20)	0.152
	Number of smoke-free policies in place	1.14	(0.86–1.52)	0.329
	Low- and middle-income country^a^	1.19	(0.11–12.92)	0.880
	Question type^b^	2.40	(0.94–6.14)	0.066
Outdoor hospitality (n = 24)				
	Study year, per year increase	0.93	(0.85–1.01)	0.086
	Number of smoke-free policies in place	1.08	(0.97–1.20)	0.170
	Low- and middle-income country^a^	1.14	(0.53–2.44)	0.723
Outdoor non-hospitality (n = 204)				
	Study year, per year increase	0.97	(0.92–1.02)	0.216
	Number of smoke-free policies in place	1.02	(0.95–1.09)	0.576
	Low- and middle-income country^a^	2.19	(1.25–3.86)	0.006
	Question type^b^	1.06	(0.42–2.67)	0.904

a Versus high-income country (using World Bank criteria).

b Binary question.

### Public support for smoke-free policies following implementation

We identified 12 studies that evaluated support for smoke-free policies before and after implementation (Table 3). Only one study used a controlled design with a control and intervention location.^31^ Six studies found that public support significantly increased following the introduction of smoke-free policies,^31^^,^^45^^,^^59^^,^^76^^,^^122^^,^^127^ and six studies reported no significant change in support.^52^^,^^70^^,^^101^^,^^114^^,^^117^^,^^118^ Increasing public support following implementation was found for smoke-free public transportation stops and children’s playgrounds,^45^ a smoke-free policy on grounds and parking places of an Australian health service,^59^ university campuses,^31^^,^^76^^,^^127^ and hospital campuses.^122^ Two studies showed a decrease in support directly after implementation of a policy, which subsequently increased at follow-up.^70^^,^^114^ Among smokers support also increased over time in all but one study.

**Table 3:** Public support following implementation of smoke-free policies from 12 studies investigating pre and post implementation support.

Author	Places covered by smoke-free policy	Measurements and comparison	Findings	Support (percentage, unless otherwise specified)
Berg (2020)	University campus	1 pre- and 1 post-measurement among an intervention and control campus	Support for smoke-free university campuses significantly increased following their introduction, while no changes were seen at the control campus. Support was measured on a 1 to 5 scale, where lower scores indicate a more favourable view towards a smoke-free campus favourable towards smoking.	Mean (SD) Campus with policy: Pre: 2.52 (1.50) Post: 1.71 (0.95) Campus without policy: Pre: 2.59 (1.55) Post: 2.63 (1.43)
Dono (2014)	Public transport stops and playgrounds	2 pre- and 1 post-measurement for public transport stops 1 pre- and 1 post-measurement for children’s playgrounds	Support for smoke-free public transport stops and children’s playgrounds significantly increased following their introduction, for smokers as well as non-smokers.	Pre (2003): 79.6% Pre (2005): 78.3% Post (2013): 93.5% Odds ratio (OR) for post compared to pre intervention level of support OR: 3.8 95% CI (3.0–4.7) Pre (2005): 94.8% Post (2013): 97.8% OR: 2.5 95% CI (1.8–3.6)
Farran (2021)	University campus	1 pre- and 1 post-measurement	Support for smoke-free university campuses did not significantly change following their introduction, for smokers as well as non-smokers.	Smokers: Pre: 66.0% Post: 73.2% Non-smokers: Pre: 91.5% Post: 94.6%
Hale (2017)	Health care grounds	2 post-measurements	Support for smoke-free health care grounds significantly increased following their introduction.	After 6 months: 70% After 3 years: 74% OR for 6 months vs. 3 years after implementation OR: 1.25 95% CI (1.02–1.52)
Kennedy (2015)	Multi-unit housing	1 pre- and 2 post- measurements	Among smokers, support for smoke-free multi-unit housing significantly decreased directly after implementation, but increased again 2 years later. Among non-smokers, support for smoke-free multi-unit housing did not significantly change at both time-points.	Smokers: Pre: 26.0% After 1 year: 22.9% After 3 years: 29.4% Non-smokers: Pre: 86.7% After 1 year: 88.5% After 3 years: 88.2%
Lechner (2012)	University campus	1 pre- and 3 post-measurements	Support for smoke-free university campuses significantly increased following their introduction. Support was measured on a score 1 to 7, where higher is more favourable towards smoke-free environment.	Mean (SD) Pre: 4.57 (2.43) After 1 year: 5.33 (2.22) After 2 years: 5.47 (2.04) After 3 years: 5.77 (1.93)
Riad-Allen (2017)	Hospital campus	1 pre- and 2 post-measurements	Support for hospital campuses did not significantly change following their introduction. Support was measured on a score 1 to 7, where higher is more favourable towards smoke-free environment.	Mean (SD) Pre: 3.89 (1.31) After 6 months: 3.75 (1.16) After 1 year: 3.87 (1.34)
Sweeting (2021)	Prison	2 pre- and 1 post measurement	Support for smoke-free prisons did not significantly change over time, for prisoners as well as staff.	Prisoners: Hypothetical: 23.5% Pre: 25.0% Post: 27.3% Staff: Hypothetical: 79.0% Pre: 69.9% Post: 83.7%
Unrod (2012)	Campus (both indoor and outdoor areas)	1 pre- and 1 post-measurement	Support for smoke-free campuses did not significantly change following their introduction, for smokers as well as non-smokers.	Non-smokers: Pre: 86.0% Post: 89.7% Smokers: Pre: 19.8% Post: 16.7%
Waddell (2014)	Parks and beaches	2 pre- and 1 post-measurement	Support for smoke-free public parks and beaches did not significantly change following their introduction.	Public parks: 2010: 52% 2011: 46% Post: 47% Public beaches: 2010: 48% 2011: 44% Post: 50%
Wheeler (2007)	Hospital campus	1 pre- and 1 post-measurement	Support for smoke-free hospital campuses significantly increased following their introduction.	Pre: 83.3% Post: 89.9%
Wray (2020)	University campus	1 pre- and 1 post-measurement	Support for smoke-free university campuses significantly increased following their introduction.	Pre: 75% Post: 84%

### Sensitivity analyses

Pooled estimates for studies at high risk vs. low risk of bias showed that support for policies concerning outdoor private places was higher among studies with a low risk of bias (see Appendix IX). Most higher-risk studies lacked information on the question “*Is the risk of non-response bias low?”*. Funnel plots of sample size against log odds were not suggestive of publication bias (Appendix X).

## Discussion

This systematic review and meta-analysis including data from almost 900,000 unique participants from 33 different countries indicates high levels of support for smoke-free policies in the majority of outdoor areas and (semi-)private places. Support was particularly high for smoke-free places where children are commonly exposed to tobacco smoke, such as cars carrying children, playgrounds, and school grounds. Except for outdoor private areas, support was 50% or higher for all places evaluated. Non-smokers and ex-smokers were more in favour of smoke-free policies than smokers.

Strengths of the study include the comprehensive search in six databases, and the absence of any language restriction. With 107 reports included covering data from 33 different countries, the body of existing literature describing support was substantial. Data from 99 studies were pooled for 15 different areas of smoke-free policies, and we accounted for potential clustering at country-level by applying a three-level meta-analysis. Hence, we accounted for variance at participant, study, and country level. In addition to the meta-analysis we conducted subgroup analyses, meta-regression, and sensitivity analyses, providing additional insight in the patterning of public support for novel smoke-free policies. Sensitivity analyses generally showed no meaningful differences in support between lower and higher risk-of-bias studies, suggesting that the evidence is robust.

A limitation of this study is that generalisability to other countries from which no surveys were available may be limited. Although there was no indication for publication bias, countries included might be subject to bias, as it is likely that countries planning for novel ways to reduce negative harms of tobacco among the population are more likely to survey support for such policies. This is reflected by the large number of studies from the USA where traditional smoke-free policies are already commonplace, and the low proportion of studies from LMICs, where governmental actions regarding tobacco are commonly more limited. Thirteen reports with 69 estimates in our systematic review were from 11 LMICs. The majority of estimates included in the meta-analysis (33%) however, were from one country (i.e., republic of Georgia). Other limitations of this study concern the different ways in which support was assessed. We did take into consideration the type of answer categories, and did not find large differences when participants were asked if they supported smoke-free policies with a yes-no question or on a Likert scale. Some papers reported proportion not in favour of a policy and outcome estimates were reversed. Although for one study this led to the reported outcome including neutral and positive answers, potentially overestimating support, any bias resulting from this will be very limited. Negative or positive framing of a question might also influence the response.^132^ These aspects may have contributed to the substantial heterogeneity observed across studies, in addition to existing cultural and contextual differences. Finally, the minimum sample size requirement of 400 participants might have caused an underrepresentation of locations with smaller populations, such as subsidised housing or inpatient facilities. For a complete overview of excluded full reports see Appendix XI.

Previous studies have shown strong increases in support for novel smoke-free policies over time,^11^ which was not confirmed in our study. Since support was generally high, it is possible that support already reached a plateau, after which little change is observed. Policy makers might be concerned that actual implementation can backfire support. Our review indicates that this concern is not backed by previous literature, with 6 out of 12 studies that assessed support before and after implementation showing an increase in support.^31^^,^^45^^,^^59^^,^^76^^,^^122^^,^^127^ Among the remaining studies support was often already high, indicating a plateau in support.^52^^,^^70^^,^^76^^,^^114^ An additional worry could be that support is theoretical and might change when it regards actual implementation. However, our study showed no differences in support between implemented policies and hypothetical scenarios. For outdoor non-hospitality places, the group with most estimates, no significant differences were found, indicating support is equally high for hypothetical questions as for implemented policies. Another consideration for policy makers could be that a high level of support may not necessarily ensure adequate compliance with smoke-free policies, especially in places where enforcement is challenging. Additional research is needed to investigate optimal approaches to enforcement, including self-enforcement in places where formal regulation is lacking.

Public support is an essential element facilitating policy implementation. We identified highest levels of support for places where children are frequently exposed to tobacco smoke. For example, support for smoke-free cars when driving with children was much higher (86%) compared to a generic policy making cars smoke-free (57%). Similarly, support for smoke-free playgrounds (80%) was higher compared to support for smoke-free parks and beaches (53%). On average, support was highest for indoor private places; this was primarily driven by the high level of support for smoke-free cars carrying children. Apart from that, support was generally higher for semi-private places compared to private places. For example, support for smoke-free regulations in multi-unit housing (76%) was higher compared to private housing (59%).

Of all places evaluated in our study, the highest level of support was found for smoke-free cars: eight out of ten participants were in favour of cleaning the air in cars when children are present. This is in agreement with a previous brief review of support for smoke-free cars, which included studies up to 2008.^133^ Our report, including studies up to 2022, similarly shows consistently high levels of support for smoke-free cars with children. This is important given the established impact of smoke-free car policies, which have been shown to reduce exposure to second-hand smoke by 31%,^7^^,^^134^ and reduced paediatric hospital admissions for asthma in Scotland.^135^

A similarity between the places with highest support was their link with children, suggesting the effectiveness of approaching tobacco control as a child health issue.^136^ In addition to protecting children from tobacco smoke exposure, smoke-free policies in these places also protect children through role-modelling, i.e., being less exposed to smoking imagery which decreases their risk of becoming a smoker.^137^^,^^138^ This perspective is especially important to justify smoke-free policies in outdoor areas that are well ventilated but have many underage visitors, such as beaches. Another argument for banning smoking in such areas is its impact on reducing littering from cigarette butts, which contain microplastics and other toxic substances that pollute the environment.^139^^,^^140^ Such aspects may be used to inform the public of why smoke-free policies are important, other than directly protecting individuals from being exposed to other people’s tobacco smoke.

The large number of reports included enabled us to evaluate differences in support between population subgroups. Perhaps unsurprisingly, higher levels of support were found among non-smokers and former smokers compared to current smokers.^11^^,^^141^ Furthermore, subgroup analyses identified a small difference in support among men and women, with higher levels of support among women. This may be because women generally perceive SHS as more harmful compared to men,^142^ and see smoking as less socially acceptable.^143^ As voluntary smoke-free rules are already often applied by parents in places such as private homes and cars,^144^, ^145^, ^146^ we anticipated that levels of support would be higher among parents. However, no significant differences were found between parents and non-parents. This pattern more widely applied to smoke-free places involving children, which may indicate that child health is not solely a matter of parents, but regarded important by society at large.

The results presented in this paper indicate that the majority of the surveyed population is in favour of smoke-free environments beyond places currently being legislated. The consistent high level of support for smoke-free policies in cars carrying children in particular, indicates momentum for increased implementation of regulation in this area. Support was consistently high not only for cars carrying children but for all places where children often go. Framing smoke-free policies as a children’s rights or child health issue as part of a broader smoke-free or tobacco-free generation initiative can provide a good starting point for implementing novel smoke-free policies.^147^^,^^148^ Furthermore, smoke-free policies that go beyond enclosed public places and the workplace should be part of a comprehensive tobacco control programme, including other measures such as tax increases, reducing the number points of sale and banning tobacco display. How support for one measure affects other measures could be assessed in future research.

Support for novel smoke-free policies is generally high across countries, especially for policies in places where children are present. This indicates that there is substantial momentum for policy-makers to take the next step in protecting the public, and children in particular, from the harmful effects of tobacco smoke exposure by expanding smoke-free policies.

## Contributors

JVB secured funding for this work. NWB, FJMM, AzS, AB, FJvL and JVB designed the study and wrote the protocol paper. NWB, AsS and EL contributed to the search, study selection, data extraction and risk of bias assessment. NWB conducted the data analyses and created the figures. JVB supervised the review process. NWB, FJMM and JVB verified the underlying data. NWB, FJMM and JVB drafted the manuscript, AzS, AB and FJvL provided feedback. All authors interpreted the analyses, read and approved the final manuscript, had full access to all the data in the study and had final responsibility for the decision to submit for publication.

## Data sharing statement

All datasets generated and analyses are available in the article and the appendix.

## Declaration of interests

NWB was funded by the Smarter Choices for Better Health Programme from Erasmus University. JVB is PI of a research project to tailor a National Smoking Cessation Programme for (future) parents funded by the Erasmus Initiatives Smarter Choices for Better Health Programme and the Dutch Ministry of Health, Welfare and Sports payed to Erasmus MC. JVB is member of the National Taskforce Smokefree Start, a collaboration of professional organisations involved in the care of pregnant couples, parents, and children aimed at supporting a smoke-free start. This Taskforce is supported by the Trimbos Institute and the Dutch Ministry of Health, Welfare and Sports. Fees for participation in the Taskforce are paid to Erasmus MC. JVB chairs the Taskforce Smokefree Erasmus MC which aims to support a smoke-free hospital environment and optimise smoking cessation support for patients and employees. JVB is a committee member for the national multidisciplinary guideline on tobacco addiction treatment and smoking cessation support.

## References

[1] G.B.D. Risk Factors Collaborators (2020). Global burden of 87 risk factors in 204 countries and territories, 1990-2019: a systematic analysis for the Global Burden of Disease Study 2019. Lancet.

[2] Faber T., Kumar A., Mackenbach J.P. (2017). Effect of tobacco control policies on perinatal and child health: a systematic review and meta-analysis. Lancet Public Health.

[3] Frazer K., Callinan J.E., McHugh J. (2016). Legislative smoking bans for reducing harms from secondhand smoke exposure, smoking prevalence and tobacco consumption. Cochrane Database Syst Rev.

[4] Johns M., Coady M.H., Chan C.A., Farley S.M., Kansagra S.M. (2013). Evaluating New York City's smoke-free parks and beaches law: a critical multiplist approach to assessing behavioral impact. Am J Community Psychol.

[5] Levy D.E., Adams I.F., Adamkiewicz G. (2017). Delivering on the promise of smoke-free public housing. Am J Public Health.

[6] Faber T., Mizani M.A., Sheikh A., Mackenbach J.P., Reiss I.K., Been J.V. (2019). Investigating the effect of England's smoke-free private vehicle regulation on changes in tobacco smoke exposure and respiratory disease in children: a quasi-experimental study. Lancet Public Health.

[7] Rado M.K., Molenberg F.J.M., Westenberg L.E.H. (2021). Effect of smoke-free policies in outdoor areas and private places on children's tobacco smoke exposure and respiratory health: a systematic review and meta-analysis. Lancet Public Health.

[8] Laverty A.A., Filippidis F.T., Been J.V., Campbell F., Cheeseman H., Hopkinson N.S. (2021). Smoke-free vehicles: impact of legislation on child smoke exposure across three countries. Eur Respir J.

[9] IARC Handbooks of Cancer Prevention (2009).

[10] World Health Organization (2009).

[11] Thomson G., Wilson N., Collins D., Edwards R. (2016). Attitudes to smoke-free outdoor regulations in the USA and Canada: a review of 89 surveys. Tob Control.

[12] Boderie N.W., Mölenberg F.J., Sheikh A. (2021). Assessing public support for extending smoke-free policies beyond enclosed public places and workplaces: protocol for a systematic review and meta-analysis. BMJ Open.

[13] Bramer W.M. (2018). Reference checking for systematic reviews using Endnote. J Med Libr Assoc.

[14] World Bank (2021). https://datahelpdesk.worldbank.org/knowledgebase/articles/378832-what-is-the-world-bank-atlas-method.

[15] World Health Organization (2017).

[16] Hong Q.N., Fàbregues S., Bartlett G. (2018). The Mixed Methods Appraisal Tool (MMAT) version 2018 for information professionals and researchers. Educ Inf.

[17] Borenstein M., Hedges L.V., Higgins J.P., Rothstein H.R. (2010). A basic introduction to fixed-effect and random-effects models for meta-analysis. Res Synth Methods.

[18] Van den Noortgate W., Lopez-Lopez J.A., Marin-Martinez F., Sanchez-Meca J. (2013). Three-level meta-analysis of dependent effect sizes. Behav Res Methods.

[19] Viechtbauer W. (2010). Conducting meta-analyses in R with the metafor package. J Stat Softw.

[20] Barker T.H., Migliavaca C.B., Stein C. (2021). Conducting proportional meta-analysis in different types of systematic reviews: a guide for synthesisers of evidence. BMC Med Res Methodol.

[21] Hunter J.P., Saratzis A., Sutton A.J., Boucher R.H., Sayers R.D., Bown M.J. (2014). In meta-analyses of proportion studies, funnel plots were found to be an inaccurate method of assessing publication bias. J Clin Epidemiol.

[22] Abundis F. (2008). The General Tobacco Control Law and public opinion. Salud Publica Mex.

[23] Agaku I.T., Odukoya O.O., Olufajo O., Filippidis F.T., Vardavas C.I. (2014). Support for smoke-free cars when children are present: a secondary analysis of 164,819 U.S. adults in 2010/2011. Eur J Pediatr.

[24] Al-Delaimy W., White M., Gilmer T., Zhu S., Pierce J.P. (2008).

[25] Almutairi K.M. (2014). Attitudes of students and employees towards the implementation of a totally smoke free university campus policy at King Saud University in Saudi Arabia: a cross sectional baseline study on smoking behavior following the implementation of policy. J Community Health.

[26] Atiba Y.M., Olubodun T., Odukoya O.O. (2020). Young peoples' support for a smoke-free campus policy: a case for smoke-free campuses in the statewide smoking law in Lagos State, Nigeria. Ann Afr Med.

[27] Bartington S.E., Wootton R., Hawkins P., Farley A., Jones L.L., Haroon S. (2020). Smoking behaviours and attitudes towards campus-wide tobacco control policies among staff and students: a cross-sectional survey at the University of Birmingham. BMC Public Health.

[28] Basto-Abreu A.C., Christine P.J., Zepeda-Tello R. (2016). Behaviours and opinions towards outdoor smoking bans and cigarette littering in Baja California, Mexico. Health Policy Plan.

[29] Berg C.J., Thrasher J.F., O'Connor J., Haardorfer R., Kegler M.C. (2015). Reactions to smoke-free policies and messaging strategies in support and opposition: a comparison of Southerners and Non-Southerners in the US. Health Behav Policy Rev.

[30] Berg C.J., Topuridze M., Maglakelidze N., Starua L., Shishniashvili M., Kegler M.C. (2016). Reactions to smoke-free public policies and smoke-free home policies in the Republic of Georgia: results from a 2014 national survey. Int J Public Health.

[31] Berg M.B., Lin L. (2022). How effective are campus-wide smoking bans? A comparison of two small colleges. J Am Coll Health.

[32] Bo-Woo L.E.E., Ji-Hyun P., Hyun-Joo K.I.M., Moo-Sik L.E.E., Jin-Yong L.E.E. (2012). Opinions on the recent no-smoking policy in Daejeon Metropolitan City: a focus on the differences of opinions between smokers and non-smokers. Korean J Health Promot.

[33] Boeckmann M., Kotz D., Shahab L., Brown J., Kastaun S. (2018). German public support for tobacco control policy measures: results from the German study on tobacco use (DEBRA), a representative national survey. Int J Environ Res Public Health.

[34] Bower G.G., Enzler D. (2005). Protecting students and faculty from environmental tobacco smoke: an assessment and rationale for college policies prohibiting smoking in public areas and student residencies. Health Educ.

[35] Braverman M.T., Ceraso M., Sporrer F., Rockler B.E. (2021). Five-year changes in support for tobacco control policy options among students, faculty and staff at a public university. Prev Med.

[36] Braverman M.T., Hoogesteger L.A., Johnson J.A. (2015). Predictors of support among students, faculty and staff for a smoke-free university campus. Prev Med.

[37] Brown A., Sweeting H., Logan G., Demou E., Hunt K. (2019). Prison staff and prisoner views on a prison smoking ban: evidence from the tobacco in prisons study. Nicotine Tob Res.

[38] Burns S., Jancey J., Bowser N. (2013). Moving forward: a cross sectional baseline study of staff and student attitudes towards a totally smoke free university campus. BMC Public Health.

[39] Buth S., Stover H., Ritter C. (2013). Tobacco prevention in prisons a survey among prisoners on tobacco use and the possibilities and obstacles regarding the reduction of smoking in prison. Suchttherapie.

[40] Cartanya-Hueso A., Lidon-Moyano C., Fu M. (2019). Apoyo a la regulacion de fumar en el interior de vehiculos privados y espacios publicos al aire libre. Rev Esp Salud Publica.

[41] Chaaya M., Farran D., Saab D. (2021). Influence of a university tobacco-free policy on the attitudes, perceptions of compliance, and policy benefit among the university students: a pre-post investigation. Int J Public Health.

[42] Clegg H., Howle F., Groom K. (2021). Understanding the enablers and barriers to implementing smoke-free NHS sites across acute care trusts in Greater Manchester: results of a hospital staff survey. Future Healthc J.

[43] Crosby S., Bell D., Savva G., Edlin B., Bewick B.M. (2018). The impact of a social norms approach on reducing levels of misperceptions around smokefree hospital entrances amongst patients, staff, and visitors of a NHS hospital: a repeated cross-sectional survey study. BMC Public Health.

[44] Diez-Izquierdo A., Lidon-Moyano C., Martin-Sanchez J.C. (2017). Smoke-free homes and attitudes towards banning smoking in vehicles carrying children in Spain (2016). Environ Res.

[45] Dono J., Bowden J., Ettridge K., Roder D., Miller C. (2014). Monitoring approval of new legislation banning smoking in children’s playgrounds and public transport stops in South Australia. Asia Pac J Clin Oncol.

[46] Drach L.L., Pizacani B.A., Rohde K.L., Schubert S. (2010). The acceptability of comprehensive smoke-free policies to low-income tenants in subsidized housing. Prev Chronic Dis.

[47] Dunn J., Greenbank S., McDowell M. (2008). Community knowledge, attitudes and behaviours about environmental tobacco smoke in homes and cars. Health Promot J Aust.

[48] El Ansari W., Labeeb S., Kotb S., Yousafzai M.T., El-Houfey A., Stock C. (2012). Correlates of smoking, quit attempts and attitudes towards total smoking bans at university: findings from eleven faculties in Egypt. Asian Pac J Cancer Prev.

[49] El Ansari W., Salam A. (2021). Prevalence and predictors of smoking, quit attempts and total smoking ban at the University of Turku, Finland. Cent Eur J Public Health.

[50] El Ansari W., Stock C. (2012). Factors associated with smoking, quit attempts and attitudes towards total smoking bans at university: a survey of seven universities in England, Wales and Northern Ireland. Asian Pac J Cancer Prev.

[51] Fallin A., Roditis M., Glantz S.A. (2015). Association of campus tobacco policies with secondhand smoke exposure, intention to smoke on campus, and attitudes about outdoor smoking restrictions. Am J Public Health.

[52] Farran D., Nakkash R., Al-Hindi M. (2021). Evaluating a tobacco-free university policy: a repeated cross-sectional survey of faculty and staff in Lebanon. Tob Induc Dis.

[53] Fong G.T., Craig L.V., Guignard R. (2013). Evaluation of the smoking ban in public places in France one year and five years after its implementation: findings from the ITC France survey. Bull Epidemiol Hebd.

[54] Gallus S., Rosato V., Zuccaro P. (2012). Attitudes towards the extension of smoking restrictions to selected outdoor areas in Italy. Tob Control.

[55] Garg T., Fradkin N., Moskowitz J.M. (2011). Adoption of an outdoor residential hall smoking policy in a California public university: a case study. J Am Coll Health.

[56] Gendall P., Hoek J., Maubach N., Edwards R. (2013). Public support for more action on smoking. N Z Med J.

[57] Gentzke A.S., Hyland A., Kiviniemi M., Travers M.J. (2018). Attitudes and experiences with secondhand smoke and smoke-free policies among subsidised and market-rate multiunit housing residents living in six diverse communities in the USA. Tob Control.

[58] Gillespie J., Milne K., Wilson N. (2005). Secondhand smoke in New Zealand homes and cars: exposure, attitudes, and behaviours in 2004. N Z Med J.

[59] Hale N., Murphy A.M., Adams J.R., Williams C.M. (2017). Effect of a smoke-free policy on staff attitudes and behaviours within an Australian metropolitan health service: a 3 year cross-sectional study. Aust Health Rev.

[60] Hammond D., Costello M.J., Fong G.T., Topham J. (2006). Exposure to tobacco marketing and support for tobacco control policies. Am J Health Behav.

[61] Hewett M.J., Ortland W.H., Brock B.E., Heim C.J. (2012). Secondhand smoke and smokefree policies in owner-occupied multi-unit housing. Am J Prev Med.

[62] Ickes M.J., Rayens M.K., Wiggins A., Hahn E.J. (2017). Students' beliefs about and perceived effectiveness of a tobacco-free campus policy. Policy Polit Nurs Pract.

[63] Jalleh G., Donovan R.J., Stewart S., Sullivan D. (2006). Is there public support for banning smoking in motor vehicles?. Tob Control.

[64] Johnston Polacek G.N.L., Atkins J.L. (2008). Smoking behavior, attitudes of second-hand smoke, and no-smoking policies on a university campus. Health Educ.

[65] Kandra K.L., Goldstein A.O., Gizlice Z., Woldman R.L., Proescholdbell S.K. (2007). Attitudes about tobacco policies among North Carolina parents. N C Med J.

[66] Kandra K.L., McCullough A., Ranney L., Goldstein A.O. (2013). Support among middle school and high school students for smoke-free policies, North Carolina, 2009. Prev Chronic Dis.

[67] Karadag M., Aydin Guclu O., Gorek Dilektasli A., Coskun F., Uzaslan E. (2021). Universite ogrencilerinin sigara kullanma durumlari ve tutunsuz kampus politikasina yonelik tutumlarinin degerlendirilmesi. Tuberk Toraks.

[68] Kecojevic A., Kernan W.D., Urena A., Pereda A., Shair R., Amaya-Fernandez E. (2020). Support for 100% tobacco-free policy on a college campus in New Jersey: differences between students and faculty/staff. J Public Health.

[69] Kennedy R.D., Behm I., Craig L. (2012). Outdoor smoking behaviour and support for outdoor smoking restrictions before and after France's national smoking ban. Eur J Public Health.

[70] Kennedy R.D., Ellens-Clark S., Nagge L., Douglas O., Madill C., Kaufman P. (2015). A smoke-free community housing policy: changes in reported smoking behaviour-findings from Waterloo Region, Canada. J Community Health.

[71] King B.A., Cummings K.M., Mahoney M.C., Juster H.R., Hyland A.J. (2010). Multiunit housing residents' experiences and attitudes toward smoke-free policies. Nicotine Tob Res.

[72] King B.A., Homa D.M., Dube S.R., Babb S.D. (2014). Exposure to secondhand smoke and attitudes toward smoke-free workplaces among employed U.S. adults: findings from the National Adult Tobacco Survey. Nicotine Tob Res.

[73] Klein E.G., Forster J.L., McFadden B., Outley C.W. (2007). Minnesota tobacco-free park policies: attitudes of the general public and park officials. Nicotine Tob Res.

[74] Kruger J., Jama A., Kegler M., Marynak K., King B. (2016). National and state-specific attitudes toward smoke-free parks among U.S. adults. Int J Environ Res Public Health.

[75] Kruger J., Patel R., Kegler M.C., Brener N.D., King B.A. (2015). National and state attitudes of US adults toward tobacco-free school grounds, 2009-2010. Prev Chronic Dis.

[76] Lechner W.V., Meier E., Miller M.B., Wiener J.L., Fils-Aime Y. (2012). Changes in smoking prevalence, attitudes, and beliefs over 4 years following a campus-wide anti-tobacco intervention. J Am Coll Health.

[77] Li J., Newcombe R. (2013).

[78] Li J., Newcombe R., Walton D. (2016). Responses towards additional tobacco control measures: data from a population-based survey of New Zealand adults. N Z Med J.

[79] Licht A.S., King B.A., Travers M.J., Rivard C., Hyland A.J. (2012). Attitudes, experiences, and acceptance of smoke-free policies among US multiunit housing residents. Am J Public Health.

[80] Lin D., Stahl D.C., Ikle D., Grannis F.W. (2006). Employee attitudes and smoking behavior at the City of Hope National Medical Center smoke-free campus. J Natl Compr Canc Netw.

[81] Loukas A., Garcia M.R., Gottlieb N.H. (2006). Texas college students' opinions of no-smoking policies, secondhand smoke, and smoking in public places. J Am Coll Health.

[82] Lugo A., Zuccaro P., Pacifici R. (2017). Smoking in Italy in 2015-2016: prevalence, trends, roll-your-own cigarettes, and attitudes towards incoming regulations. Tumori.

[83] Lund M. (2016). Exploring smokers' opposition to proposed tobacco control strategies. Nord Stud Alcohol Drugs.

[84] Mamudu H.M., Veeranki S.P., Kioko D.M., Boghozian R.K., Littleton M.A. (2016). Exploring support for 100% college tobacco-free policies and tobacco-free campuses among college tobacco users. J Public Health Manag Pract.

[85] Marsh L., Robertson L.A., Cameron C. (2014). Attitudes towards smokefree campus policies in New Zealand. N Z Med J.

[86] Martinez-Sanchez J.M., Gallus S., Lugo A. (2014). Smoking while driving and public support for car smoking bans in Italy. Tob Control.

[87] McMillen R., Breen J., Cosby A.G. (2004). Rural-urban differences in the social climate surrounding environmental tobacco smoke: a report from the 2002 Social Climate Survey of Tobacco Control. J Rural Health.

[88] McMillen R.C., Winickoff J.P., Gottlieb M.A., Tanski S., Wilson K., Klein J.D. (2019). Public support for smoke-free section 8 public housing. West J Nurs Res.

[89] Meng Y.Y., Rahman T., Hanaya D. (2016). Unequal protection: secondhand smoke threatens health of tenants in multi-unit housing in Los Angeles. Policy Brief UCLA Cent Health Policy Res.

[90] Mishra S., Thind H.K., Gokarakonda S.B., Lartey G., Watkins C., Chahal M. (2011). Second-hand smoke in a university campus: attitudes and perceptions of faculty, staff and students. Int J Health Res.

[91] Morain S., Mello M.M. (2013). Survey finds public support for legal interventions directed at health behavior to fight noncommunicable disease. Health Aff.

[92] Niemeier B.S., Chapp C.B., Henley W.B. (2014). Improving tobacco-free advocacy on college campuses: a novel strategy to aid in the understanding of student perceptions about policy proposals. J Am Coll Health.

[93] Nogueira S.O., Fu M., Lugo A. (2022). Non-smokers' and smokers' support for smoke-free legislation in 14 indoor and outdoor settings across 12 European countries. Environ Res.

[94] Okoli C.T., Pederson A., Rice W. (2013). Support for a smoke-free bylaw in parks and on beaches. Health Policy.

[95] Patel M., Donovan E.M., Liu M., Solomon-Maynard M., Schillo B.S. (2022). Policy support for smoke-free and E-cigarette free multiunit housing. Am J Health Promot.

[96] Pederson A., Okoli C.T., Hemsing N. (2016). Smoking on the margins: a comprehensive analysis of a municipal outdoor smoke-free policy. BMC Public Health.

[97] Ramachandran S., Bentley S., Casey E., Bentley J.P. (2020). Prevalence of and factors associated with violations of a campus smoke-free policy: a cross-sectional survey of undergraduate students on a university campus in the USA. BMJ Open.

[98] Rashid A., Manan A.A., Yahya N., Ibrahim L. (2014). The support for smoke free policy and how it is influenced by tolerance to smoking - experience of a developing country. PLoS One.

[99] Reuband K.H. (2014). Tabakkonsum im gesellschaftlichen Wandel. Verbreitung des Konsums und Einstellung zu Rauchverboten, Dusseldorf 1997-2009. Gesundheitswesen.

[100] Rhoades R.R., Beebe L.A., Mushtaq N. (2019). Support for local tobacco policy in a preemptive state. Int J Environ Res Public Health.

[101] Riad-Allen L., Dermody S.S., Herman Y., Bellissimo K., Selby P., George T.P. (2017). Becoming tobacco-free: changes in staff and patient attitudes and incident reports in a large academic mental health and addictions hospital. Am J Addict.

[102] Rodríguez-González A.M., Salinas-Martínez A.M., Elizondo-Omaña R.E. (2022). Opinion of the law of protection against exposure to tobacco smoke in adults. J Subst Use.

[103] Rosen L.J., Rier D.A., Schwartz R. (2012). Public support for smoke-free areas in Israel: a case for action. Health Policy.

[104] Rosenberg M., Pettigrew S., Wood L., Ferguson R., Houghton S. (2012). Public support for tobacco control policy extensions in Western Australia: a cross-sectional study. BMJ Open.

[105] Ruokolainen O., Ollila H., Patja K., Borodulin K., Laatikainen T., Korhonen T. (2018). Social climate on tobacco control in an advanced tobacco control country: a population-based study in Finland. Nordisk Alkohol Nark.

[106] Sabrian F., Utomo W. (2019). Perceptions of students, lecturers and staffs on establishing a smoke-free campus. Enferm Clin.

[107] Saebo G., Lund P.B. (2019). Children's right to smoke-free air: public support in Norway for banning smoking in vehicles with children present. Health Policy.

[108] Schmidt L.M., Reidmohr A.A., Helgerson S.D., Harwell T.S. (2016). Secondhand smoke exposure and smoke-free policy support among public housing authority residents in rural and tribal settings. J Community Health.

[109] Sendall M.C., Fox L., Wraith D. (2021). University staff and students' attitudes towards a completely smoke-free campus: shifting social norms and organisational culture for health promotion. Int J Environ Res Public Health.

[110] Seo D.C. (2005). Correlates of attitudes toward a smoking ban in vehicles. J Public Health Manag Pract.

[111] Stevenson A.M., Bradshaw R., Cook J. (2008). Majority of smokers and non-smokers in favour of smokefree parks in New Zealand. N Z Med J.

[112] Stillman F.A., Tanenbaum E., Wewers M.E. (2018). Variations in support for secondhand smoke restrictions across diverse rural regions of the United States. Prev Med.

[113] Sureda X., Fernandez E., Martinez-Sanchez J.M. (2015). Secondhand smoke in outdoor settings: smokers' consumption, non-smokers' perceptions, and attitudes towards smoke-free legislation in Spain. BMJ Open.

[114] Sweeting H., Demou E., Brown A., Hunt K. (2021). Prisoners and prison staff express increased support for prison smoking bans following implementation across Scotland: results from the tobacco in prisons study. Tob Control.

[115] Thompson B., Coronado G.D., Chen L., Thompson L.A., Hymer J.C., Peterson A.K. (2006). Preferred smoking policies at 30 Pacific Northwest colleges. Public Health Rep.

[116] Topuridze M., Berg C.J., Dekanosidze A. (2020). Smokers' and nonsmokers' receptivity to smoke-free policies and pro- and anti-policy messaging in Armenia and Georgia. Int J Environ Res Public Health.

[117] Unrod M., Oliver J.A., Heckman B.W., Simmons V.N., Brandon T.H. (2012). Outdoor smoking ban at a cancer center: attitudes and smoking behavior among employees and patients. J Public Health Manag Pract.

[118] Waddell E.N., Farley S.M., Mandel-Ricci J., Kansagra S.M. (2014). Public support for smoke-free air strategies among smokers and nonsmokers, New York City, 2010-2012. Prev Chronic Dis.

[119] Wallar L.E., Croteau S., Estill A., Robson L., Papadopoulos A. (2013). Analyzing exposure, use, and policies related to tobacco use on campus for the development of comprehensive tobacco policies at Canadian post-secondary institutions. J Community Health.

[120] Walsh R.A., Paul C.L., Tzelepis F., Stojanovski E., Tang A. (2008). Is government action out-of-step with public opinion on tobacco control? Results of a New South Wales population survey. Aust N Z J Public Health.

[121] Wang T.W., Lemos P.R., McNabb S., King B.A. (2018). Attitudes toward smoke-free public housing among U.S. adults, 2016. Am J Prev Med.

[122] Wheeler J.G., Pulley L., Felix H.C. (2007). Impact of a smoke-free hospital campus policy on employee and consumer behavior. Public Health Rep.

[123] Wiium N., Aaro L.E., Hetland J. (2009). Psychological reactance and adolescents' attitudes toward tobacco-control measures. J Appl Soc Psychol.

[124] Williams R.D.J., Barnes J.T., Hunt B.P. (2011). Health beliefs related to secondhand smoke and smoke-free policies in a college community. Am J Health Behav.

[125] Wipfli H., Bhuiyan M.R., Qin X. (2020). Tobacco use and E-cigarette regulation: perspectives of university students in the Asia-Pacific. Addict Behav.

[126] Wong S.L., Epperson A.E., Rogers J., Castro R.J., Jackler R.K., Prochaska J.J. (2020). A multimodal assessment of tobacco use on a university campus and support for adopting a comprehensive tobacco-free policy. Prev Med.

[127] Wray R.J., Hansen N., Ding D., Masters J. (2021). Effects of a campus-wide tobacco-free policy on tobacco attitudes, norms and behaviors among students, staff and faculty. J Am Coll Health.

[128] Xiao L., Jiang Y., Zhang J., Parascandola M. (2020). Secondhand smoke exposure among nonsmokers in China. Asian Pac J Cancer Prev.

[129] Kennedy R.D., Behm I., Craig L. (2012). Smoking cessation interventions from health care providers before and after the national smoke-free law in France. Eur J Public Health.

[130] World Health Organization (2003).

[131] Harrer M., Cuijpers P., Furukawa T.A., Ebert D.D. (2021).

[132] Lee J.G.L., Purcell C.J., Chaney B.H. (2017). An experiment assessing punitive versus wellness framing of a tobacco-free campus policy on students' perceived level of university support. Int J Environ Res Public Health.

[133] Thomson G., Wilson N. (2009). Public attitudes to laws for smoke-free private vehicles: a brief review. Tob Control.

[134] Laverty A.A., Hone T., Vamos E.P. (2020). Impact of banning smoking in cars with children on exposure to second-hand smoke: a natural experiment in England and Scotland. Thorax.

[135] Mackay D.F., Turner S.W., Semple S.E., Dick S., Pell J.P. (2021). Associations between smoke-free vehicle legislation and childhood admissions to hospital for asthma in Scotland: an interrupted time-series analysis of whole-population data. Lancet Public Health.

[136] Willemsen M.C., Been J.V. (2022). Accelerating tobacco control at the national level with the smoke-free generation movement in the Netherlands. NPJ Prim Care Respir Med.

[137] Song A.V., Dutra L.M., Neilands T.B., Glantz S.A. (2015). Association of smoke-free laws with lower percentages of new and current smokers among adolescents and young adults: an 11-year longitudinal study. JAMA Pediatr.

[138] Bommelé J., van Laar M., Kleinjan M. (2016).

[139] van Schalkwyk M.C.I., Novotny T.E., McKee M. (2019). No more butts. BMJ.

[140] Araújo M.C.B., Costa M.F. (2019). A critical review of the issue of cigarette butt pollution in coastal environments. Environ Res.

[141] Lupton J.R., Townsend J.L. (2015). A systematic review and meta-analysis of the acceptability and effectiveness of university smoke-free policies. J Am Coll Health.

[142] Kruger J., Patel R., Kegler M., Babb S.D., King B.A. (2016). Perceptions of harm from secondhand smoke exposure among U.S. adults, 2009-2010. Tob Induc Dis.

[143] Grard A., Schreuders M., Alves J. (2019). Smoking beliefs across genders, a comparative analysis of seven European countries. BMC Public Health.

[144] Murphy-Hoefer R., Madden P., Maines D., Coles C. (2014). Prevalence of smoke-free car and home rules in Maine before and after passage of a smoke-free vehicle law, 2007-2010. Prev Chronic Dis.

[145] Shojima K., Tabuchi T. (2019). Voluntary home and car smoke-free rules in Japan: a cross-sectional study in 2015. BMJ Open.

[146] Jankowski M., Pinkas J., Zgliczynski W.S. (2020). Voluntary smoke-free home rules and exposure to secondhand smoke in Poland: a national cross-sectional survey. Int J Environ Res Public Health.

[147] Hadjipanayis A., Stiris T., Del Torso S., Mercier J.C., Valiulis A., Ludvigsson J. (2017). Europe needs to protect children and youths against secondhand smoke. Eur J Pediatr.

[148] Been J.V., Laverty A.A., Tsampi A., Filippidis F.T. (2021). European progress in working towards a tobacco-free generation. Eur J Pediatr.

