# Supplementary material for: Public support for smoke-free policies in outdoor areas and (semi-)private places: a systematic review and meta-analysis
Source: eClinicalMedicine. 2023 May 9;59:101982. doi: 10.1016/j.eclinm.2023.101982 (PMC10225670; doi:10.1016/j.eclinm.2023.101982)
Supplement: Appendix [file mmc1.pdf]

Appendix I – Search strategy

embase.com (1974-)

('smoking regulation'/exp OR (smokefree OR ((smoking OR smoke OR tobacco) NEAR/3 (regulation* OR government* OR law OR laws OR policy OR policies OR ban OR bans OR banned OR free OR restrict* OR act OR acts))):ab,ti) AND ('public opinion'/exp OR 'public attitude'/de OR (opinion* OR support* OR views OR (public NEAR/3 view) OR attitude* OR feeling* OR acceptance* OR accepts OR perception* OR misperception*):ab,ti) NOT ([Conference Abstract]/lim OR [Letter]/lim OR [Note]/lim OR [Conference Paper]/lim OR [Editorial]/lim)

Medline Ovid (1946-)

(Smoke-Free Policy/ OR (smokefree OR ((smoking OR smoke OR tobacco) ADJ3 (regulation* OR government* OR law OR laws OR policy OR policies OR ban OR bans OR banned OR free OR restrict* OR act OR acts))).ab,ti.) AND (Public Opinion/ OR Attitude/ OR (opinion* OR support* OR views OR (public ADJ3 view) OR attitude* OR feeling* OR acceptance* OR accepts OR perception* OR misperception*).ab,ti.) NOT (news OR congres* OR abstract* OR book* OR chapter* OR dissertation abstract*).pt.

Web of science Core Collection (1900-)

TS=(((smokefree OR ((smoking OR smoke OR tobacco) NEAR/2 (regulation* OR government* OR law OR laws OR policy OR policies OR ban OR bans OR banned OR free OR restrict* OR act OR acts)))) AND ((opinion* OR support* OR views OR (public NEAR/2 view) OR attitude* OR feeling* OR acceptance* OR accepts OR perception* OR misperception*))) AND DT=(article)

Cochrane Central register of trials (1992-issue 9, September 2019)

((smokefree OR ((smoking OR smoke OR tobacco) NEAR/3 (regulation* OR government* OR law OR laws OR policy OR policies OR ban OR bans OR banned OR free OR restrict* OR act OR acts))):ab,ti) AND ((opinion* OR support* OR views OR (public NEAR/3 view) OR attitude* OR feeling* OR acceptance* OR accepts OR perception* OR misperception*):ab,ti)

CINAHL EBSCOhost (1937-)

(TI (smokefree OR ((smoking OR smoke OR tobacco) N2 (regulation* OR government* OR law OR laws OR policy OR policies OR ban OR bans OR banned OR free OR restrict* OR act OR acts))) OR AB (smokefree OR ((smoking OR smoke OR tobacco) N2 (regulation* OR government* OR law OR laws OR policy OR policies OR ban OR bans OR banned OR free OR restrict* OR act OR acts)))) AND (MH Public Opinion OR MH Attitude OR TI (opinion* OR support* OR views OR (public N2 view) OR attitude* OR feeling* OR acceptance* OR accepts OR perception* OR misperception*) OR AB (opinion* OR support* OR views OR (public N2 view) OR attitude* OR feeling* OR acceptance* OR

accepts OR perception* OR misperception*)) NOT PT (news OR congres* OR abstract* OR book* OR chapter* OR dissertation abstract*)

PsycINFO Ovid (1806-)

((smokefree OR ((smoking OR smoke OR tobacco) ADJ3 (regulation* OR government* OR law OR laws OR policy OR policies OR ban OR bans OR banned OR free OR restrict* OR act OR acts))).ab,ti.) AND (Public Opinion/ OR Attitudes/ OR (opinion* OR support* OR views OR (public ADJ3 view) OR attitude* OR feeling* OR acceptance* OR accepts OR perception* OR misperception*).ab,ti.) NOT (news OR congres* OR abstract* OR book* OR chapter* OR dissertation abstract*).pt.

Appendix II – Studies among (former) smokers only

Author (year)	Geographic location	Smoke-free location	Support measure	Answer options	Overall sample size	Reported support overall	Support transformed, reversed etc.
Driezen (2020)	USA, Canada, UK	Multi-unit housing	Prefer a policy in their building prohibiting smoking in "all areas, including individual residences, common areas, and exterior grounds."	Strongly prefer, somewhat prefer, slightly prefer, and would not prefer	Living in Multi-unit housing: n = 2168	36.1% of smokers in multi-unit housing	31.6%
Edwards (2021)	New Zealand	Cars with children	Support for a total ban on smoking in cars with children in them	Agree (strongly agree, agree); not agree (oppose, strongly oppose, refused, don't know)	Wave 1: n = 1,155<br>Wave 2: n = 1,020	92% (smokers and recent quitters)	
Fu (2018)	Germany, Greece, Hungary, Poland, Romania and Spain	Cars with non-smokers	At which of the following places do you think smoking SHOULD be allowed?	Support (strongly support, support); oppose (oppose, strongly oppose)	Germany: n = 1003<br>Greece: n = 1000<br>Hungary: n = 1000<br>Poland: n = 1006<br>Romania: n = 1001<br>Spain: n=998	Among smokers:<br>G:88.0% (84.7 - 91.2)<br>GR: 88.6% (85.5 - 91.6)<br>H: 87.3% (83.5 - 91.1)<br>PL: 86.5% (82.6 - 90.4)<br>RO: 95.1% (93.2 - 97.0)<br>E: 77.5% (73.4 - 81.5)	
Fu (2018)	Germany, Greece, Hungary, Poland, Romania and Spain	Cars with children	At which of the following places do you think smoking SHOULD be allowed?	Support (strongly support, support); oppose (oppose, strongly oppose)	Germany: n = 1003<br>Greece: n = 1000<br>Hungary: n = 1000	Among smokers:<br>G: 97.1% (95.6-98.6)<br>GR: 98.5%	

					Poland: n = 1006<br>Romania: n = 1001<br>Spain: n= 998	(97.7-99.4)<br>H: 92.7% (89.7-95.6)<br>PL: 90.9% (88.3-93.4)<br>RO: 97.3% (96.2-98.4)<br>E: 92.5% (89.8-95.1)
Fu (2018)	Germany, Greece, Hungary, Poland, Romania, Spain	Beaches	At which of the following places do you think smoking SHOULD be allowed?	Support (support, strongly support); oppose (oppose, strongly oppose)	Germany: n = 1003<br>Greece: n = 1000<br>Hungary: n = 1000<br>Poland: n = 1006<br>Romania: n = 1001<br>Spain: n=998	Among smokers:<br>G: 27.4% (22.6 - 32.1)<br>GR: 11.1% (8.1 - 14.1)<br>H: 50.6% (44.0 - 57.2)<br>PL: 61.0% (56.2 - 65.9)<br>RO: 38.1% (33.0 - 43.1)<br>E: 15.7% (12.3 - 19.2)
Fu (2018)	Germany, Greece, Hungary, Poland, Romania, Spain	Bus stops	At which of the following places do you think smoking SHOULD be allowed?	Oppose (oppose, strongly oppose); support (support, strongly support)	Germany: n = 1003<br>Greece: n = 1000<br>Hungary: n = 1000<br>Poland: n = 1006<br>Romania: n = 1001<br>Spain: n=998	Among smokers:<br>G: 26.8% (22.5 - 31.2)<br>GR: 25.8% (20.4 - 31.2)<br>H: 64.9% (59.0 - 70.8)<br>PL: 72.1% (67.7 - 76.6)<br>RO: 50.3% (45.4 - 55.2)

						E: 33.0% (28.8 - 37.2)
Fu (2018)	Germany, Greece, Hungary, Poland, Romania, Spain	Subway and train stations	At which of the following places do you think smoking SHOULD be allowed?	Oppose (oppose, strongly oppose); support (support, strongly support)	Germany: n = 1003<br>Greece: n = 1000<br>Hungary: n = 1000<br>Poland: n = 1006<br>Romania: n = 1001<br>Spain: n=998	Among smokers:<br>G: 53.6% (48.9 - 58.3)<br>GR: 67.5% (60.6 - 74.5)<br>H: 69.7% (63.6 - 75.8)<br>PL: 79.5% (75.9 - 83.2)<br>RO: 73.1% (68.4 - 77.8)<br>E: 63.2% (57.7 - 68.8)
Fu (2018)	Germany, Greece, Hungary, Poland, Romania, Spain	In schoolyards of primary schools	At which of the following places do you think smoking SHOULD be allowed?	Oppose (oppose, strongly oppose); support (support, strongly support)	Germany: n = 1003<br>Greece: n = 1000<br>Hungary: n = 1000<br>Poland: n = 1006<br>Romania: n = 1001<br>Spain: n = 998	Among smokers: G: 97.0% (95.7 - 98.2)<br>GR: 94.5% (91.6 - 97.5)<br>H: 93.4% (90.6 - 96.2)<br>PL: 90.2% (87.5 - 92.9)<br>RO: 95.3% (93.0 - 97.7)<br>E: 95.6% (93.1 - 98.1)
Fu (2018)	Germany, Greece, Hungary, Poland, Romania, Spain	In schoolyards of secondary schools	At which of the following places do you think smoking SHOULD be allowed?	Oppose (oppose, strongly oppose); support (support, strongly support)	Germany: n = 1003<br>Greece: n = 1000<br>Hungary: n = 1000<br>Poland: n = 1006<br>Romania: n= 1001<br>Spain: n = 998	Among smokers:<br>G: 88.1% (85.4 - 90.9)<br>GR: 92.9% (89.6 - 96.2)

						H: 92.7% (90.1 - 95.2)<br>PL: 85.7% (82.4 - 89.0)<br>RO: 95.2% (92.8 - 97.6)<br>E: 91.2% (87.5 - 94.8)
Fu (2018)	Germany, Greece, Hungary, Poland, Romania, Spain	Open stadiums for events	At which of the following places do you think smoking SHOULD be allowed?	Oppose (oppose, strongly oppose); support (support, strongly support)	Germany: n = 1003<br>Greece: n = 1000<br>Hungary: n = 1000<br>Poland: n = 1006<br>Romania: n = 1001<br>Spain: n = 998	Among smokers:<br>G: 32.5% (28.2 - 36.9)<br>GR: 24.4% (18.4 - 30.3)<br>H: 65.9% (60.6 - 71.2)<br>PL: 78.4% (74.6 - 82.2)<br>RO: 47.0% (40.9 - 53.1)<br>E: 31.3% (27.0 - 35.6)
Fu (2018)	Germany, Greece, Hungary, Poland, Romania, Spain	Outdoor terraces of bars and pubs	At which of the following places do you think smoking SHOULD be allowed?	Oppose (oppose, strongly oppose); support (support, strongly support)	Germany: n = 1003<br>Greece: n = 1000<br>Hungary: n = 1000<br>Poland: n = 1006<br>Romania: n = 1001<br>Spain: n=998	Among smokers:<br>G: 3.5% (1.6 - 5.3)<br>GR:4.1% (2.2 - 6.0)<br>H: 22.1% (16.8 - 27.3)<br>PL: 15.5% (12.1 - 18.8)<br>RO: 3.5% (2.0 - 5.0)<br>E: 3.4% (2.1 - 4.7)

Fu (2018)	Germany, Greece, Hungary, Poland, Romania, Spain	Outdoor terraces of restaurants	At which of the following places do you think smoking SHOULD be allowed?	Oppose (oppose, strongly oppose); support (support, strongly support)	Germany: n = 1003<br>Greece: n = 1000<br>Hungary: n = 1000<br>Poland: n = 1006<br>Romania: n = 1001<br>Spain: n=998	Among smokers:<br>G: 8.5% (6.2 - 10.8)<br>GR: 5.2% (3.1 - 7.3)<br>H: 24.3% (18.6 - 30.0)<br>PL: 15.3% (12.3 - 18.4)<br>RO: 3.2% (1.7 - 4.7)<br>E: 4.2% (2.1 - 6.2)	
Hitchman (2011)	USA, Canada, UK and Australia	Cars with children	Would you support a law that banned smoking in cars when children are in them?	Yes; no	USA: n = 1427<br>Canada: n = 1622<br>UK: n = 1608<br>Australia: n = 1575	Among smokers:<br>USA: 59%<br>Canada: 74%<br>UK: 75%<br>Australia: 82%	
Park (2019)	South Korea	Smoking ban in parks	For each of the following public places, please tell me if you think smoking should be:	Allowed in all areas; in some areas; not allowed at all	2005: n = 995<br>2008: n = 1737<br>2010: n = 1560	2008: 18.4%<br>2010: 29.8%	2008: 18.4%<br>2010: 29.8%
Park (2019)	South Korea	Smoking ban in bus stops	For each of the following public places, please tell me if you think smoking should be:	Allowed in all areas; in some areas; not allowed at all	2005: n = 995<br>2008: n = 1737<br>2010: n = 1560	2005: NA<br>2008: 29.9%<br>2010: 44.2%	2005: NA<br>2008: 29.9%<br>2010: 44.2%
Park (2019)	South Korea	Smoking ban in crowded streets	For each of the following public places, please tell me if you think smoking should be:	Not allowed at all; in some areas; allowed in all areas	2005: n = 995<br>2008: n = 1737<br>2010: n = 1560	2005: NA<br>2008: 28.0%<br>2010: 36.6%	2005: NA<br>2008: 28.0%<br>2010: 36.6%
Park (2019)	South Korea	Restaurants and cafes outdoor areas	For each of the following public places, please tell me if you think smoking should be:	Not allowed at all; in some areas; allowed in all areas	2005: n = 995<br>2008: n = 1737<br>2010: n = 1560	2005: NA<br>2008: 4.1%<br>2010: 9.7%	2005: NA<br>2008: 4.1%<br>2010: 9.7%

Park (2019)	South Korea	Smoking ban in drinking establishments outdoor areas	For each of the following public places, please tell me if you think smoking should be:	Allowed in all areas; in some areas; not allowed at all	2005: n = 995<br>2008: n = 1737<br>2010: n = 1560	2005: NA<br>2008: 3.4%<br>2010: 4.6%	2005: NA<br>2008: 3.4%<br>2010: 4.6%
Siddiqi (2021)	Pakistan	Cars with minors	Ban on smoking in cars with minors	Support (strongly support, support); neither support nor oppose; oppose (oppose, strongly oppose)	n = 5901	86.5% (among smokers)	
Sohlberg (2019)	Sweden	Playgrounds	Ban against smoking at playgrounds	Support (to a great extent, totally); not support (not at all, to some extent)	n = 663	Among former smokers: 93.9%	
Sohlberg (2019)	Sweden	Platforms, bus stops, taxi zones outdoors	Ban against smoking at platforms, bus stops, taxi zones outdoors	Support (to a great extent, totally); not support (not at all, to some extent)	n = 663	82.5%	
Sohlberg (2019)	Sweden	Entrances where public has access	Ban against smoking at entrances where the public has access	Support (to a great extent, totally); not support (not at all, to some extent)	n = 663	92.2%	
Sohlberg (2019)	Sweden	Outdoor seating areas at restaurants/bars	Ban against smoking at outdoor seating areas at restaurants/bars	Support (to a great extent, totally); not support (not at all, to some extent)	n = 663	80.3%	
Thomson (2009a)	New Zealand	Cars with pre-school children	Do you think smoking should be allowed in cars with pre-school children in them?	Agree (Strongly agree, agree); disagree (neither agree nor disagree, disagree, strongly disagree)	n = 1367	See subgroup gender	95.7%
Thomson (2009b)	New Zealand	Playgrounds	Do you think smoking should be allowed at	Yes; no	n = 1376	Among smokers:	65.7% (62.3 - 69.1)

			council-owned playgrounds?			65.7% (62.3 - 69.1)	
Wilson (2009)	New Zealand	Cars with children	Do you think smoking should be allowed in cars with pre-school children in them?	Yes; no	n = 1376	3% (1.9-4.0)	97.0%
Wilson (2009)	New Zealand	Playgrounds	Do you think smoking should be allowed at council-owned playgrounds?	Yes; no	n = 1376	31.9% (28.6 - 35.2)	68.1%
Wilson (2009)	New Zealand	Beaches	Do you think smoking should be allowed in beaches?	Yes; no	n = 1376	54.8% (51.2 - 58.3)	45.2%
Wilson (2009)	New Zealand	Entrance ways within 5m of public buildings	Do you think smoking should be allowed in entrance ways within 5m of public buildings?	Yes; no	n = 1376	48.2% (44.6 - 51.7)	51.8%
Wilson (2009)	New Zealand	Outdoors at pubs	Do you think smoking should be allowed in some of the outdoor seating areas of pubs?	Yes; no	n = 1376	82.6% (79.8 - 85.3)	17.4%
Wilson (2009)	New Zealand	Outdoor eating areas	Do you think smoking should be allowed in all outdoor eating areas, in some outdoor eating areas, or not allowed in outdoor eating areas at all?	Yes, all; yes, some; no	n = 1376	25.4% (22.4 - 28.3)	25.4% (22.4 - 28.3)

Appendix III – Support per type of location

Author (year)	Geographic location	Smoke-free location	Support measure	Answer options	Overall sample size	Reported support overall	Support transformed, reversed etc.
**Indoor private - Cars**							
Abundis (2008)	Mexico	Car	Do you agree or disagree with the prohibition of smoking in the following places (…)?	Agree; disagree	n = 1200	65%	
Agaku (2014)	USA	Cars	Inside a car, when other people are present, do you think that smoking should be:	Support (always allowed); not support (allowed under some conditions, never allowed).	n = 164,935	73.3%	
Cartanyà-Hueso (2019)	Barcelona (Spain)	Cars	Should smoking be prohibited in cars?	Yes (agree, totally agree); no (neutral, disagree, totally disagree)	n = 736	61.7%	
Díez-Izquierdo (2017)	Spain	Cars	Should smoking be prohibited inside cars, without exception	Totally agree; agree; neither agree nor disagree; disagree; totally disagree	n = 1036	61.6%	
Dunn (2008)	Queensland (Australia)	Cars	Smoke-free laws should be implemented in the car	Agree (now or as soon as possible); disagree (within 5-10 years)	n = 1026	72.2%	
Gendall (2013)	New Zealand	Cars with non-smokers	Indicate support for smoke-free cars with non-smokers	Eleven-point Likert scale	n = 828	67.0%	67.0%
Gendall (2013)	New Zealand	All cars	Indicate support for smoke-free cars	Eleven-point Likert scale	n = 828	45.0%	45.0%
Gendall (2013)	New Zealand	Smoke free cars combined		Eleven-point Likert scale	n = 828	6.7 out of 10	67.0%

Author (year)	Geographic location	Smoke-free location	Support measure	Answer options	Overall sample size	Reported support overall	Support transformed, reversed etc.
Gillespi (2005)	New Zealand	Cars	Smoking should not be allowed in private cars	Not reported	n = 2731	40.2%	
Jalleh (2006)	Western Australia	Vehicles carrying any passenger	Banning smoking in vehicles when there are any passengers in the vehicle	In favour of; against; no opinion either way	n = 405	See subgroup smoking status	51.9%
Jalleh (2006)	Western Australia	Total ban smoking in cars	Total ban on smoking in vehicles	Low (not at all, a little) intermediate (moderately) and high (a lot)	n = 405	See subgroup smoking status	36.3%
Martínez-Sánchez (2014)	Italy	Cars	Attitude towards smoke-free legislation in cars	Support (moderately in favour, strongly in favour); no support (moderately against, strongly against)	n = 6167	79.6%	
Nogueira (2022)	Bulgaria<br>England<br>France<br>Germany<br>Greece<br>Ireland<br>Italy<br>Latvia<br>Poland<br>Portugal<br>Romania<br>Spain	Cars	For each of the following sites, are you:	Strongly in favour, moderately in favour, moderately against or strongly against a total tobacco ban?	Bulgaria: n = 1,050<br>England: n = 1,013<br>France: n = 1,018<br>Germany: n = 1,031<br>Greece: n = 1,000<br>Ireland: n = 941<br>Italy: n = 1,059<br>Latvia: n = 1,022<br>Poland: n = 724<br>Portugal: n = 1,000<br>Romania: n = 1,018<br>Spain: n = 1,026	Strongly in favour: 38.3%<br>Moderately in favour: 15.8%	54.1%
Seo (2005)	Indiana (USA)	Car drivers	Would you favour or oppose an ordinance that prohibits people from	Favour; oppose	n = 529	34%	

Author (year)	Geographic location	Smoke-free location	Support measure	Answer options	Overall sample size	Reported support overall	Support transformed, reversed etc.
Seo (2005)	Indiana (USA)	Cars with passengers	smoking while driving a car?<br>Would you favour or oppose an ordinance that prohibits passengers in cars from smoking?	Favour; oppose	n = 529	70%	
Stillman (2018)	USA	Cars	Attitudes towards smoking in cars	Not allowed (should never be allowed); allowed (only under some conditions; should always be allowed)	Urban:181,162<br>Rural:47,805	Urban:74.1%<br>Rural:69.1%	73.0%
**Indoor private – housing**							
Abundis (2008)	Mexico	Own house	Do you agree or disagree with the prohibition of smoking in the following places (…)?	Agree; disagree	n = 1200	57.0%	
Berg (2015)	Alabama, Florida, Georgia, Kentucky, North Carolina, Mississippi, South Carolina, Tennessee (USA)	Within individual apartment units in a complex	For each of the following places, indicate how you feel about a policy prohibiting smoking in that kind of place	Oppose (strongly oppose, somewhat oppose); other (neutral, somewhat favour, strongly favour	n = 2501	26.3%	73%
Berg (2016)	Republic of Georgia	Within individual apartments	How would you feel about a policy prohibiting smoking in that kind of place?	Oppose (strongly oppose, somewhat oppose); other (neutral, somewhat favour, strongly favour	n = 1163	23.9%	76.1%
Dunn (2008)	Queensland, Australia	Homes	Smoke-free laws should be implemented in the home	Agree (now or as soon as possible);	n = 1026	71.9%	

Author (year)	Geographic location	Smoke-free location	Support measure	Answer options	Overall sample size	Reported support overall	Support transformed, reversed etc.
				Disagree (within 5-10 years)			
Hewett (2012)	USA	Residents' units	What rules do you prefer about smoking in residents' units	Strongly prefer smoking permitted; somewhat prefer permitted; no preference; somewhat prefer not permitted; strongly prefer not permitted	n = 472	Strongly prefer: 28.3% (9.9. 18.0) Somewhat prefer: 13.5% (26.9. 38.3)	41.8%
Gillespi (2005)	New Zealand	Homes	Attitudes towards smoking restrictions inside the home	Support(no smoking anywhere); no support(smoking only in set areas, no restrictions)	n = 2731	No smoking anywhere: 31.0% (29.3-32.7) Smoking only in set areas: 34.0% (32.2-35.8) No restrictions: 29.4% (27.7-31.1)	
Morain (2013)	USA	Private spaces	How much would you support the following policies? Make it illegal to smoke in private spaces	Support (somewhat support, strongly support); oppose (somewhat oppose, strongly oppose)	n = 1817	37.9%	
Topuridze (2020)	Armenia, Republic of Georgia	Individual apartments	To what extent do you support or oppose a complete cigarette smoking ban in the following settings?	1: strongly oppose 2: somewhat oppose 3: don't know 4: somewhat	Armenia: n = 705 Georgia: n = 751	Mean (SD) 3.79 (1.47)	69.7%

Author (year)	Geographic location	Smoke-free location	Support measure	Answer options	Overall sample size	Reported support overall	Support transformed, reversed etc.
				support<br>5: strongly support			
**Indoor private – cars with vulnerable passengers**							
Agaku (2014)	USA	Cars with children	Inside a car, when children are present, do you think that smoking should be:	Support (always allowed); not support (allowed under some conditions, never allowed).	n = 164,819	94.3%	
Al-Delaimy (2008)	California (USA)	Cars with children	Inside cars when children are traveling in them?	Please tell me if smoking should be allowed or not allowed in this place	n = 82,566	92.3% (91.6-93.0)	
Berg (2015)	Alabama, Florida, Georgia, Kentucky, North Carolina, Mississippi, South Carolina, Tennessee (USA)	Private vehicles when children under 18 are present	For each of the following places, indicate how you feel about a policy prohibiting smoking in that kind of place	Oppose (strongly oppose, somewhat oppose); other (neutral, somewhat favour, strongly favour	n = 2501	20.7%	79.3%
Berg (2016)	Republic of Georgia	In cars with children under 18 present	How would you feel about a policy prohibiting smoking in that kind of place?	Oppose (somewhat oppose, strongly oppose); other (~~neutral,~~ somewhat favour, strongly favour)	n = 1163	10.7%	89.3%
Boeckmann (2018)	Germany	Cars with children	When minor children are in the car, smoking inside the car should be banned and subjected to punishment?	Agree (tend to support, strongly oppose support); don't agree (no opinion, tend to	n = 2062	71.5% (69.5 - 73.5)	

Author (year)	Geographic location	Smoke-free location	Support measure	Answer options	Overall sample size	Reported support overall	Support transformed, reversed etc.
				oppose, strongly oppose)			
Cartanyà-Hueso (2019)	Barcelona (Spain)	Cars with children	Should smoking be prohibited in cars with children?	Yes (agree, strongly agree); no (neutral, disagree, totally disagree)	n = 736	89.5%	
Díez-Izquierdo (2017)	Spain	Cars with children	Should smoking be prohibited inside cars in the presence of minors?	Agree (agree, totally agree); disagree (disagree, totally disagree); neither agree nor disagree)	n = 1036	90.1%	
Fong (2013)	France	Cars with children	Do you support or oppose a French total ban on smoking in cars with children in them	Support (support, strongly support); oppose (oppose, strongly oppose)	Wave 1 = NA<br>Wave 2 = 2217<br>Wave 3 = 2193	Wave 1: NA<br>Wave 2: 90.0% (87.4-92.1)<br>Wave 3: 91.8% (89.6-93.6)	
Gendall (2013)	New Zealand	Cars with children	Indicate support for smoke-free cars with children	Eleven-point Likert scale	n = 828	88.0%	
Jalleh (2006)	Western Australia	Vehicles carrying children	Banning smoking in vehicles when children under 18 years are in the vehicle	In favour of; against; no opinion either way	n = 405	See subgroup smoking status	83.0%
Li (2013)	New Zealand	Cars with minors present	Smoking in cars should be banned where children are in them	Strongly agree; agree; neither agree nor disagree; disagree; strongly disagree	n = 2,672	Strongly agree:<br>64% (60 -67)<br>Agree: 29% (26 - 32)	93% (90-99)
Li (2016)	New Zealand	Cars with children	Smoking in cars should be banned when children are in them	Agree (Strongly agree, agree); not agree (neither agree	n = 2,594	97.3%	

Author (year)	Geographic location	Smoke-free location	Support measure	Answer options	Overall sample size	Reported support overall	Support transformed, reversed etc.
				nor disagree, disagree, strongly disagree)			
Lugo (2017)	Italy	Cars with minors or pregnant women	Are you in favour of the extension of smoking ban in private vehicles in presence of minors or pregnant women?	Favour (moderately favour, strongly favour); against (moderately against, strongly against0	n = 3000	91.3%	91.3%
Lund (2016)	Norway	In cars when children are present	What is your opinion if the government were to implement these regulations on smoking behaviour? Prohibit smoking in cars when children are present	Five-point Likert scale	n= 5543	Mean (SD) 4.47 (1.14)	89.0%
Martínez-Sánchez (2014)	Italy	Cars carrying children	Attitude towards smoke-free legislation in cars carrying children	Support (moderately in favour, strongly in favour); no support (moderately against, strongly against)	n = 6167	92.5%	
Nogueira (2022)	Bulgaria<br>England<br>France<br>Germany<br>Greece<br>Ireland<br>Italy<br>Latvia<br>Poland<br>Portugal	Cars with children	For each of the following sites, are you:	Strongly in favour, moderately in favour, moderately against or strongly against a total tobacco ban?	Bulgaria: n = 1,050<br>England: n = 1,013<br>France: n = 1,018<br>Germany: n = 1,031<br>Greece: n = 1,000<br>Ireland: n = 941<br>Italy: n = 1,059<br>Latvia: n = 1,022<br>Poland: n = 724<br>Portugal: n = 1,000	Strongly in favour: 54.4%<br>Moderately in favour : 17.1%	72.1%

Author (year)	Geographic location	Smoke-free location	Support measure	Answer options	Overall sample size	Reported support overall	Support transformed, reversed etc.
	Romania<br>Spain				Romania: n = 1,018<br>Spain: n = 1,026		
Rosen (2012)	Israel	Cars with kids	Support for smoke-free cars with kids	In favour (5-7); neutral (4); opposed (1-3)	n = 505	94.4%	
Sæbø (2019)	Norway	Cars with children	Ban smoking in cars where children are passenger	Five-point Likert scale	n=5543	78.0%	
Stillman (2018)	USA	Cars with children	Attitudes towards smoking in cars with children	Not allowed (should never be allowed); allowed (only under some conditions; should always be allowed)	Urban: n = 181.162<br>Rural: n = 47.805	Urban: 94.8%<br>Rural: 92.5%	94.0%
Topuridze (2020)	Armenia, Republic of Georgia	Private vehicles with children	To what extent do you support or oppose a complete cigarette smoking ban in the following settings?	1: strongly oppose<br>2: somewhat oppose<br>3: don't know<br>4: somewhat support<br>5: strongly support	Armenia: n = 705<br>Georgia: n = 751	Mean (SD)<br>4.75 (0.72)	93.7%
Walsh (2008)	New South Wales, Australia	Cars with children	Supporting government bans	Not reported	n = 1191	54.8% (52.0-57.6)	
**Outdoor private**							
Ruokolainen (2018)	Finland	Balcony	Smoking on balconies should be forbidden by law	Agree (agree, completely agree); disagree (neutral, disagree, completely disagree)	n = 4905	51.3%	
Sæbø (2019)	Norway	Private balconies	Ban smoking on private balconies in cases where it bothers the neighbour	Full support; partial support; no support	n = 5543	30.9%	

Author (year)	Geographic location	Smoke-free location	Support measure	Answer options	Overall sample size	Reported support overall	Support transformed, reversed etc.
Hewett (2012)	USA	Residents' patios decks and balconies	What rules do you prefer about smoking in residents' patios decks and balconies	Strongly prefer smoking not permitted; somewhat prefer not permitted; no preference; somewhat prefer permitted; strongly prefer permitted	n = 414	Strongly prefer smoking not permitted: 28.3% (22.8-34.4) Somewhat prefer smoking not permitted:12.4 (9.0-17.0)	40.7%
**Outdoor semi-private**							
Berg (2015)	Alabama, Florida, Georgia, Kentucky, North Carolina, Mississippi, South Carolina, Tennessee (USA)	Outdoor common areas of apartment complexes	For each of the following places, indicate how you feel about a policy prohibiting smoking in that kind of place	Oppose (strongly oppose, somewhat oppose); other (neutral, somewhat favour, strongly favour	n = 2501	30.0%	70.0%
Berg (2015)	Alabama, Florida, Georgia, Kentucky, North Carolina, Mississippi, South Carolina, Tennessee (USA)	Outdoor common areas of town home or condo complexes	For each of the following places, indicate how you feel about a policy prohibiting smoking in that kind of place	Oppose (strongly oppose, somewhat oppose); other (neutral, somewhat favour, strongly favour	n = 2501	28.3%	71.7%
Berg (2016)	Republic of Georgia	Outdoor common areas of apartments	How would you feel about a policy prohibiting smoking in that kind of place?	Oppose (somewhat oppose, strongly oppose); other (~~neutral,~~ somewhat favour, strongly favour)	n = 1163	23.8%	76.2%

Author (year)	Geographic location	Smoke-free location	Support measure	Answer options	Overall sample size	Reported support overall	Support transformed, reversed etc.
Hewett (2012)	Minnesota (USA)	Outdoor common areas	What rules do you prefer about smoking in outdoor common areas	Strongly prefer smoking not permitted; somewhat prefer not permitted; no preference; somewhat prefer permitted; strongly prefer permitted	n = 429	Strongly prefer smoking not permitted: 32.8% (9.3-16.8) Somewhat prefer smoking not permitted: 5.0% (3.2-7.7)	37.8% (12.5-24.5)
Topuridze (2020)	Armenia, Republic of Georgia	Outdoor common areas	To what extent do you support or oppose a complete cigarette smoking ban in the following settings?	1: strongly oppose<br>2: somewhat oppose<br>3: don't know<br>4: somewhat support<br>5: strongly support	Armenia: n = 705<br>Georgia: n = 751	Mean (SD) 3.76 (1.43)	69.0%
**Indoor semi-private – multi-unit housing**							
Berg (2015)	Alabama, Florida, Georgia, Kentucky, North Carolina, Mississippi, South Carolina, Tennessee (USA)	Indoor common areas of apartment complexes like hallways, lobbies, and stairwells	For each of the following places, indicate how you feel about a policy prohibiting smoking in that kind of place	Oppose (strongly oppose, somewhat oppose); other (neutral, somewhat favour, strongly favour	n = 2501	15.8%	84.2%
Berg (2016)	Republic of Georgia	Indoor common areas of apartments	How would you feel about a policy prohibiting smoking in that kind of place?	Oppose (somewhat oppose, strongly oppose); other (~~neutral,~~ somewhat favour, strongly favour)	n = 1163	24.2%	75.8%

Author (year)	Geographic location	Smoke-free location	Support measure	Answer options	Overall sample size	Reported support overall	Support transformed, reversed etc.
Díez-Izquierdo (2017)	Spain	Multi-unit housing	Should smoking be prohibited within the common areas (i.e., elevator, stairs, lobby, walkways etc.) of multi-unit housing	Agree (agree, totally agree); disagreed (disagree, totally disagree); neither agree nor disagree)	n = 1036	85.7%	
Drach (2010)	Portland, Oregon, (USA)	Housing buildings	Not reported	Support (very happy, somewhat happy with policy); not reported	n = 688	74%	
Gentzke (2018)	Columbia, Charleston, Grand Forks, Bismarck, Fort Collins and Pueblo (USA)	Overall multi-unit housing	Would you prefer to have a policy in your building that prohibits smoking in all indoor areas, including individual residential units and common indoor areas?	Yes; no	n = 1565	56.4%	
Gentzke (2018)	Columbia, Charleston, Grand Forks, Bismarck, Fort Collins and Pueblo (USA)	Multi-unit housing buildings in Columbia	Would you prefer to have a policy in your building that prohibits smoking in all indoor areas, including individual residential units and common indoor areas?	Yes; no	n = 358	56.3%	
Gentzke (2018)	Columbia, Charleston, Grand Forks, Bismarck, Fort Collins and Pueblo (USA)	Multi-unit housing buildings in Charleston	Would you prefer to have a policy in your building that prohibits smoking in all indoor areas, including individual residential units and common indoor areas?	Yes; no	n = 318	63.7%	
Hewett (2012)	Minnesota (USA)	Corridors, stairways and lobbies	What rules do you prefer about smoking in	Strongly prefer smoking not	n = 314	Strongly prefer	82.0%

Author (year)	Geographic location	Smoke-free location	Support measure	Answer options	Overall sample size	Reported support overall	Support transformed, reversed etc.
			corridors, stairways and lobbies	permitted; somewhat prefer not permitted; no preference; somewhat prefer permitted; strongly prefer permitted		smoking not permitted: 73.7% (66.4-79.9) Somewhat prefer smoking not permitted: 8.3% (5.0-13.5)	
Kennedy (2015)	Canada	Multi-unit housing (pre-policy, 2010)	Support for the new policy making all new leases 100% smoke free	Not reported	n = 685	See subgroup smoking status	72.0%
Kennedy (2015)	Canada	Multi-unit housing (post-policy, 2011)	Support for the new policy making all new leases 100% smoke free	Not reported	n = 645	See subgroup smoking status	77.3%
Kennedy (2015)	Canada	Multi-unit housing (post-policy, 2013)	Support for the new policy making all new leases 100% smoke free	Not reported	n = 585	See subgroup smoking status	78.3%
King (2010)	New York (USA)	Public housing	Favour a policy in your building that bans smoking in all areas, including personal living spaces, such as balconies and patios?	In favour (probably yes, definitely yes); against (no opinion, probably not, definitely not)	n = 5936	55.6%	
Licht (2012)	USA	Multi-unit housing building	Do you think landlords should prohibit smoking inside all areas of their building, including apartments and common areas?	Yes; no	n = 418	56.0%	
McMillen (2019)	USA	Indoor multi-unit housing	Smoking should not be allowed anywhere inside	Supportive (agree, strongly agree);	n = 3070	70.8%	

Author (year)	Geographic location	Smoke-free location	Support measure	Answer options	Overall sample size	Reported support overall	Support transformed, reversed etc.
			buildings that have Section 8 housing units	unsupportive (disagree; strongly disagree)			
Meng (2016)	Los Angeles, CA, (USA)	Housing buildings	Preference for living in a non-smoking part of entirely smoke-free building	Not reported	n = 985	82%	
Meng (2016)	Los Angeles, CA, (USA)	Housing buildings	Smoke-free policies should apply to both new and existing buildings	Not reported	n = 985	95%	
Patel (2022)	USA	Multiunit housing	Support for a policy prohibiting smoking in multiunit housing	Strongly support, support, oppose, strongly oppose	n = 583	77.9%	
Rosen (2012)	Israel	Common areas apartment buildings	Support for smoke-free common areas apartment buildings	In favour (5-7); neutral (4); opposed (1-3)	n = 505	82.3%	
Schmidt (2016)	Montana (USA)	Multi-unit housing	Whether or not they agreed with a rule that does not allow smoking in their apartment, in all indoor common areas and in all outdoor areas	Support (agree, strongly agree); no support (disagree, strongly disagree)	n = 685	80.6%	
Topuridze (2020)	Armenia, Republic of Georgia	Indoor common areas	To what extent do you support or oppose a complete cigarette smoking ban in the following settings?	1: strongly oppose<br>2: somewhat oppose<br>3: don't know<br>4: somewhat support<br>5: strongly support	Armenia: n = 705<br>Georgia: n = 751	Mean (SD) 3.99 (1.83)	74.7%
Wang (2018)	USA	Public housing	Do you favour or oppose prohibiting smoking in public housing, including	Strongly favour; somewhat favour;	n = 4203	Strongly favour:	73.7% (CI 72.1 - 75.4)

Author (year)	Geographic location	Smoke-free location	Support measure	Answer options	Overall sample size	Reported support overall	Support transformed, reversed etc.
			all indoor areas of living units, common areas, and office buildings, as well as in all outdoor areas within 25 feet of buildings?	somewhat oppose; strongly oppose		52.3% (50.5 - 54.2) Somewhat favour: 21.4% (19.9 - 22.9)	
**Indoor semi-private - other**							
Brown (2019)	Scotland	Prison	Smoking should not be allowed in any outdoor areas of prisons	Agree (agree, strongly agree); disagree (no opinion, disagree, strongly disagree)	Staff: n = 1269 Prisoners: n = 2411	Staff: 37.8% Prisoners: 17.9%	25.0%
Buth (2013)	Germany	Prisons	Approval of a total smoking ban (including cells)	Not reported	n = 1258	9.9%	9.9%
Bower (2005)	Midwestern USA	Campus Apartments	All campus apartments should be non-smoking?	Strongly agree; agree; somewhat agree; undecided; somewhat disagree; disagree; strongly disagree	Freshman: n = 1743	Strongly agree: 35.3 % Agree: 7.8% Somewhat agree: 6.4%	49.5%
Bower (2005)	Midwestern USA	Campus Apartments	All campus apartments should be non-smoking?	Strongly agree; agree; somewhat agree; undecided; somewhat disagree; disagree; strongly disagree	Junior: n = 643	Strongly agree: 40.3% Agree: 5.3% Somewhat agree: 6.5%	52.1%
Garg (2011)	Berkeley, CA (USA)	Student residences	How important is it to you that your residential community adopts a smoke-free policy (no smoking anywhere, inside	Very important; somewhat important; somewhat unimportant; very unimportant	n = 1479	Very important: 46% Somewhat important: 20%	66.0%

Author (year)	Geographic location	Smoke-free location	Support measure	Answer options	Overall sample size	Reported support overall	Support transformed, reversed etc.
			or outside, on the premises)?				
Hammond (2006)	Ontario (Canada)	Student residences	Do you oppose or support campus smoking restrictions in student residences?	Support (1,2); neutral (3); oppose (4,5)	n = 1690	88%	88%
Sweeting (2021)	Scotland (UK)	Prisons	Prison smoking bans are a good idea	Agree (agree, strongly agree); not agree (neutral, disagree, strongly disagree)	Prisoners:<br>Phase 1: n = 2512<br>Phase 2: n = 1959<br>Phase 3: n = 1485<br>Staff:<br>Phase 1: n = 1271<br>Phase 2: n = 1494<br>Phase 3: n= 757	Prisoners:<br>Phase 1: 23.5%<br>Phase 2: 25.0%<br>Phase 3: 27.3%<br>Staff:<br>Phase 1: 79.0%<br>Phase 2: 69.9%<br>Phase 3: 83.7%	46.3%
Topuridze (2020)	Armenia, Republic of Georgia	Taxis	To what extent do you support or oppose a complete cigarette smoking ban in the following settings?	1: strongly oppose<br>2: somewhat oppose<br>3: don't know<br>4: somewhat support<br>5: strongly support	Armenia: n = 705<br>Georgia: n = 751	Mean (SD) 4.57 (1.01)	89.3%
Xiao (2020)	China	Taxis	Not reported	Support; no support	Not reported	87.2% (84.5-89.5)	
**Outdoor non hospitality – campus**							

Author (year)	Geographic location	Smoke-free location	Support measure	Answer options	Overall sample size	Reported support overall	Support transformed, reversed etc.
Almutairi (2014)	Saudi Arabia	University Campus	Our campus should be completely smoke free	Likert scale	Students: n = 3578<br>Faculty: n = 597<br>Staff: n = 416	See subgroups	90.0%
Atiba (2020)	Lagos State (Nigeria)	University campus	In favour of banning smoking at outdoor places on campus	Yes; unsure; no	n = 401	61.4%	
Bartington(2020)	Birmingham (UK)	University campus	We should aspire to make the university free of tobacco smoking	Agree (agree, strongly agree); disagree (unsure, disagree, strongly disagree)	Students: n = 235<br>Staff: n = 545	Students: 80.6% (74.4 - 85.9%)<br>Staff: 72.6% (68.3 - 76.6%)	75.0%
Berg (2015)	Alabama, Florida, Georgia, Kentucky, North Carolina, Mississippi, South Carolina, Tennessee (USA)	All outdoor areas on college or university campuses	For each of the following places, indicate how you feel about a policy prohibiting smoking in that kind of place	Oppose (strongly oppose, somewhat oppose); other (neutral, somewhat favour, strongly favour	n = 2501	28.3%	71.7%
Berg (2016)	Republic of Georgia	Outdoor areas on college campuses	How would you feel about a policy prohibiting smoking in that kind of place?	Oppose (somewhat oppose, strongly oppose); other (~~neutral,~~ somewhat favour, strongly favour)	n = 1163	11.4%	88.6%
Berg (2020)	Massachusetts, USA	Two college campuses; one with a smoking ban (SBC) and control campus without a policy (CC)	If your college were to enact [such] a campus-wide smoking ban (banning smoking from all indoor and outdoor spaces on campus), how would you feel about this policy?	1 (strongly agree/very positive) to 5 (strongly disagree/very negative) recoded so higher rates represent more less	Pre-ban:<br>SBC: n = 192<br>CC: n = 226<br>Post-ban:<br>SBC: n= 372<br>CC: n = 268	mean (SD)<br>Pre-ban:<br>SBC: 2.52 (1.50)<br>CC: 2.59 (1.55)<br>Post-ban:<br>SCB: 1.17	Pre-ban: 61.1%<br>Post-ban: 76.1%

Author (year)	Geographic location	Smoke-free location	Support measure	Answer options	Overall sample size	Reported support overall	Support transformed, reversed etc.
				positive views towards a campus-wide ban		(1.08)<br>CC: 2.3 (1.42)	
Bower (2005)	Midwestern USA	Campus tobacco-free	The campus should be a tobacco (including smokeless tobacco) free campus?	Strongly agree; agree; somewhat agree; undecided; somewhat disagree; disagree; strongly disagree	Freshman: n = 1743	Strongly agree: 20.1%<br>Agree: 5.2%<br>Somewhat agree: 7.7%	33.0%
Bower (2005)	Midwestern USA	Campus tobacco-free	The campus should be a tobacco (including smokeless tobacco) free campus?	Strongly agree; agree; somewhat agree; undecided; somewhat disagree; disagree; strongly disagree	Junior: n = 643	Strongly agree: 21.2 %<br>Agree: 7.0%<br>Somewhat agree: 7.2%	35.4%
Braverman (2014)	USA	College campus	Our campus should be 100% smoke-free.	Supportive (agree, strongly agree); opposed (neutral, disagree, strongly disagree)	Students: n = 5691	Support: 71.6% (70 - 73)<br>Neutral: 6.3% (6 - 7)<br>Oppose: 22.1% (21 -23)	71.6%
Braverman (2015)	USA	College campus	Our campus should be 100% smoke-free.	Supportive (agree, strongly agree); opposed (neutral, disagree, strongly disagree)	Staff: n = 2051	Support; 77.1% (75 - 79)<br>Neutral: 5.8% (5 - 7)<br>Oppose: 17.1% (15 - 19)	77.1%
Braverman (2021)	Oregon, USA	College Campus	2013: Our campus should be 100% smoke-free	1 (strongly disagree) to 7 (strongly agree)	2013:<br>Students: n = 5691	2013:<br>Students:	Pre ban:<br>73.0%

Author (year)	Geographic location	Smoke-free location	Support measure	Answer options	Overall sample size	Reported support overall	Support transformed, reversed etc.
			2018: Our campus should be 100% smoke-free, including cigarettes, other smoking products, and vaping products	collapsed into oppose, neutral and support	Faculty/staff: n = 2051<br>2018:<br>Students: n = 4883<br>Faculty/staff: n = 1882	71.6%<br>Faculty/staff: 77.0%<br>2018:<br>Students: 73.2%<br>Faculty/staff: 83.7%	Post ban: 76.1%
Burns (2013)	Perth (Australia)	College campus	Our campus should be smoke-free including all outdoor areas	Agree (agree, strongly agree); neutral; disagree (disagree, strongly disagree)	n = 589	60.8%	
Burns (2013)	Perth (Australia)	College campus	Our campus should be completely smoke free	Agree (agree, strongly agree); neutral; disagree (disagree, strongly disagree)	n = 636	65.7%	
Cartanyà-Hueso (2019)	Barcelona (Spain)	Outdoor areas of universities	Should smoking be prohibited at outdoor areas of universities?	Yes (agree, totally agree); no (neutral, disagree, totally disagree)	n = 736	57.7%	
Chaaya (2021)	Beirut (Lebanon)	University Campus	Students were asked to indicate the extent to which they supported the policy	Agree (large extent, some extent); not agree (not at all, not sure)	Pre-ban: n = 809<br>Post-ban: n = 615	See subgroup smoking status	Pre-ban: 62.7%<br>Post-ban: 83.7%
El Ansari (2012a)	Egypt	University Campus	There should be no smoking on the university premises at all	Agree (agree, strongly agree); disagree (neutral, disagree, strongly disagree)	n = 3258	87.3%	

Author (year)	Geographic location	Smoke-free location	Support measure	Answer options	Overall sample size	Reported support overall	Support transformed, reversed etc.
El Ansari (2012b)	UK, Wales, Northern Ireland	University Campus	There should be no smoking on the university premises at all	Agree (agree, strongly agree); disagree (neutral, disagree, strongly disagree)	n = 3706	45.3%	
El Ansari (2021)	Finland	University	There should be no smoking on university premises at all	5 point Likert scale, strongly agree to strongly disagree	n = 1177	Strongly agree/agree: 48.6% Neutral: 31.5% Strongly disagree/disagree: 19.8%	48.6%
Fallin (2015)	California (USA)	University Campus	Prohibiting all tobacco use on the entire grounds (tobacco-free policy) is a good thing	Agree (agree, strongly agree); disagree (neutral, disagree, strongly disagree)	n = 217	77%	
Fallin (2015)	California (USA)	University Campus	Prohibiting smoking on the entire grounds (smoke-free policy) is a good thing	Agree (agree, strongly agree); disagree (neutral, disagree, strongly disagree)	n = 230	67%	
Fallin (2015)	California (USA)	University Campus	Prohibiting smoking 30ft from entrances (California state law) is a good thing	Agree (agree, strongly agree); disagree (neutral, disagree, strongly disagree)	n = 217	71%	
Farran (2021)	Lebanon	University	Extent of support for the university tobacco-free policy	4-point Likert scale (large extent, some	2017: n = 625 2018: n = 624	Pre ban: 79% Post ban: 84%	84%

Author (year)	Geographic location	Smoke-free location	Support measure	Answer options	Overall sample size	Reported support overall	Support transformed, reversed etc.
				extent, not at all, not sure)			
Hammond (2006)	Ontario (Canada)	Student residences	Do you oppose or support campus smoking restrictions in student residences?	Support (1,2); neutral (3); oppose (4,5)	n = 1,690	88%	
Hammond (2006)	Ontario (Canada)	University campus	Do you oppose or support campus smoking restrictions on the entire campus?	Support (1,2); neutral (3); oppose (4,5)	n = 1,690	41%	
Ickes (2017)	USA	University campus	How would you rate your feelings about the tobacco-free policy + what do you think the impact is of having a tobacco free campus?	2x Five-point Likert scale summed up	n = 660	Mean (SD) 8.4 (1.9)	84%
Johnston Polacek (2008)	USA	University campus	How likely would you be to support a policy change to make this campus smoke-free?	5-point Likert scale	Undergraduate: n = 2486<br>Graduate: n = 173<br>Staff: n = 109<br>Faculty: n = 51	Undergraduate: 3.49<br>Graduate: 4.09<br>Staff: 3.86<br>Faculty: 3.53	72.0%
Karadağ (2021)	Turkey	University	What do you think about the “Tobacco-Free Campus Policy”, which includes not using tobacco in indoor and outdoor areas within the university campus, not selling tobacco in the campus, and providing	I definitely support; it could be; it would be an unnecessary restriction; finds unacceptable, opposes	n = 10,383	I definitely support: 49.9%<br>It could be: 13.8%<br>It would be an unnecessary restriction:	49.9%

Author (year)	Geographic location	Smoke-free location	Support measure	Answer options	Overall sample size	Reported support overall	Support transformed, reversed etc.
			assistance to those who want to quit smoking?			22.6% Finds unacceptable, opposes: 13.6%	
Kecojevic (2020)	New Jersey (USA)	College campus	The university should prohibit the use of all tobacco products on campus	0 = disagree to 4 = strongly agree	n = 1,192	Mean (SD): 2.3 (1.5)	52.5%
Lechner (2012)	Midwestern USA	University campus (2007)	Campus should be smoke free	7-point Likert scale	n = 1185	Mean (SD) 4.57 (2.43)	65.3%
Lechner (2012)	Midwestern USA	University campus (2008)	Campus should be smoke free	7-point Likert scale	n = 1197	5.33 (2.22)	76.1%
Lechner (2012)	Midwestern USA	University campus (2009)	Campus should be smoke free	7-point Likert scale	n = 1257	5.47 (2.04)	78.1%
Lechner (2012)	Midwestern USA	University campus (2010)	Campus should be smoke free	7-point Likert scale	n = 1257	5.77 (2.04)	82.4%
Loukas (2006)	Texas (USA)	University campus	Our campus should be completely smoke-free	Four-point Likert scale	n = 1188	See subgroup gender	68.7%
Mamudu (2016)	Johnson City, Tennessee (USA)	University campus	Support for ETSU Tobacco-free policy	Yes; no	n = 790	38.7%	
Mamudu (2016)	Johnson City, Tennessee (USA)	University campus	Support for ETSU Tobacco-free campus	Yes; no	n = 790	40.1%	
Marsh (2014)	Dunedin (New Zealand)	University campus	Indicate your support for the following university policies: a complete smoke free campus (i.e. no smoking on any university grounds)	Eleven-point scale	Staff: n = 332	8.0	
Marsh (2014)	Dunedin (New Zealand)	University campus	Indicate your support for the following university	Eleven-point scale	Students : n = 268	7.7	77.0%

Author (year)	Geographic location	Smoke-free location	Support measure	Answer options	Overall sample size	Reported support overall	Support transformed, reversed etc.
			policies: a complete smoke free campus (i.e. no smoking on any university grounds)				
Mishra (2011)	USA	University campus	Believe that entire campus should be smoke-free	Not reported	Faculty: n = 193<br>Staff: n = 291<br>Graduate: n = 317<br>Undergraduate: n = 2088	Faculty: 75%<br>Staff: 73.0%<br>Graduate: 71.0%<br>Undergraduate: 56.0%	61.0%
Niemeier (2014)	Midwestern USA	College campus	Would you be in support of the campus going completely smoke-free	Yes; no	n = 1266	See subgroup smoking status	63.0%
Rosen (2012)	Israel	College campuses	Support for smoke-free college campuses	In favour (5-7); neutral (4); opposed (1-3)	n = 505	36.5%	
Ramachandran (2020)	Mississippi (USA)	University campus	Smoking should be banned on all university property	1 (strongly disagree) to 5 (strongly agree)	n = 1541	Mean (SD): 3.6 (1.3)	65.0%
Sabrian (2019)	Riau (Indonesia)	University campus	Perception regarding smoke-free campus policy	Agree(strongly agree; somewhat agree) disagree (somewhat disagree, disagree)	Students: n = 880	Strongly agree = 88.3%<br>Somewhat agree = 7.8%	96.1%
Sabrian (2019)	Riau (Indonesia)	University campus	Perception regarding smoke-free campus policy	Agree(strongly agree; somewhat agree) disagree (somewhat disagree, disagree)	Staff: n = 229	Strongly agree = 82.1%<br>Somewhat agree = 11.4%	93.5%
Sabrian (2019)	Riau (Indonesia)	University campus	Perception regarding smoke-free campus policy	Agree(strongly agree; somewhat agree) disagree	Lecturers: n = 102	Strongly agree = 86.3%<br>Somewhat agree = 12.7%	99.0%

Author (year)	Geographic location	Smoke-free location	Support measure	Answer options	Overall sample size	Reported support overall	Support transformed, reversed etc.
				(somewhat disagree, disagree)			
Sendall (2021)	Queensland (Australia)	University campus	Our campus should be smoke free including all outdoor areas	Agree (agree, strongly agree); neutral; disagree(disagree, strongly disagree)	n = 614	85.6% (81.4-89.9)	
Sureda (2015)	Spain	University campuses	To what extent do you agree or disagree with the prohibition of smoking in the following outdoor settings?	Agree (agree, totally agree); neither agree nor disagree; disagree (disagree, totally disagree)	n = 1300	52.7%	
Thompson (2006)	Idaho, Oregon, Washington (USA)	University campus	Outdoor smoking policies, smoking should be:	Not allowed anywhere; only in designated areas; allowed anywhere; other	n = 14,167	See subgroup smoking status	33.1%
Topuridze (2020)	Armenia, Republic of Georgia	Outdoor areas university	To what extent do you support or oppose a complete cigarette smoking ban in the following settings?	1: strongly oppose<br>2: somewhat oppose<br>3: don't know<br>4: somewhat support<br>5: strongly support	Armenia: n = 705<br>Georgia: n = 751	4.42 (1.14)	85.5%
Wallar (2013)	Guelph (Canada)	University campus	100 % smoke-free campus	Strongly support; support; neutral; oppose; strongly oppose	n = 492.301	Strongly support: 29.1%<br>Support: 22.2%	51.3%
Williams (2011)	USA	University campus	The university should have a policy making the entire campus smoke free	Likert-scale from -2 to +2, with positive values representing	Students: n = 985<br>Employees: n = 58	See gender subgroup	65.1%

Author (year)	Geographic location	Smoke-free location	Support measure	Answer options	Overall sample size	Reported support overall	Support transformed, reversed etc.
				beliefs that are favourable towards smoke-free environments	Community members: n = 28		
Wipfli (2020)	Australia, Bangladesh, China, Indonesia, Japan, Korea, Malaysia, New Zealand, Philippines, Russia, Singapore, Thailand, USA	University campus	Support smoke-free campuses?	Yes; no	Australia: n = 4<br>Bangladesh: n = 474<br>China: n = 221<br>Indonesia: n = 4<br>Japan: n = 102<br>Korea: n = 521<br>Malaysia: n = 145<br>New Zealand: n =22<br>Philippines: n = 113<br>Russia: n = 218<br>Singapore: n = 3<br>Thailand: n = 68<br>USA: n = 58	All countries: 79.3%	
Wong (2020)	California (USA)	University campus	Would you favour or oppose a tobacco policy that would prohibit the use of all tobacco products on core campus, including all outdoor areas within the perimeter of Campus Drive?	Favour; neither favour nor oppose; oppose	n = 2218	63%	
Wray (2020)	Missouri (USA)	University campus	Support for campus-wide tobacco-free-policy	Not reported	**2016:**<br>Total: n = 2277<br>Students: n = 1332<br>**2017:**	**Overall:**<br>2016: 75%<br>2017: 84%<br>**Students:**	

Author (year)	Geographic location	Smoke-free location	Support measure	Answer options	Overall sample size	Reported support overall	Support transformed, reversed etc.
					Total: n = 1206<br>Students: n = 55	2016: 72%<br>2017: 78%	
**Outdoor non hospitality – beaches, parks and playgrounds**							
Al-Delaimy (2008)	California (USA)	Parks, beaches, golf courses, zoos sports stadiums	Outdoor public places such as parks, beaches, golf course, zoos, sports stadiums	Please tell me if smoking should be allowed or not allowed in this place	n = 82,566	53.4% (51.3 – 55.5)	
Abundis (2008)	Mexico	Beach	Do you agree or disagree with the prohibition of smoking in the following places (…)?	Agree; disagree	n = 1200	47.0%	
Basto-Abreu (2016)	Baja California (Mexico)	Beaches	Do you support the state level law that banned smoking in all outdoor areas where people gather for beaches?	Yes; no	n = 796	81.0%	
Basto-Abreu (2016)	Baja California (Mexico)	Parks	Do you support the state level law that banned smoking in all outdoor areas where people gather for parks?	Yes; no	n = 796	89.0%	
Berg (2015)	Alabama, Florida, Georgia, Kentucky, North Carolina, Mississippi, South Carolina, Tennessee (USA)	Public parks, playgrounds, and beaches	For each of the following places, indicate how you feel about a policy prohibiting smoking in that kind of place	Oppose (strongly oppose, somewhat oppose); other (neutral, somewhat favour, strongly favour	n = 2501	28.5%	71.5%
Berg (2016)	Republic of Georgia	Public parks, playgrounds, beaches	How would you feel about a policy prohibiting smoking in that kind of place?	Oppose (somewhat oppose, strongly oppose); other (~~neutral,~~ somewhat	n = 1163	26.6%	73.4%

Author (year)	Geographic location	Smoke-free location	Support measure	Answer options	Overall sample size	Reported support overall	Support transformed, reversed etc.
				favour, strongly favour)			
Bo-Woo (2012)	Daejeon (Korea)	Parks/Playgrounds	Should smoke-free zones be implemented at parks and playgrounds	Agree; disagree	n = 1013	87.0%	
Cartanyà-Hueso (2019)	Barcelona (Spain)	Playgrounds	Should smoking be prohibited at playgrounds?	Yes (agree, totally agree); no (neutral, disagree, totally disagree)	n = 736	84.7%	
Cartanyà-Hueso (2019)	Barcelona (Spain)	Beaches and outdoor swimming pools	Should smoking be prohibited at beaches and outdoor swimming pools?	Yes (agree, totally agree); no (neutral, disagree, totally disagree)	n = 736	50.3%	
Dono (2015)	South Australia	Playgrounds (2012, pre-implementation)	Smoking ban at children's playgrounds	Approved (approve, strongly approve); disapproved (disapproved, strongly disapproved	n = 2005	94.8%	
Dono (2015)	South Australia	Playgrounds (2012, post-implementation)	Smoking ban at children's playgrounds	Approved (approve, strongly approve); disapproved (disapproved, strongly disapproved	n = 2013	97.8%	
Gallus (2012)	Italy	Public parks	Would you be in favour or against (the extension of) the smoking ban in public parks	Favour (moderately in favour, strongly in favour); oppose (moderately against, strongly against)	n = 6233	64.6%	
Gallus (2012)	Italy	Beaches	Would you be in favour or against (the extension of) the smoking ban on beaches	Favour (moderately in favour, strongly in favour); oppose (moderately against, strongly against)	n = 6233	62.1%	

Author (year)	Geographic location	Smoke-free location	Support measure	Answer options	Overall sample size	Reported support overall	Support transformed, reversed etc.
Gendall (2013)	New Zealand	Children's playground	Indicate support for smoke-free playgrounds	Eleven-point scale	n = 828	82.0%	
Gendall (2013)	New Zealand	Sports ground	Indicate support for smoke-free sport grounds	Eleven-point scale	n = 828	68.0%	
Gendall (2013)	New Zealand	Beaches	Indicate support for smoke-free outside building entrances and doorways	Eleven-point scale	n = 828	62.0%	
Gendall (2013)	New Zealand	Town or city parks and reserves	Indicate support for smoke-free town or city parks and reserves	Eleven-point scale	n = 828	61.0%	
Klein (2007)	Minnesota (USA)	Parks	Generally favour smoke-free park policies	Strongly agree; somewhat agree; somewhat disagree; strongly disagree	n = 1492	Strongly agree: 43% Somewhat agree: 26%	69%
Klein (2007)	USA	Parks all times	Smoking should be prohibited in all outdoor parks at all times	Strongly agree; somewhat agree; somewhat disagree; strongly disagree	n = 1488	Strongly agree: 34% Somewhat agree: 19%	53%
Kruger (2016)	USA	Parks	Should smoking at parks be:	Never allowed; allowed only at some places; allowed	n = 97,978	38.5% (37.9 - 39.2)	38.5% (37.9 - 39.2)
Lund (2016)	Norway	Public parks /gardens	What is your opinion if the government were to implement these regulations on smoking behaviour? Prohibit smoking at public parks /gardens	Five-point Likert scale	n= 5543	Mean (SD) 3.09 (1.64)	52.3%
Martínez-Sánchez (2014)	Italy	Parks and public gardens	Attitude towards smoke-free legislation in parks and public gardens	Support (moderately in favour, strongly in favour); no support	n =6167	60.5%	

Author (year)	Geographic location	Smoke-free location	Support measure	Answer options	Overall sample size	Reported support overall	Support transformed, reversed etc.
Martínez-Sánchez (2014)	Italy	Beaches	Attitude towards smoke-free legislation in beaches	(moderately against, strongly against) Support (moderately in favour, strongly in favour); no support (moderately against, strongly against)	n =6167	59.4%	
McMillen (2004)	USA	Parks	Outdoor parks should be smoke-free	Support(All areas); no support(some areas, not at all)	Large central: n = 639<br>Large fringe: n = 590<br>Small urban: n = 998<br>Rural with a city: n = 352<br>Rural without city: n = 429	Large central: 40.3%<br>Large fringe: 33.4%<br>Small urban: 38.7%<br>Rural with a city: 33.5%<br>Rural without city: 37.8%	37.3%
Nogueira (2022)	Bulgaria<br>England<br>France<br>Germany<br>Greece<br>Ireland<br>Italy<br>Latvia<br>Poland<br>Portugal<br>Romania<br>Spain	Parks	For each of the following sites, are you:	Strongly in favour, moderately in favour, moderately against or strongly against a total tobacco ban?	Bulgaria: n = 1,050<br>England: n = 1,013<br>France: n = 1,018<br>Germany: n = 1,031<br>Greece: n = 1,000<br>Ireland: n = 941<br>Italy: n = 1,059<br>Latvia: n = 1,022<br>Poland: n = 724<br>Portugal: n = 1,000<br>Romania: n = 1,018<br>Spain: n = 1,026	Strongly in favour: 27.7%<br>Moderately in favour: 20.1%	47.8%
Nogueira (2022)	Bulgaria	Playground	For each of the following sites, are you:	Strongly in favour, moderately in	Bulgaria: n = 1,050	Strongly in	70.7%

Author (year)	Geographic location	Smoke-free location	Support measure	Answer options	Overall sample size	Reported support overall	Support transformed, reversed etc.
	England<br>France<br>Germany<br>Greece<br>Ireland<br>Italy<br>Latvia<br>Poland<br>Portugal<br>Romania<br>Spain			favour, moderately against or strongly against a total tobacco ban?	England: n = 1,013<br>France: n = 1,018<br>Germany: n = 1,031<br>Greece: n = 1,000<br>Ireland: n = 941<br>Italy: n = 1,059<br>Latvia: n = 1,022<br>Poland: n = 724<br>Portugal: n = 1,000<br>Romania: n = 1,018<br>Spain: n = 1,026	favour: 53.4%<br>Moderately in favour: 17.3%	
Nogueira (2022)	Bulgaria<br>England<br>France<br>Germany<br>Greece<br>Ireland<br>Italy<br>Latvia<br>Poland<br>Portugal<br>Romania<br>Spain	Beaches	For each of the following sites, are you:	Strongly in favour, moderately in favour, moderately against or strongly against a total tobacco ban?	Bulgaria: n = 1,050<br>England: n = 1,013<br>France: n = 1,018<br>Germany: n = 1,031<br>Greece: n = 1,000<br>Ireland: n = 941<br>Italy: n = 1,059<br>Latvia: n = 1,022<br>Poland: n = 724<br>Portugal: n = 1,000<br>Romania: n = 1,018<br>Spain: n = 1,026	Strongly in favour: 27.7 %<br>Moderately in favour: 20.0%	47.7%
Okoli (2013)	Vancouver (Canada)	Parks and beaches	Would you say that you strongly support, somewhat support, somewhat oppose or strongly oppose smoke-	Supporting (somewhat support, strongly support); opposing (somewhat oppose, strongly oppose)	n = 500	84.2%	

Author (year)	Geographic location	Smoke-free location	Support measure	Answer options	Overall sample size	Reported support overall	Support transformed, reversed etc.
			free bylaws in parks and beaches in your city?				
Rosen (2012)	Israel	Beaches, parks, swimming pools	Support for smoke-free beaches, parks, swimming pools	In favour (5-7); neutral (4); opposed (1-3)	n = 505	29.7%	
Sæbø (2019)	Norway	Public parks	Ban smoking in all public parks	Five-point Likert scale	n=5543	34.2%	
Stevenson (2008)	Upper Hutt (New Zealand)	Parks	Having a smoke free parks policy was a good idea	Not reported	n = 587	83.0%	
Stillman (2018)	USA	Playgrounds	Attitude towards smoking in playgrounds	Not allowed (should never be allowed); allowed (only under some conditions; should always be allowed)	Urban: n = 181.162<br>Rural: n = 47.805	Urban: 88.4%<br>Rural: 84.4%	87.6%
Sureda (2015)	Spain	Playgrounds	To what extent do you agree or disagree with the prohibition of smoking in the following outdoor settings?	Agree (agree, totally agree); neither agree nor disagree; disagree (disagree, totally disagree)	n = 1301	80.8%	
Sureda (2015)	Spain	Swimming pools and beaches	To what extent do you agree or disagree with the prohibition of smoking in the following outdoor settings?	Agree (agree, totally agree); neither agree nor disagree; disagree (disagree, totally disagree)	n = 1296	43.0%	
Topuridze (2020)	Armenia, Republic of Georgia	Playgrounds	To what extent do you support or oppose a complete cigarette smoking ban in the following settings?	1: strongly oppose<br>2: somewhat oppose<br>3: don't know<br>4: somewhat support<br>5: strongly support	Armenia: n = 705<br>Georgia: n = 751	Mean (SD) 4.37 (1.10)	84.3%

Author (year)	Geographic location	Smoke-free location	Support measure	Answer options	Overall sample size	Reported support overall	Support transformed, reversed etc.
Topuridze (2020)	Armenia, Republic of Georgia	Parks and beaches	To what extent do you support or oppose a complete cigarette smoking ban in the following settings?	1: strongly oppose<br>2: somewhat oppose<br>3: don't know<br>4: somewhat support<br>5: strongly support	Armenia: n = 705<br>Georgia: n = 751	Mean (SD)<br>3.54 (1.47)	63.5%
Waddell (2014)	New York City (USA)	Parks	Opinion regarding prohibiting smoking in all public parks	Favour; oppose	2010: n = 1447<br>2011: n = 1436<br>2012: n = 1445	2010: 52%<br>2011: 46%<br>2012: 47%	
Waddell (2014)	New York City (USA)	Beaches	Opinion regarding prohibiting smoking on all public beaches	Favour; oppose	2010: n = 1447<br>2011: n = 1436<br>2012: n = 1446	2010: 48%<br>2011: 44%<br>2012: 50%	
Walsh (2008)	New South Wales (Australia)	Children's playground	Supporting government bans	Not reported	n = 1191	88.6% (86.0 - 90.4)	
Walsh (2008)	New South Wales (Australia)	Beaches	Supporting government bans	Not reported	n = 1191	55.5% (52.7 - 58.4)	
**Outdoor non hospitality – public transport stops**							
Berg (2015)	Alabama, Florida, Georgia, Kentucky, North Carolina, Mississippi, South Carolina, Tennessee (USA)	Bus stops	For each of the following places, indicate how you feel about a policy prohibiting smoking in that kind of place	Oppose (strongly oppose, somewhat oppose); other (neutral, somewhat favour, strongly favour	n = 2501	27.9%	72.1%
Berg (2016)	Republic of Georgia	Bus and train stops	How would you feel about a policy prohibiting smoking in that kind of place?	Oppose (somewhat oppose, strongly oppose); other (~~neutral,~~ somewhat favour, strongly favour)	n = 1163	29.9%	70.1%

Author (year)	Geographic location	Smoke-free location	Support measure	Answer options	Overall sample size	Reported support overall	Support transformed, reversed etc.
Bo-Woo (2012)	Daejeon (Korea)	Bus/Taxi stops	Should smoke-free zones be implemented at bus and taxi stops?	Agree; disagree	n = 1013	89.9%	
Cartanyà-Hueso (2019)	Barcelona (Spain)	Outdoor areas of public transport	Should smoking be prohibited in outdoor areas of public transport?	Yes (agree, totally agree); no (neutral, disagree, totally disagree)	n = 736	65.7%	
Dono (2015)	South Australia	Public transport stops	Smoking ban at transport stops such as bus, tram and train stops	Approved (approve, strongly approve); disapproved (disapproved, strongly disapproved	n = 2002	79.6%	79.6%
Dono (2015)	South Australia	Public transport stops (2012, pre-implementation)	Smoking ban at transport stops such as bus, tram and train stops	Approved (approve, strongly approve); disapproved (disapproved, strongly disapproved	n = 2005	78.3%	78.3%
Dono (2015)	South Australia	Public transport stops (2013, post-implementation	Smoking ban at transport stops such as bus, tram and train stops	Approved (approve, strongly approve); disapproved (disapproved, strongly disapproved	n = 2013	93.5%	93.5%
Li (2016)	New Zealand	Outdoor transport waiting areas	Smoking should be banned in all outdoor transport waiting areas	Agree (Strongly agree, agree); not agree (neither agree nor disagree, disagree, strongly disagree)	n = 2,954	74.6%	
Lund (2016)	Norway	Roofed stands for buses, trains, boats, trams and taxis	What is your opinion if the government were to implement these regulations on smoking behaviour? Prohibit	Five-point Likert scale (1 = no support, 5 = full support)	n= 5543	Mean (SD) 3.97 (1.44)	74.3%

Author (year)	Geographic location	Smoke-free location	Support measure	Answer options	Overall sample size	Reported support overall	Support transformed, reversed etc.
			smoking at roofed stands for buses, trains, boats, trams and taxis				
Martínez-Sánchez (2014)	Italy	Bus stops	Attitude towards smoke-free legislation in bus stops	Support (moderately in favour, strongly in favour); no support (moderately against, strongly against)	n = 6167	59.8%	
Nogueira (2022)	Bulgaria<br>England<br>France<br>Germany<br>Greece<br>Ireland<br>Italy<br>Latvia<br>Poland<br>Portugal<br>Romania<br>Spain	Tram/bus/subway stops,	For each of the following sites, are you:	Strongly in favour, moderately in favour, moderately against or strongly against a total tobacco ban?	Bulgaria: n = 1,050<br>England: n = 1,013<br>France: n = 1,018<br>Germany: n = 1,031<br>Greece: n = 1,000<br>Ireland: n = 941<br>Italy: n = 1,059<br>Latvia: n = 1,022<br>Poland: n = 724<br>Portugal: n = 1,000<br>Romania: n = 1,018<br>Spain: n = 1,026	Strongly in favour: 29.1%<br>Moderately in favour: 20.4%	49.5%
Rosen (2012)	Israel	Train platforms	Support for smoke-free train platforms	In favour (5-7); neutral (4); opposed (1-3)	n = 505	58.2%	
Sæbø (2019)	Norway	Covered stops or stations for bus, boat, tram, train, taxi and the like	What view would you take if the authorities were to propose these measures on smoking? Ban smoking in covered stops or stations for bus, boat, tram, train, taxi	Five-point Likert scale	n = 5543	58.1%	

Author (year)	Geographic location	Smoke-free location	Support measure	Answer options	Overall sample size	Reported support overall	Support transformed, reversed etc.
Sureda (2015)	Spain	Public transportation	To what extent do you agree or disagree with the prohibition of smoking in the following outdoor settings?	Agree (agree, totally agree); neither agree nor disagree; disagree (disagree, totally disagree)	n = 1305	56.1%	
Topuridze (2020)	Armenia, Republic of Georgia	Bus stops	To what extent do you support or oppose a complete cigarette smoking ban in the following settings?	1: strongly oppose<br>2: somewhat oppose<br>3: don't know<br>4: somewhat support<br>5: strongly support	Armenia: n = 705<br>Georgia: n = 751	Mean (SD) 4.10 (1.29)	77.5%
**Outdoor non hospitality – schools**							
Berg (2016)	Republic of Georgia	Outdoor areas on school grounds	How would you feel about a policy prohibiting smoking in that kind of place?	Oppose (somewhat oppose, strongly oppose); other (~~neutral,~~ somewhat favour, strongly favour)	n = 1163	8.9%	91.1%
Cartanyà-Hueso (2019)	Barcelona, Spain	Outdoor areas of schools	Should smoking be prohibited in outdoor areas of schools?	Yes (agree, totally agree); no (neutral, disagree, totally disagree)	n = 736	77.5%	77.5%
Gallus (2012)	Italy	School courtyards	Would you be in favour or against (the extension of) the smoking ban on school courtyards	Favour (moderately in favour, strongly in favour); oppose (moderately against, strongly against)	n = 6233	85.9%	
Kandra (2007)	North Carolina (USA)	Schools	To what degree do you support a tobacco free policy in your child's school so that no one, not students, nor teachers,	Strongly support; (moderately support, do not support)	n = 3973	85.6% (84.3 - 86.9)	

Author (year)	Geographic location	Smoke-free location	Support measure	Answer options	Overall sample size	Reported support overall	Support transformed, reversed etc.
			staff or visitors, could smoke or use other tobacco products on the school grounds at any time?				
Kruger (2015)	USA	School grounds	Should tobacco use be completely banned on school grounds, including fields and parking lots, and at all school events even for teachers and other adults?	Yes; no	n = 68489	86.1% (85.5 - 86.7)	
Martínez-Sánchez (2014)	Italy	School courtyards	Attitude towards smoke-free legislation in school courtyards	Support (moderately in favour, strongly in favour); no support (moderately against, strongly against)	n =6167	82.6%	
Nogueira (2022)	Bulgaria<br>England<br>France<br>Germany<br>Greece<br>Ireland<br>Italy<br>Latvia<br>Poland<br>Portugal<br>Romania<br>Spain	Outdoor areas of schools	For each of the following sites, are you:	Strongly in favour, moderately in favour, moderately against or strongly against a total tobacco ban?	Bulgaria: n = 1,050<br>England: n = 1,013<br>France: n = 1,018<br>Germany: n = 1,031<br>Greece: n = 1,000<br>Ireland: n = 941<br>Italy: n = 1,059<br>Latvia: n = 1,022<br>Poland: n = 724<br>Portugal: n = 1,000<br>Romania: n = 1,018<br>Spain: n = 1,026	Strongly in favour: 47.6%<br>Moderately in favour: 17.7%	

Author (year)	Geographic location	Smoke-free location	Support measure	Answer options	Overall sample size	Reported support overall	Support transformed, reversed etc.
Rosen (2012)	Israel	School yards	Support for smoke-free school yards	In favour (5-7); neutral (4); opposed (1-3)	n = 505	75.1%	
Sureda (2015)	Spain	Schools and high schools	To what extent do you agree or disagree with the prohibition of smoking in the following outdoor settings?	Agree (agree, totally agree); neither agree nor disagree; disagree (disagree, totally disagree)	n = 1302	70.5%	
Topuridze (2020)	Armenia, Republic of Georgia	Schoolyards	To what extent do you support or oppose a complete cigarette smoking ban in the following settings?	1: strongly oppose<br>2: somewhat oppose<br>3: don't know<br>4: somewhat support<br>5: strongly support	Armenia: n = 705<br>Georgia: n = 751	Mean (SD)<br>4.75 (0.78)	99.7%
Wiium (2009)	Norway	High-school premises	Total ban on smoking on high school premises	Totally agree, agree, neither agree nor disagree, disagree, totally disagree	n = 2381	Totally agree: 17.1 %<br>Agree: 37.9%	55.0%
**Outdoor non hospitality – event locations**							
Basto-Abreu (2016)	Baja California (Mexico)	Stadiums	Do you support the state level law that banned smoking in all outdoor areas where people gather for stadiums?	Yes; no	n = 796	80%	
Basto-Abreu (2016)	Baja California (Mexico)	Concert venues	Do you support the state level law that banned smoking in all outdoor areas where people gather for concert venues?	Yes; no	n = 796	83%	
Berg (2015)	Alabama, Florida, Georgia, Kentucky, North	Outdoor events like concerts, sporting events, and festivals	For each of the following places, indicate how you feel about a policy	Oppose (strongly oppose, somewhat oppose); other	n = 2501	29.1%	

Author (year)	Geographic location	Smoke-free location	Support measure	Answer options	Overall sample size	Reported support overall	Support transformed, reversed etc.
	Carolina, Mississippi, South Carolina, Tennessee (USA)		prohibiting smoking in that kind of place	(neutral, somewhat favour, strongly favour			
Berg (2016)	Republic of Georgia	Outdoor events like concerts, sporting events, festivals	How would you feel about a policy prohibiting smoking in that kind of place?	Oppose (somewhat oppose, strongly oppose); other (~~neutral,~~ somewhat favour, strongly favour)	n = 1163	33.9%	
Cartanyà-Hueso (2019)	Barcelona (Spain)	Outdoor sport locations	Should smoking be prohibited at outdoor sport locations?	Yes (agree, totally agree); no (neutral, disagree, totally disagree)	n = 736	65%	
Fong (2013)	France	Open-air concert	For open concert venues, smoking should be allowed in...?	All indoor areas; in some indoor areas; not allowed indoors at all	Wave 1 = 2250<br>Wave 2 = 2217<br>Wave 3 = 2196	Wave 1: 44.0% (38.7-49.5)<br>Wave 2: 27.4% (23.6-31,5)<br>Wave 3: 32.9% (28.0-38.2)	
Fong (2013)	France	Football stadium tribunes	For covered stands in football grounds, smoking should be allowed in...?	All indoor areas; in some indoor areas; not allowed indoors at all	Wave 1 = 2217<br>Wave 2 = 2210<br>Wave 3 = 2179	Wave 1: 54.5% (49.5-59.3)<br>Wave 2: 54.8% (50,5-58.9)<br>Wave 3: 57.5% (52.6-62.2)	

Author (year)	Geographic location	Smoke-free location	Support measure	Answer options	Overall sample size	Reported support overall	Support transformed, reversed etc.
Gallus (2012)	Italy	Sport stadiums	Would you be in favour or against (the extension of) the smoking ban in sport stadiums	Favour (moderately in favour, strongly in favour); oppose (moderately against, strongly against)	n = 6233	68.5%	
Li (2013)	New Zealand	Outside sports fields or courts	In your opinion, do you think people should be able to smoke at outside sport fields or courts?	Yes; no	n = 2672	59% (55 - 62)	
Martínez-Sánchez (2014)	Italy	Stadiums	Attitude towards smoke-free legislation in cars, in stadiums	Support (moderately in favour, strongly in favour); no support (moderately against, strongly against)	n = 6167	66.7%	
Nogueira (2022)	Bulgaria<br>England<br>France<br>Germany<br>Greece<br>Ireland<br>Italy<br>Latvia<br>Poland<br>Portugal<br>Romania<br>Spain	Stadium	For each of the following sites, are you:	Strongly in favour, moderately in favour, moderately against or strongly against a total tobacco ban?	Bulgaria: n = 1,050<br>England: n = 1,013<br>France: n = 1,018<br>Germany: n = 1,031<br>Greece: n = 1,000<br>Ireland: n = 941<br>Italy: n = 1,059<br>Latvia: n = 1,022<br>Poland: n = 724<br>Portugal: n = 1,000<br>Romania: n = 1,018<br>Spain: n = 1,026	Strongly in favour: 29.5%<br>Moderately in favour: 19.8%	49.3%
Rosenberg (2012)	Western Australia	Perth zoo	Do you support, do not support or have no feelings either way about smoke free policies in Perth Zoo?	Support; do not support; have no feelings either way	n = 2005	85%	

Author (year)	Geographic location	Smoke-free location	Support measure	Answer options	Overall sample size	Reported support overall	Support transformed, reversed etc.
Rosenberg (2012)	Western Australia	South Perth Foreshore Family zone (river front venue for music festivals)	Do you support, do not support or have no feelings either way about smoke free policies in South Perth Foreshore Family zone?	Support; do not support; have no feelings either way	n = 2005	82%	
Rosenberg (2012)	Western Australia	Perth Royal Show (annual agricultural event)	Do you support, do not support or have no feelings either way about smoke free policies at Perth Royal Show?	Support; do not support; have no feelings either way	n = 2005	80%	
Rosenberg (2012)	Western Australia	Adventure World Theme Park	Do you support, do not support or have no feelings either way about smoke free policies in Adventure World Theme Park?	Support; do not support; have no feelings either way	n = 2005	77%	
Rosenberg (2012)	Western Australia	Kings Park (national park located adjacent to the city centre)	Do you support, do not support or have no feelings either way about smoke free policies in Kings Park?	Support, do not support or have no feelings either way	n = 2005	52%	
Sureda (2015)	Spain	Sport centres	To what extent do you agree or disagree with the prohibition of smoking in the following outdoor settings?	Agree (agree, totally agree); neither agree nor disagree; disagree (disagree, totally disagree)	n = 1289	53.5%	
Walsh (2008)	New South Wales (Australia)	Sport stadia	Supporting government bans	Not reported	n = 1191	76.6% (74.2 - 79.0)	
Wiium (2009)	Norway	Outdoor sporting events	Total ban on smoking during outdoor sport events	Totally agree, agree, neither agree nor disagree, disagree, totally disagree	n = 2368	Totally agree: 18.8%<br>Agree: 39.0%	57.8%

Author (year)	Geographic location	Smoke-free location	Support measure	Answer options	Overall sample size	Reported support overall	Support transformed, reversed etc.
**Outdoor non hospitality – entrances**							
Al-Delaimy (2008)	California (USA)	Outside entrances to buildings	Just outside entrances to buildings	Please tell me if smoking should be allowed or not allowed in this place	n = 82,566	67.1% (65.2 – 69.0)	
Berg (2015)	Alabama, Florida, Georgia, Kentucky, North Carolina, Mississippi, South Carolina, Tennessee (USA)	Areas within 25 feet of an entrance to a public building	For each of the following places, indicate how you feel about a policy prohibiting smoking in that kind of place	Oppose (strongly oppose, somewhat oppose); other (neutral, somewhat favour, strongly favour	n = 2501	20.6%	79.4%
Berg (2016)	Republic of Georgia	Areas outside entrances to public places	How would you feel about a policy prohibiting smoking in that kind of place?	Oppose (somewhat oppose, strongly oppose); other (~~neutral,~~ somewhat favour, strongly favour)	n = 1163	26.0%	74.0%
Gendall (2013)	New Zealand	Outside building entrances and doorways	Indicate support for smoke-free outside building entrances and doorways	Eleven-point Likert scale	n = 828	65.0%	
Li (2016)	New Zealand	Public entrances	Smoking should be banned within 5 metres of the entrance of all buildings used by the public	Agree (Strongly agree, agree); not agree (neither agree nor disagree, disagree, strongly disagree)	n = 2,594	80.4%	
Lund (2016)	Norway	Entrances to all workplaces	What is your opinion if the government were to implement these regulations on smoking behaviour? Prohibit	Five-point Likert scale	n= 5543	Mean (SD) 3.79 (1.52)	69.7%

Author (year)	Geographic location	Smoke-free location	Support measure	Answer options	Overall sample size	Reported support overall	Support transformed, reversed etc.
			smoking at entrances to all workplaces				
Sæbø (2019)	Norway	Outdoor entrances to all workplaces	Ban smoking at outdoor entrances to all workplaces	Five-point Likert scale	n = 5543	53.3%	
Topuridze (2020)	Armenia, Republic of Georgia	Within 5m of public building entrances	To what extent do you support or oppose a complete cigarette smoking ban in the following settings?	1: strongly oppose<br>2: somewhat oppose<br>3: don't know<br>4: somewhat support<br>5: strongly support	Armenia: n = 705<br>Georgia: n = 751	Mean (SD) 3.33 (1.50)	58.2%
**Outdoor non hospitality – streets and open areas**							
Basto-Abreu (2016)	Baja California (Mexico)	Town squares	Do you support the state level law that banned smoking in all outdoor areas where people gather for town squares?	Yes; no	n = 796	84%	
Bo-Woo (2012)	Daejeon (Korea)	Tour sites	Should smoke-free zones be implemented at tour sites	Agree; disagree	n = 1013	79.6%	
Bo-Woo (2012)	Daejeon (Korea)	Pedestrian crosswalks	Should smoke-free zones be implemented at pedestrian cross-walks	Agree; disagree	n = 1013	80.1%	
Bo-Woo (2012)	Daejeon (Korea)	Sidewalks	Should smoke-free zones be implemented at sidewalks	Agree; disagree	n = 1013	76.7%	
Bo-Woo (2012)	Daejeon (Korea)	Parking lots	Should smoke-free zones be implemented in parking lots	Agree; disagree	n = 1013	79.6%	
Cartanyà-Hueso (2019)	Barcelona (Spain)	Outdoor areas shopping centres	Should smoking be prohibited in outdoor shopping centres?	Yes (agree, totally agree); no (neutral,	n = 736	42.5%	

Author (year)	Geographic location	Smoke-free location	Support measure	Answer options	Overall sample size	Reported support overall	Support transformed, reversed etc.
				disagree, totally disagree)			
Gendall (2013)	New Zealand	Footpaths in shopping areas	Indicate support for smoke-free footpaths in shopping areas	Eleven-point Likert scale	n = 828	61.0%	
Rashid (2014)	George Town (Malaysia)	City centre	Support for making the GTWHS a smoke free zone	Support; no support	n = 2969	90.9%	
eRhoades (2019)	Oklahoma (USA)	City owned properties	Policies that prohibit tobacco use on city-owned properties	Favour; neither favour nor oppose; oppose	n = 4461	75.3%	
Sureda (2015)	Spain	Shopping centres	To what extent do you agree or disagree with the prohibition of smoking in the following outdoor settings?	Agree (agree, totally agree); neither agree nor disagree; disagree (disagree, totally disagree)	n = 1298	38.4%	
**Outdoor non hospitality – health care**							
Cartanyà-Hueso (2019)	Barcelona (Spain)	Outdoor areas of hospitals and health centres	Should smoking be prohibited in outdoor areas of hospitals and health centres	Yes (agree, totally agree); no (neutral, disagree, totally disagree)	n = 736	77.6%	
Clegg (2018)	Greater Manchester, UK	Hospital	Level of agreement that patients and visitors/staff should not smoke on the hospital grounds	5 point Likert scale, strongly agree to strongly disagree	n = 477	Patients/visitors shouldn't smoke: 61%<br>Agreed staff shouldn't smoke: 67%	64.1%
Crosby (2018)	Wakefield, UK	Hospital entrances	Hospital patients/staff/visitors should not smoke at hospital building entrances	Agree (strongly agree, agree); not agree (neither agree nor disagree,	n = 459	Patients: 91%<br>Staff: 92%<br>Visitors: 92%	91.0%

Author (year)	Geographic location	Smoke-free location	Support measure	Answer options	Overall sample size	Reported support overall	Support transformed, reversed etc.
				disagree, strongly disagree)			
Gallus (2012)	Italy	Outdoor areas surrounding hospitals	Would you be in favour or against (the extension of) the smoking ban in outdoor areas surrounding hospitals	Favour (moderately in favour, strongly in favour); oppose (moderately against, strongly against)	n = 6233	79.9%	
Hale (2017)	Australia	Health care grounds	What is your attitude towards Peninsula Health's Smoke Free policy?	Positive (positive, very positive); negative (neutral, negative, very negative)	2011: n = 873<br>2013: n = 1108	2011: 70%<br>2013: 74%	
Lin (2006)	Duarte, California (USA)	Hospital	Not reported (*link in paper to original survey not working)*	Policy was a good idea; not reported	n = 1356	92.6%	
Martínez-Sánchez (2014)	Italy	Open areas of hospitals	Attitude towards smoke-free legislation in open areas of hospitals	Support (moderately in favour, strongly in favour); no support (moderately against, strongly against)	n = 6167	75.0%	
Nogueira (2022)	Bulgaria<br>England<br>France<br>Germany<br>Greece<br>Ireland<br>Italy<br>Latvia<br>Poland<br>Portugal	Outdoor areas of hospitals	For each of the following sites, are you:	Strongly in favour, moderately in favour, moderately against or strongly against a total tobacco ban?	Bulgaria: n = 1,050<br>England: n = 1,013<br>France: n = 1,018<br>Germany: n = 1,031<br>Greece: n = 1,000<br>Ireland: n = 941<br>Italy: n = 1,059<br>Latvia: n = 1,022<br>Poland: n = 724<br>Portugal: n = 1,000	Strongly in favour: 35.5%<br>Moderately in favour: 19.4%	54.9%

Author (year)	Geographic location	Smoke-free location	Support measure	Answer options	Overall sample size	Reported support overall	Support transformed, reversed etc.
	Romania<br>Spain				Romania: n = 1,018<br>Spain: n = 1,026		
Riad-Allen (2017)	Toronto (Canada)	Hospital campus (Baseline)	I support the creation and implementation of a tobacco free policy at CAMH	Five-point Likert scale	Staff: n = 454<br>Patient: n = 123	Mean (SD)<br>Staff: 3.89 (1.31)<br>Patient 3.15 (1.39)	68.5%
Riad-Allen (2017)	Toronto (Canada)	Hospital campus (soft launch)	I support the creation and implementation of a tobacco free policy at CAMH	Five-point Likert scale	Staff: n=356,<br>Patient: n = 106	Staff: 3.75 (1.39)<br>Patient: 3.50 (1.50)	67.3%
Riad-Allen (2017)	Toronto (Canada)	hospital campus (hard launch)	I support the creation and implementation of a tobacco free policy at CAMH	Five-point Likert scale	Staff: n=363<br>Patient: n = 193	Staff: 3.87 (1.34)<br>Patient:3.42 (1.38)	67.8%
Rosen (2012)	Israel	Entrances to health care facilities	Support for smoke-free entrances to health care facilities	In favour (5-7); neutral (4); opposed (1-3)	n = 505	92.6%	
Sureda (2015)	Spain	Healthcare centres	To what extent do you agree or disagree with the prohibition of smoking in the following outdoor settings?	Agree (agree, totally agree); neither agree nor disagree; disagree (disagree, totally disagree)	n = 1301	52.7%	
Unrod (2012)	Tampa, Florida (USA)	Hospital campus	Support to the tobacco-free campus (pre-ban)	Not reported	Employees Pre-ban: n = 607<br>Employees Post-ban: n = 511	See subgroup smoking status	Pre-ban: 78.3%<br>Post-ban: 82.0%
Wheeler (2007)	Little Rock, AR (USA)	Hospital campus	Support for smoke-free policy (after implementation)	Not reported	n = 842	83.3%	

Author (year)	Geographic location	Smoke-free location	Support measure	Answer options	Overall sample size	Reported support overall	Support transformed, reversed etc.
Wheeler (2007)	Little Rock, AR (USA)	Hospital campus	Support for smoke-free policy (before implementation)	Not reported	n = 912	89.9%	
Wheeler (2007)	Little Rock, AR (USA)	Hospital campus	Support for smoke-free policy (patient sample)	Not reported	n = 183	87.7%	
**Outdoor non hospitality – other**							
Basto-Abreu (2016)	Baja California (Mexico)	All public places	Do you support the state level law that banned smoking in all outdoor areas where people gather?	Yes; no	n = 796	72%	
Bo-Woo (2012)	Daejeon (Korea)	Protection zones for children (School zones)	Should smoke-free zones be implemented at protection zones for children	Agree; disagree	n = 1013	98.5%	
Bo-Woo (2012)	Daejeon (Korea)	Residential areas	Should smoke-free zones be implemented at residential areas	Agree; disagree	n = 1013	74.3%	
Gendall (2013)	New Zealand	Outdoor areas combined		Eleven-point Likert scale	n = 828	6.5 out of 10	65.0%
Kandra (2013)	North Carolina (USA)	Outdoor public places	Exposure to second-hand smoke in outdoor public places should:	Not be allowed; be allowed at some times or in some places; be allowed anywhere	n = 3301	63.1 % (61.0 - 65.3)	
King (2014)	USA	Outdoor workplaces	At workplaces, do you think smoking outdoors should be:	Never allowed; only allowed at some places; always allowed	n = 65,120	23.2%	
Klein (2007)	Minnesota (USA)	Outdoor areas kids	Smoking should be prohibited in outdoor areas used by kids	Strongly agree; somewhat agree; somewhat disagree; strongly disagree	n = 1493	Strongly agree: 57% Somewhat agree: 21%	78%

Author (year)	Geographic location	Smoke-free location	Support measure	Answer options	Overall sample size	Reported support overall	Support transformed, reversed etc.
Li (2013)	New Zealand	Outside areas where children are likely to go	Smoking should be banned in all outdoor public places where children are likely to go	Strongly agree; agree; neither agreed nor disagreed; disagreed; strongly disagreed	n = 2672	Strongly agree: 40% (37 - 43) Agree: 33% (31 - 36)	73%
Li (2016)	New Zealand	Outside areas where children are likely to go	Smoking should be banned in all outdoor public places where children are likely to go	Agree (strongly agree, agree); not agree (neither agreed nor disagreed, disagreed, strongly disagreed)	n = 2,594	83.7%	
Topuridze (2020)	Armenia, Republic of Georgia	Other public outdoor areas	To what extent do you support or oppose a complete cigarette smoking ban in the following settings?	1: strongly oppose<br>2: somewhat oppose<br>3: don't know<br>4: somewhat support<br>5: strongly support	Armenia: n = 705<br>Georgia: n = 751	Mean (SD) 3.41 (1.56)	60.3%
**Outdoor hospitality**							
Al-Delaimy (2008)	California (USA)	Outdoor dining areas	Outdoor restaurants dining patios	Please tell me if smoking should be allowed or not allowed in this place	n = 82,566	70.0% (68.8 – 71.7)	
Berg (2015)	Alabama, Florida, Georgia, Kentucky, North Carolina, Mississippi, South Carolina, Tennessee USA	Outdoor seating areas of bars and restaurants	For each of the following places, indicate how you feel about a policy prohibiting smoking in that kind of place	Oppose (strongly oppose, somewhat oppose); other (neutral, somewhat favour, strongly favour	n = 2501	15.8%	69.5%

Author (year)	Geographic location	Smoke-free location	Support measure	Answer options	Overall sample size	Reported support overall	Support transformed, reversed etc.
Berg (2016)	Republic of Georgia	Outdoor seating areas of restaurants, bars, pubs, clubs	How would you feel about a policy prohibiting smoking in that kind of place?	Oppose (somewhat oppose, strongly oppose); other (~~neutral,~~ somewhat favour, strongly favour)	n = 1163	40.9%	59.1%
Fong (2013)	France	Outdoor areas of restaurants	Do you think that smoking should be allowed in...?	Not allowed at all; in some areas; all areas	Wave 1 = NA<br>Wave 2 = 2219<br>Wave 3 = 2202	Wave 1: NA<br>Wave 2: 32.8% (28.7 - 37.2)<br>Wave 3: 36.2% (31.6 - 41.1)	
Kennedy (2012)	France	Outdoor areas of restaurants	Do you think that smoking should be:	Not allowed in outdoor eating areas at all; in designated outdoor eating areas such as smoking terraces; allowed in all outdoor eating areas	n = 1654	See smoking status subgroup	14.3%
Li (2016)	New Zealand	Outdoor dining areas	Smoking should be banned in all public outdoor dining areas	Agree (strongly agree, agree); not agree (neither agreed nor disagreed, disagreed, strongly disagreed)	n = 2,594	75.9%	
Lund (2016)	Norway	Outdoor seating areas in restaurants	What is your opinion if the government were to implement these regulations on smoking behaviour? Prohibit	Five-point Likert scale	n = 5543	Mean (SD) 3.23 (1.66)	55.7%

Author (year)	Geographic location	Smoke-free location	Support measure	Answer options	Overall sample size	Reported support overall	Support transformed, reversed etc.
			smoking at outdoor seating areas in restaurants				
Lund (2016)	Norway	Outdoor seating areas in bars	What is your opinion if the government were to implement these regulations on smoking behaviour? Prohibit smoking at outdoor seating areas in bars	Five-point Likert scale	n = 5543	3.15 (1.67)	63.0%
Nogueira (2022)	Bulgaria<br>England<br>France<br>Germany<br>Greece<br>Ireland<br>Italy<br>Latvia<br>Poland<br>Portugal<br>Romania<br>Spain	Restaurants and bars patios	For each of the following sites, are you:	Strongly in favour, moderately in favour, moderately against or strongly against a total tobacco ban?	Bulgaria: n = 1,050<br>England: n = 1,013<br>France: n = 1,018<br>Germany: n = 1,031<br>Greece: n = 1,000<br>Ireland: n = 941<br>Italy: n = 1,059<br>Latvia: n = 1,022<br>Poland: n = 724<br>Portugal: n = 1,000<br>Romania: n = 1,018<br>Spain: n = 1,026	Strongly in favour: 27.5%<br>Moderately in favour: 19.3%	46.8%
Reuband (2014)	Germany	Terraces	Should smoking be ... at terraces?	Completely forbidden; partially forbidden; not forbidden	n = 1046	16.0%	
Rodríguez-González (2021)	Nuevo Leon (Mexico)	Terraces	The law must prohibit smoking on terraces even if they do not have walls	Agree, disagree, indistinct, does not know	n = 1070	76.8%	

Author (year)	Geographic location	Smoke-free location	Support measure	Answer options	Overall sample size	Reported support overall	Support transformed, reversed etc.
Topuridze (2020)	Armenia, Republic of Georgia	Terrace	To what extent do you support or oppose a complete cigarette smoking ban in the following settings?	1: strongly oppose<br>2: somewhat oppose<br>3: don't know<br>4: somewhat support<br>5: strongly support	Armenia: n = 705<br>Georgia: n = 751	2.88 (1.60)	47.0%
Walsh (2008)	New South Wales (Australia)	Outdoor dining areas	Supporting government bans	Not reported	n = 1191	68.9% (66.3 - 71.5)	

Appendix IV – Overview policies and samples

Author (year)	Description of policy	Implementation date	Implementation level	Enforcement	Observational period	Selection of participants
Al-Delaimy (2008)	Support for hypothetical policies	-	-	-	2005	California Tobacco Survey 2005. Two-stage sampling procedure and random digit dialling of Californian adults.
Abundis (2008)	Support for hypothetical policies	-	-	-	February 2008	Systematic random sampling with probability proportional to size
Agaku (2014)	Support for hypothetical policies	-	-	-	May 2010 to January 2011	Multistage stratified area probability sample of households to reflect the civilian non institutionalized US adult population
Almutairi (2014)	Support for hypothetical policies	-	-	-	Academic year 2013	Randomly selected staff and faculty members as well students.
Atiba (2020)	State-wide smoke free policy but university campuses were excluded from the list of public places where smoking would be disallowed	January 2014	State level (Lagos State, Nigeria)	Not reported	2015	Multistage sampling method, two largest universities selected, three faculties selected and two random departments within each faculty. Four academic levels in each department selected to recruit required number of students
Bartington (2020)	Support for hypothetical policies	-	-	-	May 2016 to April 2017	Staff, undergrad/postgrad students, enrolled at Edgbaston Campus during observation period.
Basto-Abreu (2016)	Legislation banning outdoor smoking in public gathering areas such as parks, stadiums and beaches	September 2013	Not reported	Not reported	October 2013 to March 2014	Multistage stratified cluster sampling, in 5 cities in Baja California State. Census tracts and street blocks

Berg (2015)	Support for hypothetical policies	-	-	-	June-July 2013	Eligible participants were individuals from the GMI panel living in the USA, English speaking, and 18-65 years old. Daily e-mail invitations and targeted email invitations to panellists known to meet some of the study criteria
Berg (2016)	Support for hypothetical policies	-	-	-	February - May 2014	Adults age 18-64, multi-stage clustered sample design based on most recent census data. Random walk method to select households from 122 clusters in 22 strata. Households with two eligible people used KISH method.
Berg (2020)	Campus-wide smoking ban	2014	Local level, campus wide	Not reported	Pre ban 2014 and post ban 2018	Email sent to all students and paper surveys handed out at student activity centre
Boeckmann (2018)	Support for five possible legislative changes in Germany	-	-	-	September 2016	National representative sample age 14 and older, multi-stage, multi-stratified random probability sampling approach
Bo-Woo (2012)	South-Korea National Health promotion act in 2010, making municipalities enforce non-smoking areas	2010, by 2012 85 out of 244 governments implemented smoke-free zones	Local governments	Fines up to 100.000 won	June 13 to 14 2011	Adult residents (older than 19 years) living in Daejeon Metropolitan Area. Proportional allocation method by region, age and sex.
Bower (2005)	Support for hypothetical policies	-	-	-	Assessment day 2003	Freshman and juniors who participated during Assessment Day (i.e. mandatory first day of academic year)

Braverman (2015)	Smoke-free campus policy at a public university in Pacific Northwest, U.S.	2012	Local level	Not reported	May 2013	Email invites send to all students registered for an on-campus course of one credit or more and staff/faculty who worked at campus at least 20% time during spring 2013<br>Email addresses obtained from campus administrative offices
Braverman (2021)	Campus for which a smoke-free policy was in effect	September, 2012	Local level, campus wide	Not reported	May-June 2013 and October-November 2018	Email sent to all students and reminder emails sent to those that did not respond
Brown (2019)	Smoke-free prison policy	November 2018	National	Not reported	November 1st to December 16 2016	All prison staff and prisoners in Scotland were invited.
Burns (2013)	Smoke-free campus policy at Curtin University, Australia	January 1st 2012	Local	Not reported	October 2011	Random cross-sectional sample of staff and students from all faculties
Buth (2013)	Development of non-smoker protection in prisons, total smoking ban in prisons including cells	Not yet implemented	Not reported	Not reported	September - December 2011	Surveys were handed out to all prisoners in 15 participating prisons.
Cartanyà-Hueso (2019)	Law 42/2010; extended smoke-free regulation to all hospitality venues without exception and to some outdoor areas, including hospital premises, educational campuses, and playgrounds	2011	National law	Not reported	May 2013 - February 2014	All participants from baseline survey with vital status and contact information who provide informed consent to participate. National representative sample.

Chaaya (2021)	Campus tobacco-free policy	January, 2018	Local level, campus wide	Not reported	Pre and post ban November, 2017 and November 2018	Random selection of courses by stratified cluster sampling, professors of selected courses asked for permission. For approved courses researches visited and asked students present to complete survey
Clegg (2018)	Hypothetical	-	-	-	October 2019 to May 2020	Survey was distributed via repeated internal email communications containing a link to the online survey. The survey was also advertised via trust screensavers with QR codes and distributed flyers in staff rooms with QR codes to allow access to the survey via a smartphone or tablet
Crosby (2018)	Hypothetical support before and after a social norm campaign	-	-	-	Before: 10–18 September 2012 After: 17 – 21 December 2012.	All hospital patients, visitors, and staff on the premises eligible, 1,000 individuals approached
Díez-Izquierdo (2017)	Law 42/2010; extended smoke-free regulation to all hospitality venues without exception and to some outdoor areas, including hospital premises, educational campuses, and playgrounds	2011	National law	Not reported	March to April 2016	Participants from ONMIBUS survey, national representatives of Spanish adult population (16-75 years).

Dono (2014)	Tobacco Products Regulation Act 2007 smoking bans in prescribed (outdoor) public transport areas (bus stops, tram stops, railway stations, taxi ranks and airports) and within 10 m of children's outdoor playground equipment	May 31st 2012	State level	Maximum penalty 200$	March-April 2011 (wave 1; pre-legislation), April-June 2012 (wave 2; pre-legislation) and October–December 2013 (wave 3; post-legislation)	For each wave, a random sample of telephone numbers was sourced from the Electronic White Pages, selecting adults (18+)
Drach (2010)	Smoke-free policy for all indoor and outdoor communal areas within 25 feet of buildings	January 1st 2008	LCC, largest property management company in Portland, Oregon	Not reported	Questionnaire in May 2008, follow-up interviews in August 2008 and 2009	839 tenants from 17 subsidized buildings received questionnaires
Dunn (2008)	Support for hypothetical policies	-	-	-	March 2006	A probability sample of Queensland adults was generated using random digit dialling methods. Quota sampling was undertaken to ensure a 50% gender division and an equal division between the Brisbane metropolitan region and the rest of the state.
El Ansari (2012a)	Support for hypothetical policies	-	-	-	Academic year 2009-2010	Representative random sample of students at eleven participating faculties at Assiut University.
El Ansari (2012b)	Support for hypothetical policies	-	-	-	Academic year 2007-2008	Data collected as part of the General Student Health Survey

El Ansari (2021)	Support for hypothetical policies	-	-	-	2013-14 academic year	Email invites to all undergrads at all faculties
Fallin (2015)	Support for hypothetical policies	-	-	-	September 2013 to May 2014	Students at 8 Californian colleges and universities, recruited from high-traffic areas on campus, approached by the intercept method.
Farran (2021)	Tobacco free policy	January, 2018	Local level, campus wide	Not reported	December, 2017 and December 2018	A sample of 1722 staff and 960 faculty members was randomly selected in each round. Faculty and staff were sent invitations by emails and were asked to access a survey link if they considered participating. To ensure participation of lower grade staff (who rarely use their email accounts), hard copies of the questionnaire were also placed for two weeks in the administration offices of almost all departments
Fong (2013)	Implementation of a comprehensive smoke-free policy in public places in France in 2007, extended to public meeting places in 2008	February 2007, extended January 2008	National	Not reported	December 2006 to February 2007 (wave 1), September to November 2008 (wave 2) and September to December 2012 (wave 3)	ITC France project. Adults age 18 and over. Random digit dialling sampling design, no stratification. Recruitment until planned sample was reached. Next Birthday method was used.
Gallus (2012)	Possibility to extend smoking ban to selected outdoor areas in Italy	Not yet implemented	-	-	March to April 2009 and 2010	Italian adults age 15 and over, representative in age, sex, geographic area and socio-economic characteristics.
Garg (2011)	Completely smoke-free campus declaration	July 2011	Not reported	Not reported	November 11 2009	All student residents were invited to participate.

Gendall (2013)	Support for hypothetical policies	-	-	-	March, 2012	Smokers and non-smokers selected from Research Now panel, which can be nationally representative but required equal numbers of smokers and non-smokers in sample to enhance comparison.
Gentzke (2018)	Support for hypothetical policies	-	-	-	August to December, 2012	Aged 18 and older who resided in multi-unit housing and lived in the specified communities. Random digit dialling of landlines (18.000) and cell phones (60.000)
Gillespi (2005)	Support for hypothetical policies	-	-	-	2004	General population sample, current smoker and recent quitter sample and a Maori sample. Fieldwork carried out by TNS New Zealand (market research company).
Hale (2017)	Smoke-free policy at Peninsula Health	September 2010	Not reported	Not reported	March 2010 (pre-implementation), March 2011 and September 2013 (post-implementation)	Email invites send to all staff and hard copy questionnaires added to payslips. Per time point four weeks of data collection.
Hammond (2006)	Support for hypothetical policies	-	-	-	November 1 to December 10, 2003	Convenience sample selected from central locations at campus.
Hewett (2012)	Support for hypothetical policies	-	-	-	January 6 to March 6, 2009	Community owner-occupants in the USA, samples drawn from two non-overlapping lists maintained by InfoUSA.com on multi and single family unit housing
Ickes (2017)	Smoke-free campus policy at University of Kentucky, U.S.	November 2009	Local level	Not reported	April, 2013	Random selection of students aged 18 or older with official university email address.

Jalleh (2006)	Support for hypothetical policies	-	-	-	Not reported	Participants age 25-54 who participated in surveys evaluating the impact of anti-tobacco campaigns
Johnston Polacek (2008)	Support for hypothetical policies	-	-	-	October 2004 to March 2005	Classrooms and public spaces served as data-gathering sites combined with survey on university website
Kandra (2007)	Support for hypothetical policies	-	-	-	2005	2005 Child Health Assessment and Monitoring Program (CHAMP) survey; a random telephone survey of noninstitutionalized adults age 18 and older.
Kandra (2013)	Support for hypothetical policies	-	-	-	2009	2009 North Carolina Youth Tobacco Survey (NCYTS)
Karadağ (2021)	Hypothetical support	-	-	-	June 2019 and September 2019	Online questionnaire sent to 12,000 university students
Kecojevic (2020)	Hypothetical support	-	-	-	December, 2017	One-time email invitation through HR and student registers
Kennedy (2012)	Support for hypothetical policies	-	-	-	December 2006 to February 2007 (wave 1) and September to November 2008 (wave 2)	ITC France project. Adults age 18 and over. Random digit dialling sampling design, no stratification. Recruitment until planned sample was reached. Next Birthday method was used.
Kennedy (2015)	Smoke-free policy for all new leases and transfers in regionally-owned community housing, Waterloo region, Canada	April 1, 2010	Local level	Not reported	March 2010 (pre-implementation), March 2011 and March 2013 (post-implementation	Printed surveys distributed at tenants

King (2010)	Support for hypothetical policies	-	-	-	May 2007 to May 2009	New York State Adult Tobacco Survey (NYATS) participants who reported living a multi-unit housing complex.
King (2014)	Support for hypothetical policies	-	-	-	2009-2010	National Adult Tobacco Survey (NATS); stratified telephone survey among noninstitutionalized US adults age 18+ through landline and cellular
Klein (2007)	Support for tobacco-free parks in Minnesota, U.S., where policy is already implemented	Not reported	Local level	Not reported	Late summer 2004	Random sample selected from driving licence records, living close to a tobacco-free park based on the city or county of residence on licence.
Kruger (2015)	Support for hypothetical policies	-	-	-	October 20, 2009 to February 28, 2010	National Adult Tobacco Survey (NATS); stratified telephone survey among noninstitutionalized US adults age 18+ through landline and cellular
Lechner (2012)	Prohibition of use, sale and promotion of tobacco on university properties in the U.S.	July 2008	Local level	Not reported	Fall 2007 (baseline), 2008, 2009 and 2010	Cluster randomized sample from office of registrar lists.
Li (2013)	Support for hypothetical policies	-	-	-	2012	2012 Health and Lifestyles Survey (HLS)
Li (2016)	Support for extending smoke-free areas	Not yet implemented	Not reported	Not reported	2014	2014 Health and Lifestyles Survey (HLS)
Licht (2012)	Support for hypothetical policies	-	-	-	January to March (landline) and October to December (cell phone), 2010	Two nationally representative random-digit-dial samples of US adults living in multi-unit housing.

Lin (2006)	Smoke-free City of Hope (California, U.S.) campus policy, prohibiting smoking anywhere on campus grounds both inside and outside of buildings	1989	Local level	Not reported	Not reported	Emails send to all City of Hope employees and posts in campus publications.
Loukas (2006)	Support for hypothetical policies	Not reported	Not reported	Not reported	Spring 2003	Convenience sample selected from 5 participating colleges, surveys distributed during class time
Lugo (2017)	Legislation in Italy extending smoking ban to selected outdoor areas and to private cars in the presence of children or pregnant women	2016	National law	Not reported	February to April 2015 and December 2015 to February 2016	Representative multistage sampling, Italian adult population
Lund (2016)	Government proposals for regulating smoking behaviour	Not yet implemented	Not reported	Not reported	December 2014	Pool of registered internet panellists from market research firm (Ipsos MMI) and mobile phone lists. Quotas set by gender, age and geography
Mamudu (2016)	Tobacco free policy at East Tennessee State University, allowing smoking only in private vehicles.	August 2008	Local level	Not reported	April to May 2011	Anonymous internet based survey, selecting self-reported smokers
Marsh (2014)	Support for hypothetical policies	-	-	-	October 2012	Random sample of students and staff selected from University of Otago HR and student enrolment databases
Martínez-Sánchez (2014)	Support for hypothetical policies	-	-	-	February to April 2011 and February to May 2012	Face-to-face interviews conducted by DOXA

McMillen (2004)	Support for hypothetical policies	-	-	-	July to September, 2002	The Social Climate Survey of Tobacco Control; random digit dialling adults age 18 and older
McMillen (2019)	Policy prohibiting the use of combustible tobacco products in all public housing living units, indoor common areas and Public Housing Authorities administrative office buildings, as well as all outdoor areas up to 25ft from public housing in the U.S.	July 31, 2018	National law	Not reported	2015	2015 Social Climate Survey of Tobacco Control; dual-frame survey administered to national probability samples of U.S. adults
Meng (2016)	Support for hypothetical policies	-	-	-	October 2015 to January 2016	Adults age 18 years and older living in multi-unit housing in target districts in the city of Los Angeles. Those living in residential motels/hotels or single-family homes were excluded.
Mishra (2011)	Support for hypothetical policies	-	-	-	Spring 2009	All faculty, staff and students registered on the university's e-mail network were emailed with an incentive to complete survey, there was 3 follow up emails to non-respondents
Morain (2013)	Support for hypothetical policies	-	-	-	October 12 to 24, 2011	KnowledgePanel; standing, probability based, nationally representative sample of US adults. Panel is recruited through random digit dialling and address-based sampling
Niemeier (2014)	Support for hypothetical policies	-	-	-	January, 2012	Students age 18 and older, invited through e-mail and web sites
Nogueira (2022)	Support for hypothetical policies	-	-	-	2017-2018	TackSHS Survey, cross sectional survey with representative samples from the

						general population of 12 European countries. Subjects are age 15 or over and representative for age, sex, habitat, and socio-economic characteristics. Multistage, cluster or stratified random sampling methods used. Face-to-face computer assisted interviews.
Okoli (2013)	Smoke-free bylaw banning smoking of any substance in the city's park, beaches and recreational facilities in Vancouver, Canada	September 1, 2010	Local level	Not reported	September 15 to 25, 2011	Random digit dialling Vancouver residents, performed by survey research company NRG Research Group
Patel (2022)	By 2018 all public housing agencies are required to implement smoke-free policies	2018	Country level	Not reported	October-November 2018	Ipsos KnowledgePanel, nationally representative sample of English and Spanish speaking adults 18-65, address and probability weighted
Pederson (2014)	Smoke-free bylaw banning smoking of any substance in the city's park, beaches and recreational facilities in Vancouver, Canada	September 1, 2010	Local level	Not reported	September 15 to 25, 2011	Random digit dialling Vancouver residents, performed by survey research company NRG Research Group
Ramachandran (2020)	Smoke-free policy at the University of Mississippi, including all indoor and outdoor grounds including residence halls and private vehicles	August 1st 2012	Local level	Not reported	Fall semester 2015	Sampling frame based on undergraduate classes in the fall of 2015, short survey provide in a random sample of classes
Rashid (2014)	Initiative to make Penang, Malaysia a smokes free state, starting with Georg Town World Heritage Site	2011	Local level	Not reported	Middle 2012 to April 2013	Multistage sampling process at multiple premises (e.g. eating outlets, accommodations, places of worship etc.)

Reuband (2014)	Support for hypothetical policies	-	-	-	2009	Random sample from Dusseldorf city population register
Rhoades (2019)	Proposed policy that local municipalities in Oklahoma can enact to make their own properties, indoor and outdoor, tobacco-free	Not yet implemented	Not reported	Not reported	2016	Stratified sampling strategy to equally represent each 6 regions in Oklahoma, landline and telephone interviews
Riad-Allen (2017)	Centre for Addiction and Mental Health (CAMH) tobacco-free campus policy	April 30, 2014	Local level	Not reported	March 1 to April 29, 2014 (pre-implementation), September 1 to November 6, 2014 (soft launch) and march 1 to May 1, 2015 (post-implementation)	Staff surveys sent through public affairs office, patient surveys distributed through their clinical teams or volunteers
Rodríguez-González (2021)	Support for hypothetical policies	-	-	-	Not reported	Adults age 18 and over selected from the white section of the 2015 telephone directory, computer assisted telephone interviews. Excluding those who acknowledged using drugs, alcohol or psychotics in the last 12 hours.
Rosen (2012)	Support for hypothetical policies	-	-	-	November 30 to December 22, 2010	National phone survey among Israeli adults

Rosenberg (2012)	Support for hypothetical policies	-	-	-	November 2010	Western Australia adults age 18-69, randomly selected through electronic household directories
Ruokolainen (2018)	Tobacco end game aim to end tobacco use in Finland by 2030	2010, updated in 2016	Not reported	Not reported	2012	FINRISK study data; Adults age 25-74, stratified random sample drawn from population register. Postal invitation for examination and questionnaire, questionnaire on smoking given after examination and returned by mail
Sabrian (2019)	Support for hypothetical policies	-	-	-	July to October, 2016	Students of Universities Riau, They were conveniently recruited from all schools and faculties in the university.
Sæbø (2019)	Support for the proposal to ban smoking in vehicles when children are present	Proposed in 2012, not implemented yet	Not reported	Not reported	December 2014 to January 2015	IPSOS MMI web panel; double opt-in method combined with info on background variables resulting in a random sample of individuals who agreed to participate in surveys and who are active members of the panel. Mobile platform following population extraction in the 15-30 age group. Sampling quotas for age, gender and region
Schmidt (2016)	Support for hypothetical policies	-	-	-	October to December 2013	Adult tenants living in Public Housing Authority buildings in Montana
Sendall (2021)	Prohibition of smoking in all areas of the university, including the grounds, buildings and vehicles	July 2016	Not reported	Not reported	September-October 2016	Invitation to participate sent through the register office newsletter
Seo (2005)	Support for hypothetical policies	-	-	-	October 16 to November 16, 2003	Computer assisted telephone interviews using random digit dialling among adults in Indiana, U.S.

Stevenson (2008)	Smoke-free park policy in Upper Hutt, New Zealand	May 2006	Local	Signage and media coverage rather than bylaws	September, 2007	Face-to-face interviews among park users
Stillman (2018)	Support for hypothetical policies	-	-	-	July 2014, January 2015 and May 2015	Tobacco Use Supplement to the Current Population Survey (TUS-CPS); U.S. adults age 18 and older selected based on the 2000 Census.
Sureda (2015)	Smoke-free law (Law 42/2010) prohibiting smoking in some outdoor areas (e.g. hospital premises, school courtyards and children's playgrounds)	January 2, 2011	National	Not reported	June 2011 to March 2012	Representative random sample of the official 2010 population census of Barcelona. Sample size set at 1560 (α error of 5%, β error of 20%, and 20% losses for independent samples). Participants that declined or could not be located where replaced at random in the same strata.
Sweeting (2021)	Complete prison smoking ban	November, 2018	National level	Not reported	Phase 1: November to December 2016<br>Phase 2: May to July 2018<br>Phase 3: May to July 2019	Staff distributed and collected paper questionnaires in all 15 Scottish prisons
Thompson (2006)	Support for hypothetical policies	-	-	-	Not reported	Sample of students in 30 participating schools, oversampling freshmen (~750) and approximately 200 students in each of the remaining classes (i.e., sophomores, juniors, and seniors). For the 16 schools with less than 1,350 students, we surveyed all students.

Topuridze (2020)	Support for hypothetical policies	-	-	-	October-November 2018	Armenia: target of 50 households per city, 1128 households visited of which 819 were eligible and 705 participated<br>Georgia: multistage cluster sampling, 5 clusters per city. 985 households visited, 910 eligible and 751 participated
Unrod (2012)	Campus wide outdoor smoking ban at Moffitt Cancer Centre (MCC)	April, 2008	Local	Not reported	January 2008 (pre-ban) and July 2008 (post-ban)	All employees at MCC, subset of patients in which inpatients were oversampled because they would be impacted by the smoking ban to a greater degree compared with outpatients
Waddell (2014)	Smoke-free air rules in public pars and on beaches, extension of the Smoke-Free Air Act of 2002	2010	Local	Not reported	August 2010, April 2011 and February 2012	Citywide landline and cellular phone survey, participants requited through phone number.
Wallar (2013)	Support for hypothetical policies	-	-	-	November 19 to 30, 2012	In person recruitment at neutral, high student volume places on campus
Walsh (2008)	Support for hypothetical policies	-	-	-	October to December 2004	New Zealand households selected through New South Wales Electronic White Pages, quotas were applied to increase the representativeness of the sample and data were weighted to the NSW population. Letters were sent to 12,000 addresses of which 4,859 (40.5%) proved to be businesses or were ineligible for other reasons.

Wang (2018)	US Department of Housing and Urban development rule requiring US public housing agencies (PHA) to implement a smoke free policy	February 3, 2017	National	Law required PHA to have policies, enforcement is up to PHA	2016 (18 months prior to rule)	US adults age 18 and older, random sampling through address based probability sampling. Data are weighted to be nationally representative and match US current population survey proportions of nine demographic variables
Wheeler (2007)	University of Ankara for Medical Sciences (UAMS) smoke-free campus policy	July 4, 2004	Local	Compliance would be the responsibility of administrators with security personnel as back up	April 2004 and May 2005	The personnel roster from human resources was used to randomly sample 1,400 from approximately 9,000 employees without replacement.
Wiliams (2011)	Support for hypothetical policies	-	-	-	Two-weeks in fall 2008 and two weeks in fall 2009	Six high traffic locations on campus, during mid-day on all five days of the week
Wiium (2009)	Support for hypothetical policies	-	-	-	October 4 to 23, 2004	List of randomly selected 16–20-year-olds from the Norwegian Population Register
Wipfli (2020)	Hypothetical support	-	-	-	10 months in 2017	Online survey through Qualtrics Survey Software sent to on campus list servers of all 50 partners of the Association of Pacific

						Rim Universities (APRU) Global Health Program (GHP).
Wong (2020)	Support for hypothetical policies	-	-	-	January to August 2018	Campus community members, Recruitment via print directories (undergraduate students), online directories (graduate students, faculty), listservs (postdoctoral, fellows, staff). Random digit table to select and email survey.
Wray (2020)	Tobacco free campus in Missouri, USA. Prohibited use of tobacco at all campuses indoor and outdoor, parking facilities, university housing and hotels/establishments operated by the university	July 1st 2016	Community (University)	Not reported	17-06-16 till 30-06-16 and one year later same period	Email to all University faculty, staff and students.
Xiao (2020)	Support for hypothetical policies	-	-	-	2015	Non-institutionalised men and women age 15 and older, handheld computers were used to collect data

Appendix V – MMAT scores per study

Author	1. Is the sampling strategy relevant to address the research question?	2. Is the sample representative of the target population?	3. Are the measurements appropriate?	4. Is the risk of Nonresponse bias low?	5. Is the statistical analysis appropriate to answer the research question?
**Al-Delaimy (2008)**	Yes	Yes	Yes	Yes	Yes
**Abundis (2008)**	Yes	Can't tell	Yes	Can't tell	Can't tell
**Agaku (2014)**	Yes	Yes	Yes	Yes	Yes
**Almutairi (2014)**	Yes	Yes	Yes	Yes	Yes
**Atiba (2020)**	Yes	Can't tell	Yes	Can't tell	Yes
**Bartington (2020)**	Yes	Yes	Yes	No	Yes
**Basto-Abreu (2016)**	Yes	Can't tell	Yes	Yes	Yes
**Berg (2015)**	Yes	Yes	Yes	Yes	Yes
**Berg (2016)**	Yes	Yes	Yes	No	Yes
**Berg (2020)**	Yes	Yes	Yes	No	Yes
**Boeckmann (2018)**	Yes	Yes	Yes	Can't tell	Yes
**Bo-Woo (2012)**	Yes	Yes	Yes	Can’t tell	Yes
**Bower (2005)**	Yes	Yes	Yes	Can't tell	Can't tell
**Braverman (2015)**	Yes	Can't tell	Yes	No	Yes
**Braverman (2021)**	Yes	Can't tell	Yes	No	Yes
**Brown (2019)**	Yes	Yes	Yes	Yes	Yes
**Burns (2013)**	Yes	Yes	Yes	No	Yes
**Buth (2013)**	Yes	Can’t tell	Yes	Can’t tell	Yes
**Cartanyà-Hueso (2019)**	Yes	Yes	Yes	Can't tell	Yes
**Chaaya (2021)**	Yes	Yes	Yes	Yes	Yes
**Clegg (2021)**	Yes	Can’t tell	Yes	Can’t tell	Yes
**Crosby (2018)**	Yes	Can’t tell	Yes	Can’t tell	Yes
**Díez-Izquierdo (2017)**	Yes	Can't tell	Yes	No	Yes
**Dono (2014)**	Yes	Yes	Yes	Yes	Yes
**Drach (2010)**	Yes	Yes	Yes	Yes	Yes
**Dunn (2008)**	Yes	Yes	Yes	Yes	Yes
**El Ansari (2012a)**	Yes	Yes	Yes	Yes	Yes
**El Ansari (2012b)**	Yes	Yes	Yes	Yes	Yes
**El Ansari (2021)**	Yes	Can’t tell	Yes	No	Yes
**Fallin (2015)**	Yes	Yes	Yes	Can’t tell	Yes
**Farran (2021)**	Yes	Yes	Yes	Yes	Yes
**Fong (2013)**	Yes	Yes	Yes	Yes	Yes
**Gallus (2012)**	Yes	Yes	Yes	Can't tell	Yes
**Garg (2011)**	Yes	Yes	Yes	Can’t tell	Yes
**Gendall (2013)**	Yes	Yes	Yes	Can't tell	Yes
**Gentzke (2018)**	Yes	Can't tell	Yes	Yes	Yes

Author	1. Is the sampling strategy relevant to address the research question?	2. Is the sample representative of the target population?	3. Are the measurements appropriate?	4. Is the risk of Nonresponse bias low?	5. Is the statistical analysis appropriate to answer the research question?
**Gillespi (2005)**	Yes	Yes	Yes	Yes	Yes
**Hale (2017)**	Yes	Yes	Yes	Can’t tell	Yes
**Hammond (2006)**	Yes	Yes	Yes	Yes	Yes
**Hewett (2012)**	Yes	Yes	Yes	No	Yes
**Ickes (2017)**	Yes	No	Yes	Can't tell	Yes
**Jalleh (2006)**	Yes	Can't tell	Yes	Can't tell	Yes
**Johnston Polacek (2008)**	Yes	Yes	Yes	Can’t tell	Yes
**Kandra (2007)**	Yes	Yes	Yes	Yes	Yes
**Kandra (2013)**	Yes	Yes	Yes	Yes	Yes
**Karadağ (2021)**	Yes	Can’t tell	Yes	Yes	Yes
**Kecojevic (2020)**	Yes	Can’t tell	Yes	No	Yes
**Kennedy (2012)**	Yes	Yes	Yes	Yes	Yes
**Kennedy (2015)**	Yes	Yes	Yes	No	Yes
**King (2010)**	Yes	Yes	Yes	Yes	Yes
**King (2014)**	Yes	Yes	Yes	Yes	Yes
**Klein (2007)**	Yes	Yes	Yes	Can't tell	Yes
**Kruger (2015)**	Yes	Can’t tell	Yes	Can’t tell	Yes
**Kruger (2016)**	Yes	Yes	Yes	Yes	Yes
**Lechner (2012)**	Yes	Yes	Yes	Can’t tell	Yes
**Li (2013)**	Yes	Yes	Yes	Yes	Yes
**Li (2016)**	Yes	Yes	Yes	Yes	Yes
**Licht (2012)**	Yes	Yes	Yes	No	Yes
**Lin (2006)**	Yes	Yes	Yes	No	Yes
**Loukas (2006)**	Yes	No	Yes	Can't tell	Yes
**Lugo (2017)**	Yes	Yes	Yes	Can't tell	Yes
**Lund (2016)**	Yes	Can't tell	Yes	Can't tell	Yes
**Mamudu (2016)**	Yes	Can’t tell	Yes	Can’t tell	Can’t tell
**Marsh (2014)**	Yes	Yes	Yes	Yes	Yes
**Martínez-Sánchez (2014)**	Yes	Yes	Yes	Can't tell	Yes
**McMillen (2004)**	Yes	Yes	Yes	Yes	Yes
**McMillen (2019)**	Yes	Yes	Yes	Yes	Yes
**Meng (2016)**	Yes	Yes	Yes	Yes	Can't tell
**Mishra (2011)**	Yes	Yes	Yes	Can't tell	Yes
**Morain (2013)**	Yes	Yes	Yes	Yes	Yes
**Niemeier (2014)**	Yes	Yes	Yes	Can’t tell	Yes
**Nogueira (2022)**	Yes	Yes	Yes	Yes	Yes
**Okoli (2013)**	Yes	Can’t tell	Yes	Can't tell	Yes
**Patel (2022)**	Yes	Yes	Yes	Yes	Yes

Author	1. Is the sampling strategy relevant to address the research question?	2. Is the sample representative of the target population?	3. Are the measurements appropriate?	4. Is the risk of Nonresponse bias low?	5. Is the statistical analysis appropriate to answer the research question?
**Pederson (2016)**	Yes	Can’t tell	Yes	Can't tell	Yes
**Ramachandran (2020)**	Yes	Can’t tell	Yes	Yes	Yes
**Rashid (2014)**	Yes	Can't tell	Yes	Can't tell	Yes
**Reuband (2014)**	Yes	Yes	Yes	Can’t tell	Yes
**Rhoades (2019)**	Yes	Yes	Yes	Yes	Yes
**Riad-Allen (2017)**	Yes	Yes	Yes	Can't tell	Yes
**Rodríguez-González (2021)**	Yes	Can’t tell	Yes	Yes	Yes
**Rosen (2012)**	Yes	Can't tell	Yes	Can't tell	Yes
**Rosenberg (2012)**	Yes	Yes	Yes	Yes	Yes
**Ruokolainen (2018)**	Yes	Yes	Yes	Yes	Yes
**Sabrian (2019)**	Yes	Can't tell	Yes	Can't tell	Yes
**Sæbø (2019)**	Yes	Yes	Yes	No	Yes
**Schmidt (2016)**	Yes	Can't tell	Yes	No	Yes
**Sendal (2021)**	Yes	Yes	Yes	Yes	Yes
**Seo (2005)**	Yes	Yes	Yes	Can't tell	Yes
**Stevenson (2008)**	Yes	Can't tell	Yes	Yes	Yes
**Stillman (2018)**	Yes	Yes	Yes	Yes	Yes
**Sureda (2015)**	Yes	Yes	Yes	Yes	Yes
**Sweeting (2021)**	Yes	Yes	Yes	Can’t tell	Yes
**Thompson (2006)**	Yes	Yes	Yes	Yes	Yes
**Topuridze (2020)**	Yes	Yes	Yes	Yes	Yes
**Unrod (2012)**	Yes	Yes	Yes	Can't tell	Yes
**Waddell (2014)**	Yes	Yes	Yes	No	Yes
**Wallar (2013)**	Yes	Yes	Yes	Can’t tell	Yes
**Walsh (2008)**	Yes	Yes	Yes	Yes	Yes
**Wang (2018)**	Yes	Yes	Yes	Yes	Yes
**Wheeler (2007)**	Yes	Yes	Yes	Yes	Yes
**Wiium (2009)**	Yes	Yes	Yes	Yes	Yes
**Williams (2011)**	Yes	Yes	Yes	Can’t tell	Yes
**Wipfli (2020)**	Can’t tell	No	Yes	No	Yes
**Wong (2020)**	Yes	Yes	Yes	No	Yes
**Wray (2020)**	Yes	Yes	Yes	No	Yes
**Xiao (2020)**	Yes	Yes	Yes	Yes	Yes

Appendix VI – Forest plots per type of location

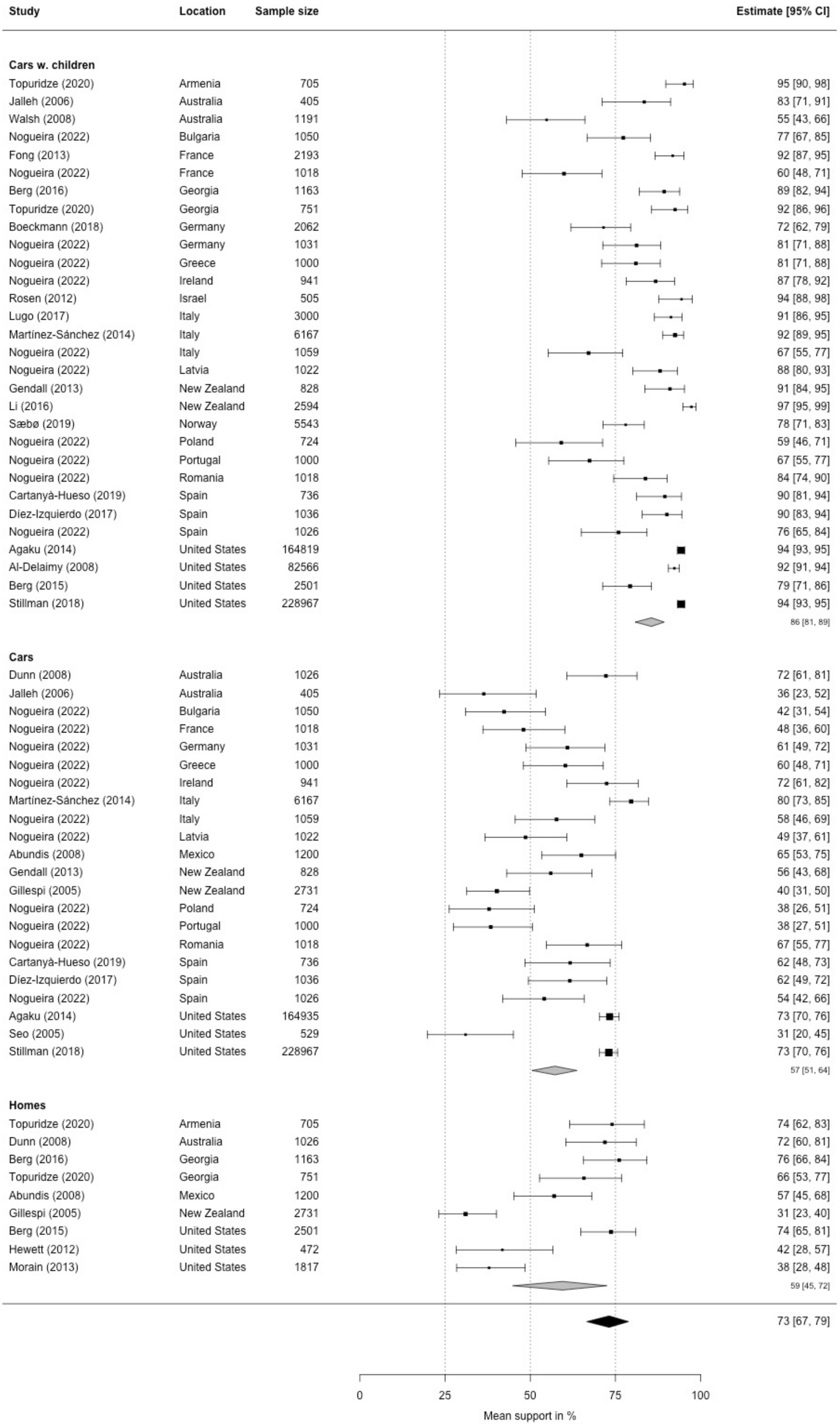

Figure 1: Support for indoor private places, I^2 is reported in Appendix VII

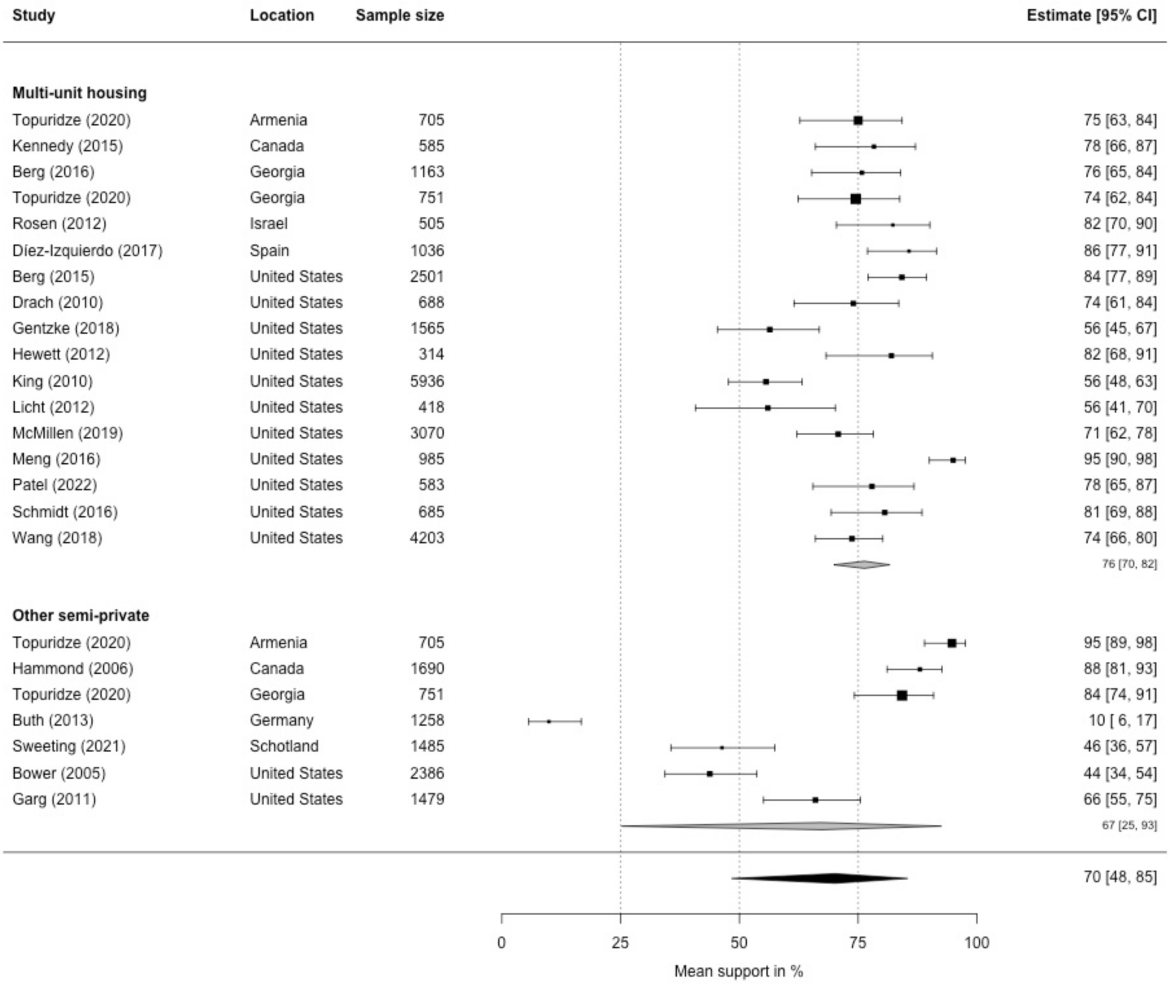

Figure 2: Support for indoor semi-private places, I^2 is reported in Appendix VII

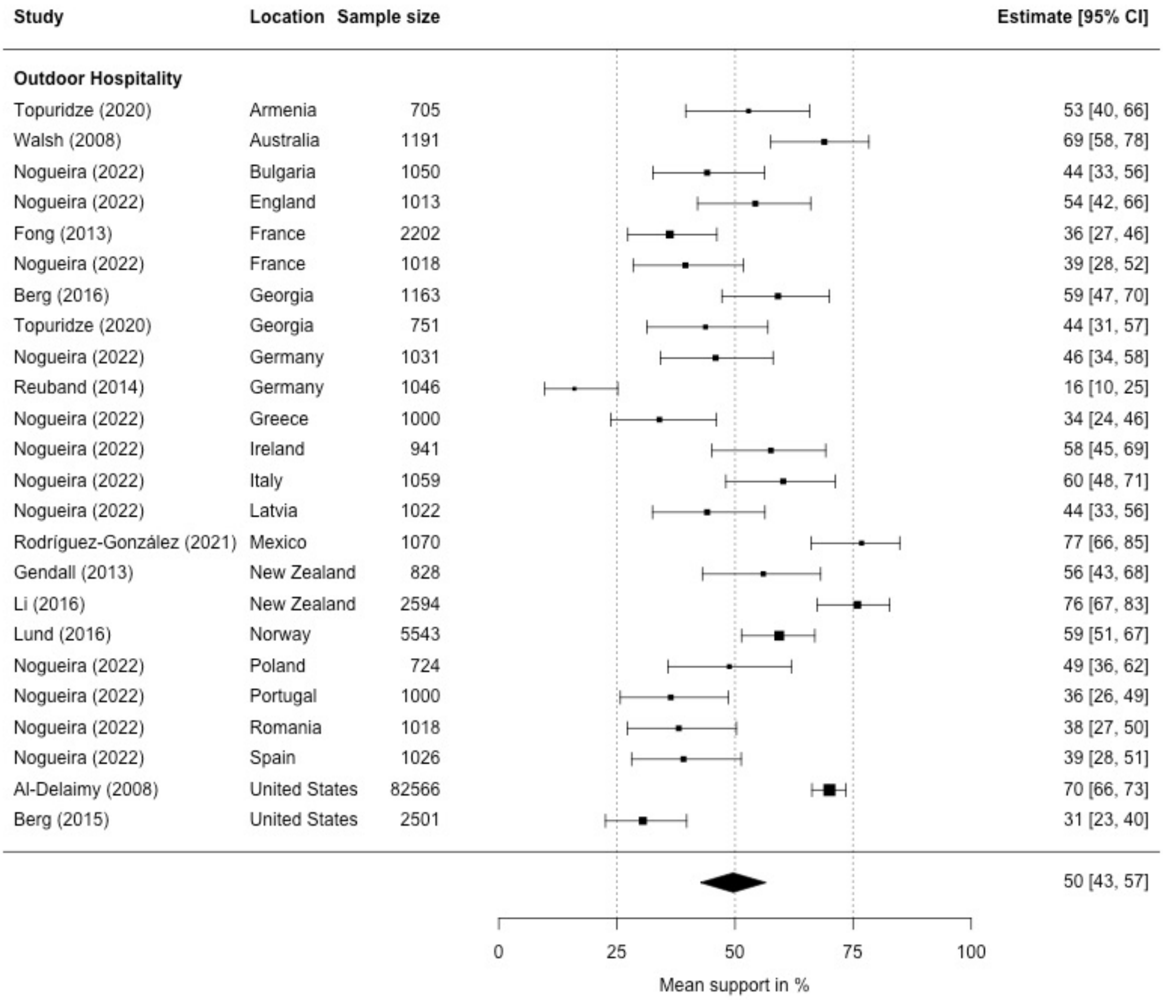

Figure 3: Support for outdoor hospitality places, I^2 is reported in Appendix VII

Study	Location	Sample size	Estimate [95% CI]
**Areas surrounding building entrances**			
Topuridze (2020)	Armenia	705	55 [42, 68]
Berg (2016)	Georgia	1163	74 [63, 83]
Topuridze (2020)	Georgia	751	61 [48, 73]
Gendall (2013)	New Zealand	828	65 [52, 76]
Li (2016)	New Zealand	2594	80 [73, 86]
Sæbø (2019)	Norway	5545	53 [45, 61]
Al-Delaimy (2008)	United States	82566	67 [63, 71]
Berg (2015)	United States	2501	79 [71, 86]
			68 [58, 76]
**Outdoor areas healthcare**			
Hale (2017)	Australia	1108	74 [63, 83]
Nogueira (2022)	Bulgaria	1050	54 [42, 66]
Riad-Allen (2017)	Canada	556	68 [54, 79]
Nogueira (2022)	England	1013	70 [58, 79]
Nogueira (2022)	France	1018	43 [31, 55]
Nogueira (2022)	Germany	1031	52 [40, 64]
Nogueira (2022)	Greece	1000	41 [30, 53]
Nogueira (2022)	Ireland	941	60 [48, 71]
Rosen (2012)	Israel	505	93 [85, 97]
Gallus (2012)	Italy	6233	80 [74, 85]
Martínez-Sánchez (2014)	Italy	6167	75 [68, 81]
Nogueira (2022)	Italy	1059	53 [41, 65]
Nogueira (2022)	Latvia	1022	70 [59, 80]
Nogueira (2022)	Poland	724	52 [39, 65]
Nogueira (2022)	Portugal	1000	53 [40, 65]
Nogueira (2022)	Romania	1018	67 [55, 77]
Cartanyà-Hueso (2019)	Spain	736	78 [66, 86]
Nogueira (2022)	Spain	1026	63 [51, 74]
Sureda (2015)	Spain	1301	53 [41, 64]
Clegg (2021)	United Kingdom	477	64 [49, 77]
Crosby (2018)	United Kingdom	934	91 [84, 95]
Lin (2006)	United States	1356	93 [87, 96]
Unrod (2012)	United States	511	82 [70, 90]
Wheeler (2007)	United States	842	83 [73, 90]
			68 [59, 75]
**Event locations**			
Rosenberg (2012)	Australia	2005	75 [66, 83]
Walsh (2008)	Australia	1191	77 [66, 85]
Nogueira (2022)	Bulgaria	1050	57 [45, 69]
Nogueira (2022)	England	1013	61 [48, 72]
Fong (2013)	France	2188	45 [35, 55]
Nogueira (2022)	France	1018	46 [34, 58]
Berg (2016)	Georgia	1163	66 [54, 76]
Nogueira (2022)	Germany	1031	37 [27, 50]
Nogueira (2022)	Greece	1000	42 [31, 55]
Nogueira (2022)	Ireland	941	63 [51, 74]
Gallus (2012)	Italy	6233	68 [61, 75]
Martínez-Sánchez (2014)	Italy	6167	67 [59, 73]
Nogueira (2022)	Italy	1059	54 [42, 66]
Nogueira (2022)	Latvia	1022	71 [60, 81]
Basto-Abreu (2016)	Mexico	796	82 [71, 89]
Gendall (2013)	New Zealand	828	68 [55, 78]
Wiium (2009)	Norway	2368	58 [48, 67]
Nogueira (2022)	Poland	724	56 [43, 69]
Nogueira (2022)	Portugal	1000	41 [30, 54]
Nogueira (2022)	Romania	1018	56 [44, 67]
Cartanyà-Hueso (2019)	Spain	736	65 [52, 76]
Nogueira (2022)	Spain	1026	48 [36, 60]
Sureda (2015)	Spain	1289	54 [42, 65]
Berg (2015)	United States	2501	71 [62, 79]
			60 [54, 66]
**Park & Beach**			
Topuridze (2020)	Armenia	705	72 [59, 82]
Walsh (2008)	Australia	1191	56 [44, 67]
Nogueira (2022)	Bulgaria	1050	42 [31, 54]
Okoli (2013)	Canada	500	85 [74, 92]
Nogueira (2022)	England	1013	57 [45, 69]
Nogueira (2022)	France	1018	44 [32, 56]
Topuridze (2020)	Georgia	751	55 [42, 68]
Nogueira (2022)	Germany	1031	42 [31, 54]
Nogueira (2022)	Greece	1000	40 [29, 52]

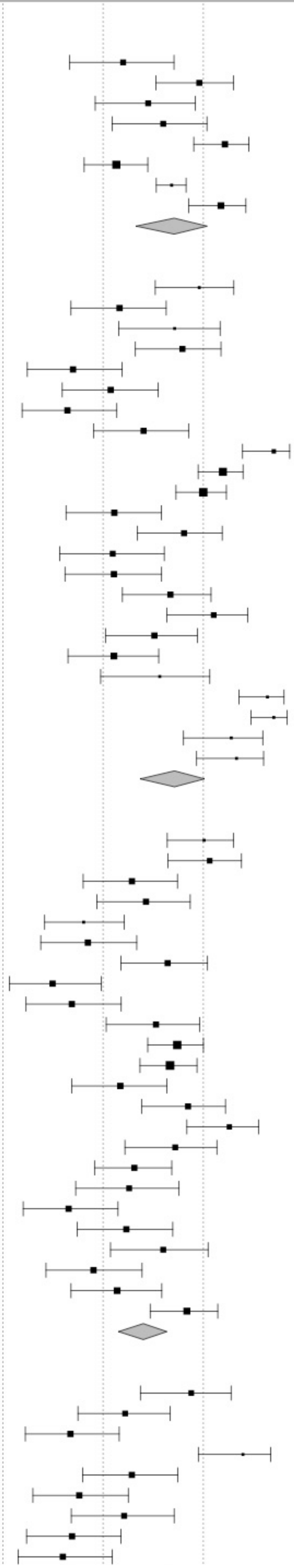

Nogueira (2022)	Ireland	941	55 [42, 67]
Rosen (2012)	Israel	505	30 [19, 44]
Gallus (2012)	Italy	6233	63 [56, 70]
Martínez-Sánchez (2014)	Italy	6167	61 [53, 68]
Nogueira (2022)	Italy	1059	52 [40, 64]
Nogueira (2022)	Latvia	1022	56 [44, 68]
Abundis (2008)	Mexico	1200	47 [36, 59]
Basto-Abreu (2016)	Mexico	796	85 [75, 91]
Gendall (2013)	New Zealand	828	62 [49, 73]
Stevenson (2008)	New Zealand	587	83 [72, 90]
Sæbø (2019)	Norway	5544	34 [27, 42]
Nogueira (2022)	Poland	724	50 [37, 63]
Nogueira (2022)	Portugal	1000	41 [29, 53]
Nogueira (2022)	Romania	1018	57 [45, 68]
Cartanyà-Hueso (2019)	Spain	736	50 [37, 63]
Nogueira (2022)	Spain	1026	45 [33, 57]
Sureda (2015)	Spain	1296	43 [32, 55]
Klein (2007)	United States	1488	62 [51, 72]
Kruger (2016)	United States	97978	38 [35, 42]
McMillen (2004)	United States	3008	37 [29, 47]
Waddell (2014)	United States	1446	49 [38, 60]
			53 [47, 59]
**Playground**			
Topuridze (2020)	Armenia	705	88 [80, 94]
Dono (2014)	Australia	2013	98 [95, 99]
Walsh (2008)	Australia	1191	89 [81, 93]
Nogueira (2022)	Bulgaria	1050	86 [77, 91]
Nogueira (2022)	England	1013	88 [80, 93]
Nogueira (2022)	France	1018	55 [42, 66]
Topuridze (2020)	Georgia	751	80 [69, 88]
Nogueira (2022)	Germany	1031	75 [64, 83]
Nogueira (2022)	Greece	1000	63 [51, 74]
Nogueira (2022)	Ireland	941	82 [72, 89]
Nogueira (2022)	Italy	1059	62 [50, 73]
Nogueira (2022)	Latvia	1022	89 [82, 94]
Gendall (2013)	New Zealand	828	82 [72, 89]
Nogueira (2022)	Poland	724	65 [52, 76]
Nogueira (2022)	Portugal	1000	67 [55, 77]
Nogueira (2022)	Romania	1018	83 [73, 89]
Cartanyà-Hueso (2019)	Spain	736	85 [75, 91]
Nogueira (2022)	Spain	1026	74 [63, 83]
Sureda (2015)	Spain	1301	81 [71, 88]
Stillman (2018)	United States	228967	88 [86, 89]
			81 [74, 86]
**Streets or open areas**			
Bo-Woo (2012)	Korea, Rep.	1013	79 [68, 86]
Rashid (2014)	Malaysia	2969	91 [86, 94]
Basto-Abreu (2016)	Mexico	796	84 [74, 91]
Gendall (2013)	New Zealand	828	61 [48, 73]
Cartanyà-Hueso (2019)	Spain	736	43 [30, 56]
Sureda (2015)	Spain	1298	38 [28, 50]
Rhoades (2019)	United States	4461	75 [68, 81]
			74 [53, 88]
**Public transport stops**			
Topuridze (2020)	Armenia	705	79 [68, 88]
Dono (2014)	Australia	2013	94 [89, 96]
Nogueira (2022)	Bulgaria	1050	42 [31, 54]
Nogueira (2022)	England	1013	64 [52, 75]
Nogueira (2022)	France	1018	40 [29, 52]
Berg (2016)	Georgia	1163	70 [59, 79]
Topuridze (2020)	Georgia	751	76 [64, 85]
Nogueira (2022)	Germany	1031	48 [36, 60]
Nogueira (2022)	Greece	1000	37 [26, 49]
Nogueira (2022)	Ireland	941	62 [50, 73]
Rosen (2012)	Israel	505	58 [44, 71]
Martínez-Sánchez (2014)	Italy	6167	60 [52, 67]
Nogueira (2022)	Italy	1059	53 [41, 64]
Bo-Woo (2012)	Korea, Rep.	1013	90 [83, 94]
Nogueira (2022)	Latvia	1022	66 [55, 77]
Li (2016)	New Zealand	2594	75 [66, 82]
Sæbø (2019)	Norway	5543	58 [50, 66]
Nogueira (2022)	Poland	724	51 [38, 64]
Nogueira (2022)	Portugal	1000	43 [31, 55]
Nogueira (2022)	Romania	1018	51 [39, 63]
Cartanyà-Hueso (2019)	Spain	736	66 [53, 77]
Nogueira (2022)	Spain	1026	47 [36, 60]
Sureda (2015)	Spain	1305	56 [45, 67]
Berg (2015)	United States	2501	72 [63, 80]
			63 [54, 71]

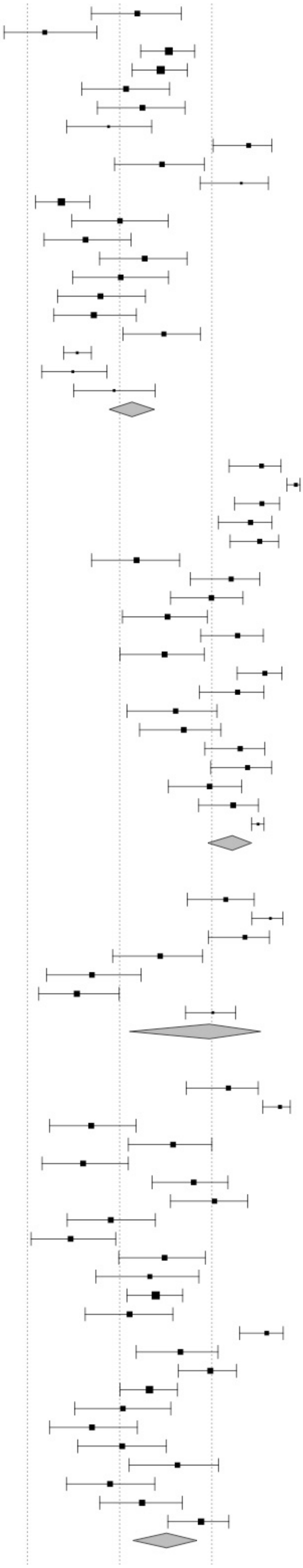

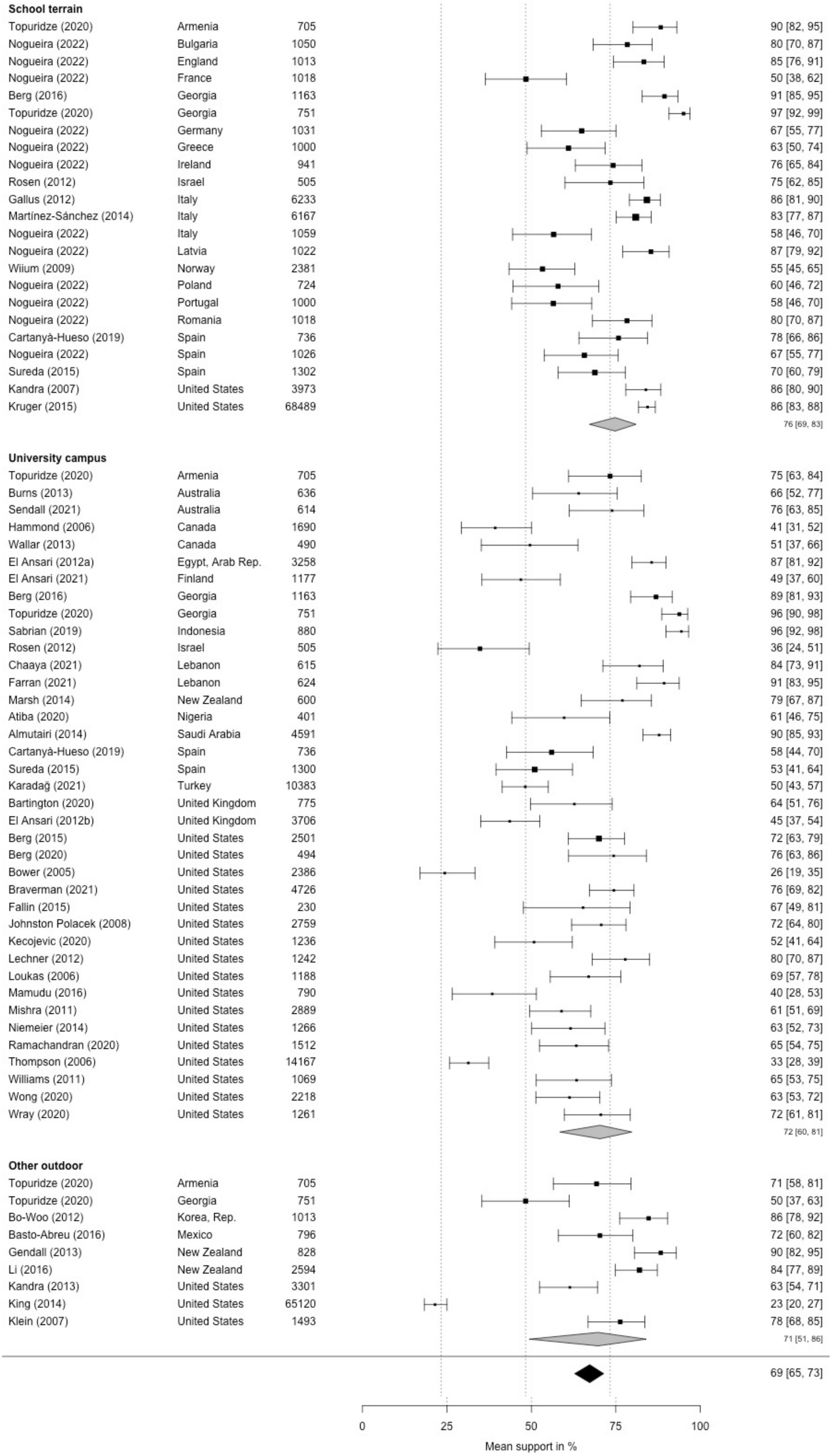
School terrain
Topuridze (2020) Armenia 705 90 [82, 95]
Nogueira (2022) Bulgaria 1050 80 [70, 87]
Nogueira (2022) England 1013 85 [76, 91]
Nogueira (2022) France 1018 50 [38, 62]
Berg (2016) Georgia 1163 91 [85, 95]
Topuridze (2020) Georgia 751 97 [92, 99]
Nogueira (2022) Germany 1031 67 [55, 77]
Nogueira (2022) Greece 1000 63 [50, 74]
Nogueira (2022) Ireland 941 76 [65, 84]
Rosen (2012) Israel 505 75 [62, 85]
Gallus (2012) Italy 6233 86 [81, 90]
Martínez-Sánchez (2014) Italy 6167 83 [77, 87]
Nogueira (2022) Italy 1059 58 [46, 70]
Nogueira (2022) Latvia 1022 87 [79, 92]
Wiium (2009) Norway 2381 55 [45, 65]
Nogueira (2022) Poland 724 60 [46, 72]
Nogueira (2022) Portugal 1000 58 [46, 70]
Nogueira (2022) Romania 1018 80 [70, 87]
Cartanyà-Hueso (2019) Spain 736 78 [66, 86]
Nogueira (2022) Spain 1026 67 [55, 77]
Sureda (2015) Spain 1302 70 [60, 79]
Kandra (2007) United States 3973 86 [80, 90]
Kruger (2015) United States 68489 86 [83, 88]
76 [69, 83]
University campus
Topuridze (2020) Armenia 705 75 [63, 84]
Burns (2013) Australia 636 66 [52, 77]
Sendall (2021) Australia 614 76 [63, 85]
Hammond (2006) Canada 1690 41 [31, 52]
Wallar (2013) Canada 490 51 [37, 66]
El Ansari (2012a) Egypt, Arab Rep. 3258 87 [81, 92]
El Ansari (2021) Finland 1177 49 [37, 60]
Berg (2016) Georgia 1163 89 [81, 93]
Topuridze (2020) Georgia 751 96 [90, 98]
Sabrian (2019) Indonesia 880 96 [92, 98]
Rosen (2012) Israel 505 36 [24, 51]
Chaaya (2021) Lebanon 615 84 [73, 91]
Farran (2021) Lebanon 624 91 [83, 95]
Marsh (2014) New Zealand 600 79 [67, 87]
Atiba (2020) Nigeria 401 61 [46, 75]
Almutairi (2014) Saudi Arabia 4591 90 [85, 93]
Cartanyà-Hueso (2019) Spain 736 58 [44, 70]
Sureda (2015) Spain 1300 53 [41, 64]
Karadağ (2021) Turkey 10383 50 [43, 57]
Bartington (2020) United Kingdom 775 64 [51, 76]
El Ansari (2012b) United Kingdom 3706 45 [37, 54]
Berg (2015) United States 2501 72 [63, 79]
Berg (2020) United States 494 76 [63, 86]
Bower (2005) United States 2386 26 [19, 35]
Braverman (2021) United States 4726 76 [69, 82]
Fallin (2015) United States 230 67 [49, 81]
Johnston Polacek (2008) United States 2759 72 [64, 80]
Kecojevic (2020) United States 1236 52 [41, 64]
Lechner (2012) United States 1242 80 [70, 87]
Loukas (2006) United States 1188 69 [57, 78]
Mamudu (2016) United States 790 40 [28, 53]
Mishra (2011) United States 2889 61 [51, 69]
Niemeier (2014) United States 1266 63 [52, 73]
Ramachandran (2020) United States 1512 65 [54, 75]
Thompson (2006) United States 14167 33 [28, 39]
Williams (2011) United States 1069 65 [53, 75]
Wong (2020) United States 2218 63 [53, 72]
Wray (2020) United States 1261 72 [61, 81]
72 [60, 81]
Other outdoor
Topuridze (2020) Armenia 705 71 [58, 81]
Topuridze (2020) Georgia 751 50 [37, 63]
Bo-Woo (2012) Korea, Rep. 1013 86 [78, 92]
Basto-Abreu (2016) Mexico 796 72 [60, 82]
Gendall (2013) New Zealand 828 90 [82, 95]
Li (2016) New Zealand 2594 84 [77, 89]
Kandra (2013) United States 3301 63 [54, 71]
King (2014) United States 65120 23 [20, 27]
Klein (2007) United States 1493 78 [68, 85]
71 [51, 86]
69 [65, 73]
0
25
50
75
100
Mean support in %

Figure 4: Public support for outdoor non-hospitality places, variance reported, I^2 is reported in Appendix VII

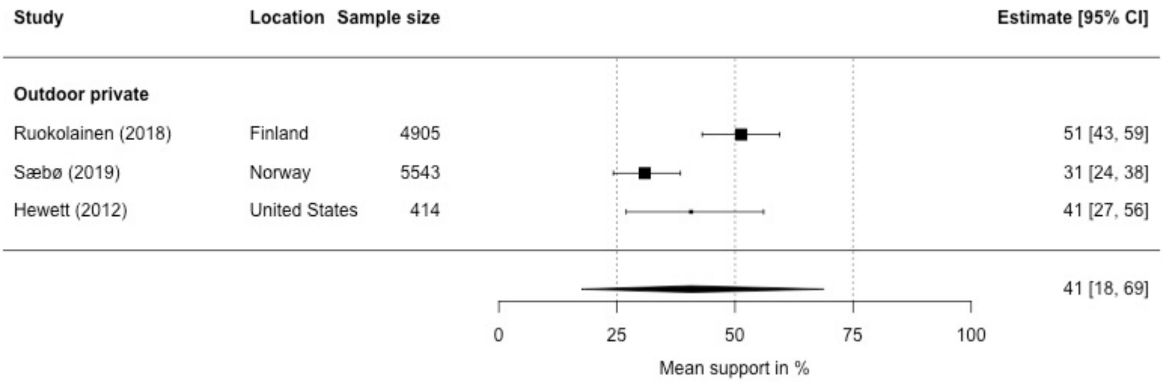

Figure 5: Public support for outdoor private places, I^2 is reported in Appendix VII

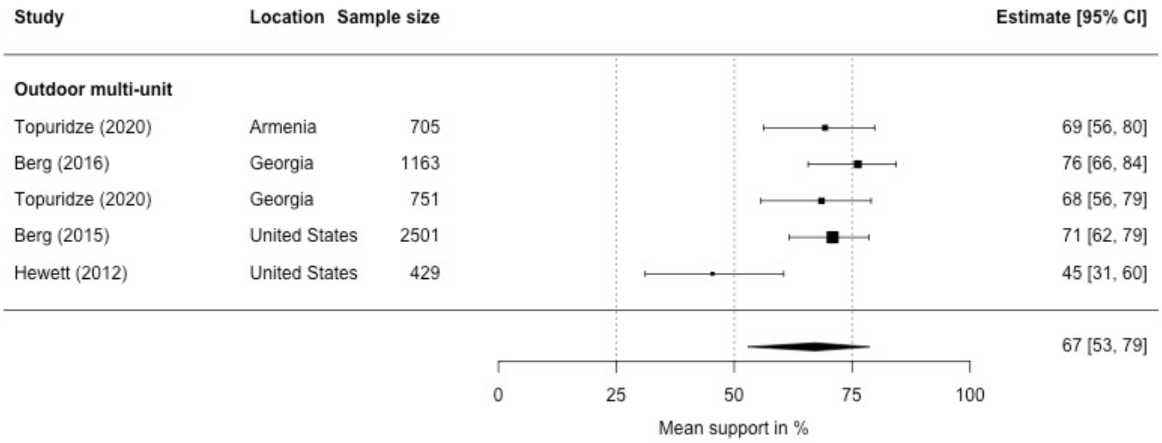

Figure 6: Public support for outdoor semi -private spaces, I^2 is reported in Appendix VII

Appendix VII – I^2 per type of location

Table VI.1 variance for three-level meta-analysis per type

	I^2
Indoor private	
Within country	95.2
Between country	0.0
Overall	95.2
Indoor semi-private	
Within country	29.6
Between country	66.4
Overall	96.0
Outdoor hospitality	
Within country	88.6
Between country	0.0
Overall	88.6
Outdoor non-hospitality	
Within country	90.5
Between country	2.3
Overall	92.8
Outdoor private	
Within country	40.3
Between country	40.3
Overall	80,6
Outdoor semi-private	
Within country	69.2
Between country	0,0
Overall	69.2

Table VI.2 variance for three-level meta-analysis per group

	I^2
Areas surrounding building entrances	
Within country	83,7
Between country	0,0
Overall	83,7
Cars w. children	
Within country	82,3
Between country	10,8
Overall	93,1
Cars	
Within country	89,8

Between country	0,0
Overall	89,8
Event locations	
Within country	5,1
Between country	74,7
Overall	79,8
Homes	
Within country	88,6
Between country	0,8
Overall	89,4
Multi-unit housing	
Within country	82,3
Between country	0,0
Overall	82,3
Other outdoor	
Within country	77,4
Between country	18,3
Overall	95,7
Other semi-private	
Within country	12,0
Between country	85,8
Overall	97,8
Outdoor areas healthcare	
Within country	58,3
Between country	31,1
Overall	89,4
Outdoor hospitality	
Within country	88,6
Between country	0,0
Overall	88,6
Outdoor multi-unit	
Within country	69,2
Between country	0,0
Overall	69,2
Outdoor private	
Within country	40,3
Between country	40,3
Overall	80,6
Park & Beach	
Within country	67,0
Between country	20,0
Overall	87,0
Playground	
Within country	45,5
Between country	43,9

Overall	89,4
Public transport stops	
Within country	3,6
Between country	87,3
Overall	90,9
School terrain	
Within country	33,9
Between country	56,9
Overall	90,8
University campus	
Within country	28,5
Between country	66,7
Overall	95,2
Streets or open areas	
Within country	0,0
Between country	93,1
Overall	93,1

Appendix VIII – Meta-regression including variable for hypothetical support

Table VI: Meta-regression hypothetical studies vs non-hypothetical

Area of policies	Variable	OR	95%CI	p-value
Indoor private (n = 58)				
	Study year, per year increase	1.01	(0.93-1.09)	.818
	Number of smoke-free policies in place	0.93	(0.83-1.03)	.169
	Low- and middle-income level*	1.53	(0.61-3.88)	.359
	Hypothetical support**	0.17	(0.04-0.67)	**.012**
Indoor semi-private (n = 24)				
	Study year. per year increase	1.07	(0.97-1.17)	.196
	Number of smoke-free policies in place	1.19	(0.94-1.54)	.219
	Low- and middle-income level*	1.02	(0.09-11.78)	.988
	Question type***	1.63	(0.83-3.19)	.144
	Hypothetical support**	2.99	(1.17-7.66)	**.025**
Outdoor hospitality (n = 24)				
	Study year. per year increase	0.93	(0.86-1.01)	.079
	Number of smoke-free policies in place	1.05	(0.95-1.17)	.308
	Low- and middle-income level*	1.24	(0.61-2.55)	.525
	Hypothetical support**	0.32	(0.08-1.20)	.085
Outdoor non-hospitality (n = 204)				
	Study year. per year increase	0.97	(0.92-1.01)	.149
	Number of smoke-free policies in place	1.02	(0.97-1.09)	.421
	Low- and middle-income level*	2.29	(1.36-3.86)	**.002**
	Hypothetical support**	0.50	(0.39-0.62)	**.000**
	Question type***	1.01	(0.41-2.49)	.976

* ref = High income level

** ref = Not hypothetical

*** ref = Binary question

Appendix IX – Sensitivity analysis

Table VII: pooled estimates for studies at risk of bias versus at low risk of bias

	At risk of bias	Low risk of bias	p-value
Indoor private	74% (63-83)	73% (64-80)	.833
Indoor semi-private	68% (47-83)	74% (53-88)	.412
Outdoor private	34% (26-43)	51% (40-63)	**.018**
Outdoor semi-private	63% (44-78)	70% (56-81)	.531
Outdoor hospitality	53% (39-67)	49% (41-56)	.608
Outdoor non-hospitality	71% (65 -77)	67% (61 -73)	.332

Appendix X- Funnel plots of study size against log odds of support

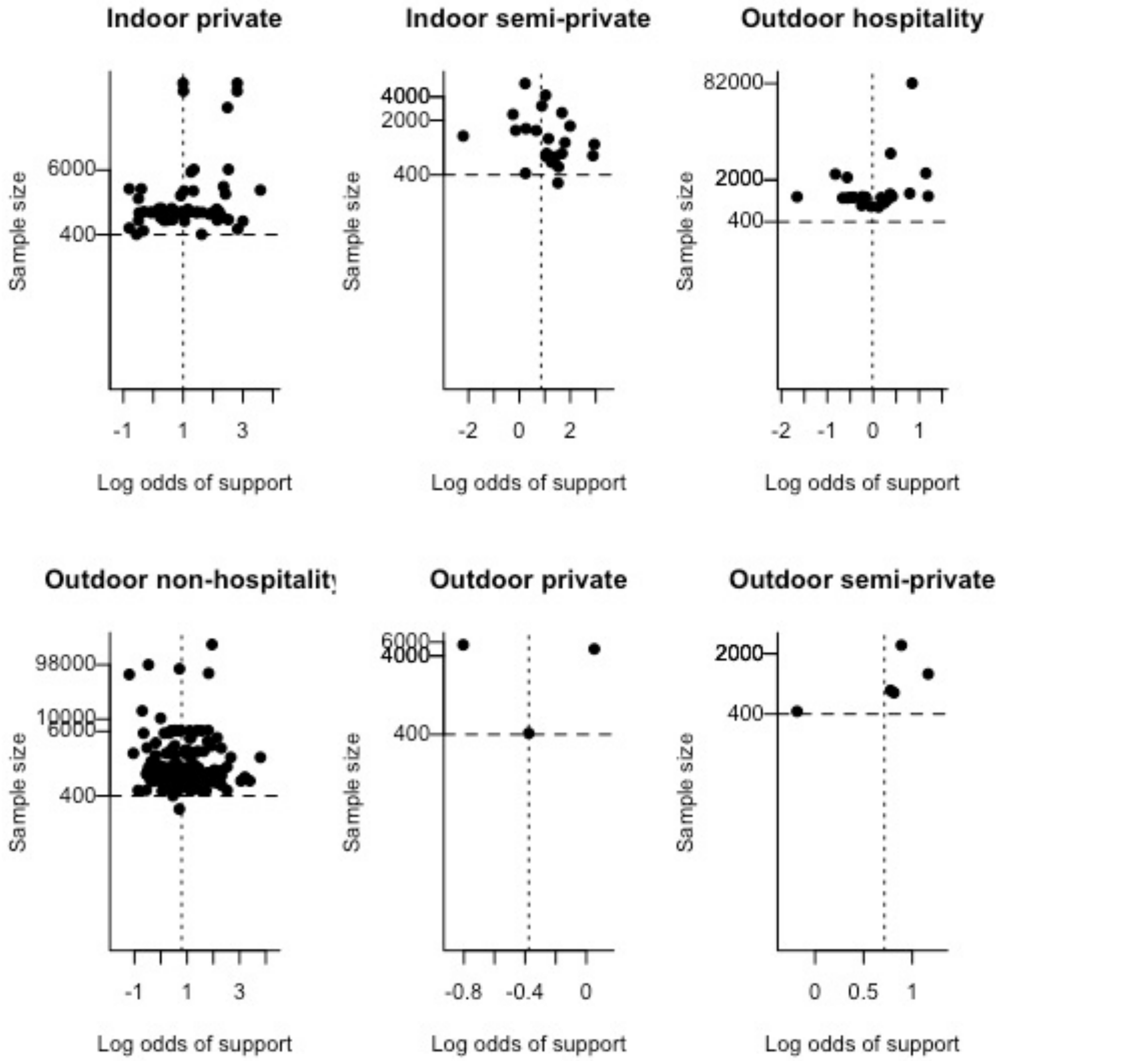

Figure 1: Funnel plot of sample size against log odds of support, per type of location. Vertical reference line indicates pooled estimate per group, horizontal indicates the minimum sample size required for inclusion

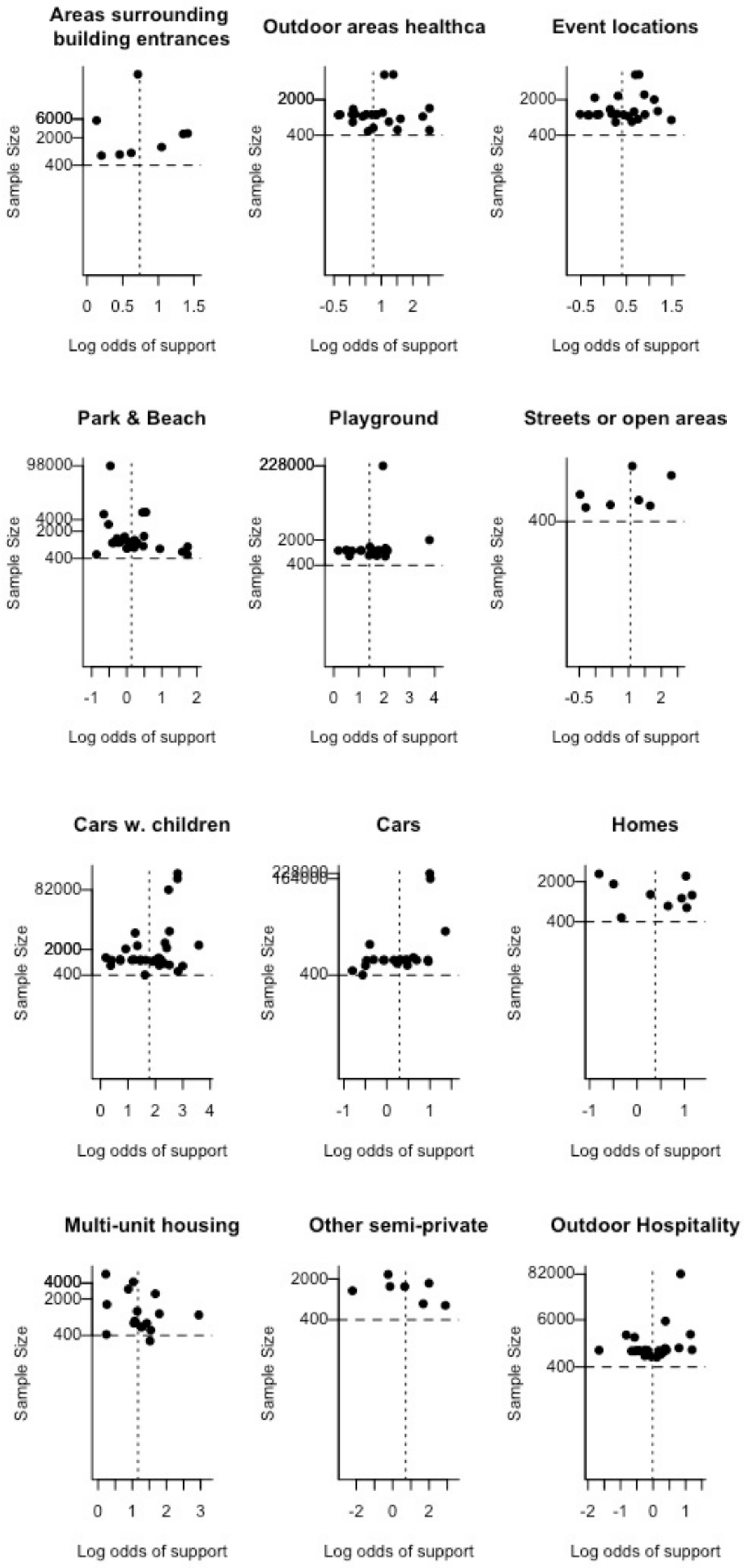

Areas surrounding building entrances
Sample Size
6000
2000
400
0 0.5 1 1.5
Log odds of support
Outdoor areas healthca
Sample Size
2000
400
-0.5 1 2
Log odds of support
Event locations
Sample Size
2000
400
-0.5 0.5 1.5
Log odds of support
Park & Beach
Sample Size
98000
4000
2000
400
-1 0 1 2
Log odds of support
Playground
Sample Size
228000
2000
400
0 1 2 3 4
Log odds of support
Streets or open areas
Sample Size
400
-0.5 1 2
Log odds of support
Cars w. children
Sample Size
82000
2000
400
0 1 2 3 4
Log odds of support
Cars
Sample Size
228000
164000
400
-1 0 1
Log odds of support
Homes
Sample Size
2000
400
-1 0 1
Log odds of support
Multi-unit housing
Sample Size
4000
2000
400
0 1 2 3
Log odds of support
Other semi-private
Sample Size
2000
400
-2 0 2
Log odds of support
Outdoor Hospitality
Sample Size
82000
6000
400
-2 -1 0 1
Log odds of support

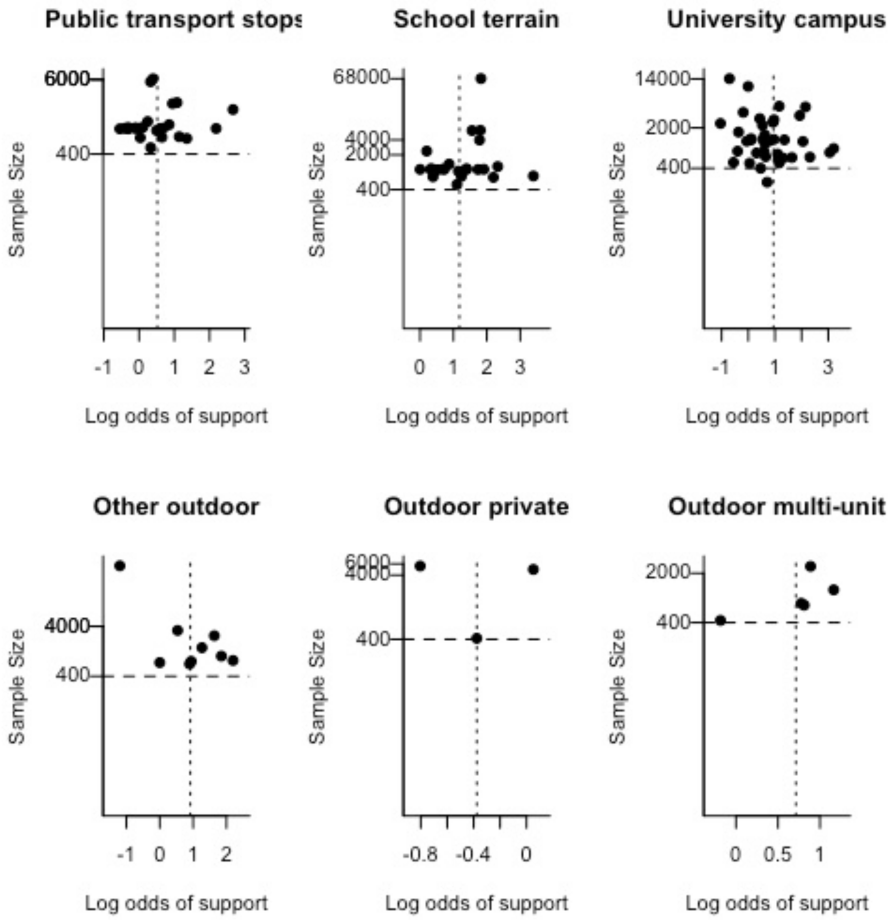

Figure 2: funnel plot of sample size against log odds of support per group of locations. Vertical reference line indicates pooled estimate per group, horizontal indicates the minimum sample size required for inclusion.

Appendix XI– Studies exclude at full text screening

Exclusion reason	Citation
Conference paper or review	(Appleby 2004, Duffin 2005, Thomson, Wilson et al. 2005, Wilson 2005, Burhan, Ryder et al. 2007, Schmidt 2007, Shipley and Allcock 2007, Tang and Phoenix 2008, Thomson, Wilson et al. 2008, Mills, Messer et al. 2009, Thomson and Wilson 2009, Thomson, Wilson et al. 2009, Cho and DeVaney 2010, Danishevskiy, McKee et al. 2010, De Lacy, Morrison et al. 2010, Economo, Stewart et al. 2010, Gilroy, Clarke et al. 2010, Jarrold, Huntly et al. 2010, Marques-Vidal, Cerveira et al. 2010, Shrestha and Amatya 2010), 2012), (Cordoba Garcia and Sanz Andres 2012, Gilroy, Doherty et al. 2012, Gilroy, Doherty et al. 2012, Ravara, Braganc¸a et al. 2013, Ravara, Braganca et al. 2013, Jiang and Ling 2014, Filippidis, Agaku et al. 2015, Lupton and Townsend 2015, 2016, Farley, Schroth et al. 2016, Hayran, Aherrera et al. 2016, Snyder, Vick et al. 2016, Thomson, Wilson et al. 2016, Díez-Izquierdo, Lidón-Moyano et al. 2017, Jaine, Edwards et al. 2017, Rees, Davine et al. 2017, Thomson, Martin et al. 2017, Asare, Owusu et al. 2018, Atiba, Odukoya et al. 2018, Boeker, Carter et al. 2018, Galiatsatos, Koehl et al. 2018, Jiang, Sansone et al. 2018, Jin, Song et al. 2018, Ningsih 2018, Nnadiwa, Kotey et al. 2018, Perez, Chung-Hall et al. 2018, Rutter, Lloyd et al. 2018, Van Dooremaal, Haandrikman et al. 2018, Wen and Zhao 2018, Woodall and Tattersfield 2018, Wu, Wang et al. 2018, McNeill, Krabill et al. 2019, Mochizuki, Sansone et al. 2019, Brown, Eadie et al. 2020, Do, Fallavollita et al. 2020, Rogers, Barrington-Trimis et al. 2020, Clegg, Howle et al. 2021)
Study recruited less than 400 participants	(Ratschen, Britton et al. 2008, Chadzyński, Woźniak et al. 2009, Fitzpatrick, Gilroy et al. 2009, Butler, Rayens et al. 2012, Ballor, Henson et al. 2013, Hood, Ferketich et al. 2013, McIntosh, Collins et al. 2015, Stockings, Bowman et al. 2015, Barrios, Ramírez et al. 2016, Feldman, Donchin et al. 2016, Li, Nelson et al. 2016, Precioso, Reis et al. 2016, Rokicki, Adamkiewicz et al. 2016, Khan, Hira et al. 2017, Huddlestone, Sohal et al. 2018, Babu, Indiran et al. 2019, Bommele, Troelstra et al. 2020, Pignataro and Daramola 2020, Yasin, Zubillah et al. 2020, Al-Jayyousi, Kurdi et al. 2021)
Sample not representative for intervention	(King, Mallett et al. 2005, Vinnikov, Burzhubaeva et al. 2009, Wernz, Friederich et al. 2009, Nieminen, Toljamo et al. 2010, Baezconde-Garbanati, Weich-Reushe et al. 2011, Bonevski, Paul et al. 2011, Gelen, Köksal et al. 2011, Mamudu, Veeranki et al. 2012, Thomson and Wilson 2012, Cook, Hollar et al. 2014, Cooper, Cabriales et al. 2016, Braverman, Hoogesteger et al. 2017, Wilson, Torok et al. 2017, Debchoudhury and Farley 2019)
Measure of support not reported as proportion of support or not for novel smoke-free policy alone	(Awotedu, Awotedu et al. 2006, Fong, Hyland et al. 2006, Doucet, Velicer et al. 2007, Lemstra, Neudorf et al. 2008, Chen, Huang et al. 2009, Franca, Dautzenberg et al. 2009, García-Vázquez, Arbesú Fernández et al. 2009, Lazuras, Rodafinos et al. 2009, Prochaska, Burdine et al. 2009, Marques-Vidal, Melich-Cerveira et al. 2010, Saglam, Bayraktar et al. 2010, Vardavas, Dimitrakaki et al. 2011, Clemente Jiménez, Bartolomé Moreno et al. 2012, Cakir, Buzgan et al. 2013, Council, Wilson et al. 2013, Dresler, Wei et al. 2013, Yang, Abdullah et al. 2013, Berg, Haardörfer et al. 2015, Dao, Hoang et al. 2015, Hall, Williams et al. 2015, McMillen, Wilson et al. 2018, Saqib, Rafique et al. 2018, Jackson-Morris 2019, Sweeting, Semple et al. 2019, Cheung, Romero et al. 2020, Haddad, Sacre et al. 2020, Rossheim, Zhao et al. 2020, Glasgow, Miller et al. 2021)
No measure of support reported for a novel smoke-free policy	(Ahmed, Aujayeb et al. 2004, Shanahan, Scheufele et al. 2004, Ceylan, Yanik et al. 2005, Gong-huan, Jie-min et al. 2005, Hersch 2005, 2006, Borland, Yong et al. 2006, Cross, Green et al. 2006, Gallus, Pacifici et al. 2006, Saltó, Joan et al. 2006, Saltó, Villalbí et al. 2006, Dwyer, Bradshaw et al. 2008, Larsson, Boëthius et al. 2008, Rayens, Hahn et al. 2008, Brown, Moodie et al. 2009, Mei, Ma et al. 2009, Parks, Wilson et al. 2009, Riou França, Dautzenberg et al. 2009, Smith, Siebel et al. 2009, Szmagaj and Jóźwiak 2009, Abdullah, Yang et al. 2010, Kowalewska 2010, Li, Hyland et al. 2010,

	Martínez-Sánchez, Fernández et al. 2010, Osypuk and Acevedo-Garcia 2010, Thrasher, Besley et al. 2010, Thrasher, Pérez-Hernández et al. 2010, Thyrian, Panagiotakos et al. 2010, A and A 2011, Amador and Nicolás 2011, Lewis, Shin et al. 2011, Noland, Rayens et al. 2011, Pacheco 2011, Tachfouti, El Rhazi et al. 2011, Thrasher, Huang et al. 2011, Atilla, Köksal et al. 2012, Nagelhout, de Vries et al. 2012, Radwan, Emam et al. 2012, Radwan, Loffredo et al. 2012, Raptou, Galanopoulos et al. 2012, Badillo Amador and López Nicolás 2013, Bakhturidze, Mittelmark et al. 2013, Biedma-Velazquez, Espinosa-Monteros et al. 2013, Chaaya, Alameddine et al. 2013, Jannu, Jha et al. 2013, Yang, Abdullah et al. 2013, Jenkins, Fakhoury et al. 2014, Lykke, Helbech et al. 2014, Olowookere, Adepoju et al. 2014, Fong, Sansone et al. 2015, Nagelhout, Wolfson et al. 2015, Forden and Carrillo 2016, Nan, Xi et al. 2016, Obeidat, Ayub et al. 2016, Obeidat, Ayub et al. 2016, Niederdeppe, Kellogg et al. 2018, Hock, Hui Li et al. 2019, Sansone, Fong et al. 2019, Gambaryan and Drapkina 2020, Grossberg, Loukas et al. 2020, Holmes, Beard et al. 2020, Lathen, Plears et al. 2020, Mamudu, Owusu et al. 2020, Rogers, Barrington-Trimis et al. 2020, Sendall, Le Lievre et al. 2020, Anwar and Senosy 2021, Asut, Vaizoglu et al. 2021, Ibrahim, Sabitu et al. 2021, Toxværd, Pisinger et al. 2021, Ngoc Yen, Obeid et al. 2022, Sæbø and Lund 2022)
No measure of support reported	(Gerson, Allard et al. 2005, Freeman, Chapman et al. 2008, Edwards, Wilson et al. 2009, Seward and Martin 2009, Alday, Murukutla et al. 2010, Thomson, Wilson et al. 2010, Ye, Gao et al. 2010, Berg, Lessard et al. 2011, Gajendra, Ossip et al. 2011, Gorini 2011, Jackson and Bonnie 2011, Kawabe 2011, Walsh, Paul et al. 2011, Abdullah, Hua et al. 2012, Klein, Bernat et al. 2012, Villaverde Royo, Marín Izaguerri et al. 2012, Zhou, Gao et al. 2012, Edwards, Wilson et al. 2013, Pacheco 2013, Gallus, Lugo et al. 2014, King, Patel et al. 2014, Serafin, Franklin et al. 2014, Hashimoto, Makinodan et al. 2015, Zhang and Martinez-Donate 2015, Bich, Cook et al. 2016, Lund, Halkjelsvik et al. 2016, Pagano, Guydish et al. 2016, Filippidis, Girvalaki et al. 2017, Guillaumier, Bonevski et al. 2017, Hollar, Cook et al. 2017, Hummel, Willemsen et al. 2017, Lee, Purcell et al. 2017, McCrabb, Baker et al. 2017, Chung-Hall, Fong et al. 2018, Seitz, Kabir et al. 2018, Volinsky, Kranzler et al. 2018, Ickes, Butler et al. 2019, Rozema, Mathijssen et al. 2019)
Policy regarding a tobacco product subgroup	(Martínez-Sánchez, Ballbé et al. 2014, Brose, McNeill et al. 2017, Brose, Partos et al. 2017, Cheung, Wang et al. 2017, Gorukanti, Delucchi et al. 2017, Odani, O'Flaherty et al. 2018)
(Former) smokers only	(Thomson, Weerasekera et al. 2009, Thomson, Wilson et al. 2009, Wilson, Blakely et al. 2009, Wilson, Weerasekera et al. 2010, Hitchman, Fong et al. 2011, Fu, Castellano et al. 2018, Park, Cho et al. 2019, Sohlberg 2019, Driezen, Fong et al. 2020, Nogueira, Tigova et al. 2020, Siddiqi, Siddiqui et al. 2020, Edwards, Johnson et al. 2021)
